# Genome Implosion Elicits Host-Confinement in *Alcaligenaceae*: Evidence from the Comparative Genomics of *Tetrathiobacter kashmirensis,* a Pathogen in the Making

**DOI:** 10.1371/journal.pone.0064856

**Published:** 2013-05-31

**Authors:** Wriddhiman Ghosh, Masrure Alam, Chayan Roy, Prosenjit Pyne, Ashish George, Ranadhir Chakraborty, Saikat Majumder, Atima Agarwal, Sheolee Chakraborty, Subrata Majumdar, Sujoy Kumar Das Gupta

**Affiliations:** 1 Department of Microbiology, Bose Institute, Kolkata, West Bengal, India; 2 Invitrogen Bioservices India, Gurgaon, Haryana, India; 3 Department of Biotechnology, University of North Bengal, Siliguri, West Bengal, India; 4 Division of Molecular Medicine, Bose Institute, Kolkata, India; 5 Central Instrument Facility, Bose Institute, Kolkata, India; Cornell University, United States of America

## Abstract

This study elucidates the genomic basis of the evolution of pathogens alongside free-living organisms within the family *Alcaligenaceae* of *Betaproteobacteria*. Towards that end, the complete genome sequence of the sulfur-chemolithoautotroph *Tetrathiobacter kashmirensis* WT001^T^ was determined and compared with the soil isolate *Achromobacter xylosoxidans* A8 and the two pathogens *Bordetella bronchiseptica* RB50 and *Taylorella equigenitalis* MCE9. All analyses comprehensively indicated that the RB50 and MCE9 genomes were almost the subsets of A8 and WT001^T^, respectively. In the immediate evolutionary past *Achromobacter* and *Bordetella* shared a common ancestor, which was distinct from the other contemporary stock that gave rise to *Tetrathiobacter* and *Taylorella*. The *Achromobacter*-*Bordetella* precursor, after diverging from the family ancestor, evolved through extensive genome inflation, subsequent to which the two genera separated via differential gene losses and acquisitions. *Tetrathiobacter*, meanwhile, retained the core characteristics of the family ancestor, and *Taylorella* underwent massive genome degeneration to reach an evolutionary dead-end. Interestingly, the WT001^T^ genome, despite its conserved architecture, had only 85% coding density, besides which 578 out of its 4452 protein-coding sequences were found to be pseudogenized. Translational impairment of several DNA repair-recombination genes in the first place seemed to have ushered the rampant and indiscriminate frame-shift mutations across the WT001^T^ genome. Presumably, this strain has just come out of a recent evolutionary bottleneck, representing a unique transition state where genome self-degeneration has started comprehensively but selective host-confinement has not yet set in. In the light of this evolutionary link, host-adaptation of *Taylorella* clearly appears to be the aftereffect of genome implosion in another member of the same bottleneck. Remarkably again, potent virulence factors were found widespread in *Alcaligenaceae*, corroborating which hemolytic and mammalian cell-adhering abilities were discovered in WT001^T^. So, while WT001^T^ relatives/derivatives in nature could be going the *Taylorella* way, the lineage as such was well-prepared for imminent host-confinement.

## Introduction

The family *Alcaligenaceae* is a phylogenetically coherent assemblage of environmentally as well as physiologically distinct betaproteobacteria. Its members range from the ecologically versatile *Achromobacter* and *Alcaligenes* [1], [2], [3], [4], [5] to soil chemolithoautotrophs like *Tetrathiobacter kashmirensis* (recently reclassified as *Advenella kashmirensis*) [6], [7], and at the same time include pathogens like species of *Bordetella*, *Taylorella* etc. [8], [9], [10], [11]. In 16S rRNA gene sequence-based phylogeny most of the branches of *Alcaligenaceae* juxtapose organisms isolated from human, animal as well as environmental samples. While genera like *Bordetella*, *Achromobacter*, *Alcaligenes*, *Pelistega*, *Taylorella* etc. all have members isolated from within animal bodies, *Tetrathiobacter*, *Pigmentiphaga*, *Castellaniella* etc., appear to be composed of truly environmental organisms apparently not associated with human or animal diseases. Understanding the differential evolution of pathogenic and non-pathogenic variants within phylogenetically coherent bacterial groups is an area of significant contemporary interest [12], [13]. So we thought it would be very important to appreciate the genomic basis of the occurrence of parasitism/pathogenicity hand in hand with free-living ability in taxonomically close members of *Alcaligenaceae*.

Despite an early start, comparative genomic studies with *Alcaligenaceae*, for a long time, revolved around the mammalian parasites *Bordetella bronchiseptica* (its strain RB50 is studied here in detail referred to hereafter as *Bb*), *Bordetella pertussis* (*Bp*) and *Bordetella parapertussis* (*Bpp*), plus the avian pathogen *Bordetella avium* (*Ba*) [8], [10], [14], [15], [16]. A clear trend of narrowing down of host range with plummeting genome size was proven among the sequenced *Bordetella* genomes [8]. Among these four species, *Bb*, which infects the widest range of mammalian hosts (including humans), had the largest genome of 5,339,179 base pairs (bp) [10]. *Bpp* with its two subpopulations, one containing isolates from cases of human whooping cough (*B*. *parapertussis* hu) and the other containing strains isolated from sheep (*B*. *parapertussis* ov) [16], had the next largest, 4,773,551 bp, genome [10]. On the other hand, the genome of *Bp*, which is restricted to human hosts causing pertussis or whooping cough, is 4,086,186 bp long [10], and that of *Ba*, the causative agent of bordetellosis in wild or domesticated birds, was found to be only 3,732,255 bp [8]. As such, relatively recent divergence of the two clonal species *Bp* and *Bpp* was propounded to have taken place from a distinct human-associated lineage of *B*. *bronchiseptica* via massive gene loss [8], [14], [15]. At the same time, apparent acquisition of novel genes was postulated as central to the development of exclusive host-specific adaptations in *Ba* that has more than 1,100 unique genes in comparison to *Bb* [8].

Despite all these developments in the understanding of *Bordetella* evolution there was no elucidation regarding the origin of *B*. *bronchiseptica*, which appears to be closest to the last common ancestor (LCA) of all bordetellae by virtue of its ability to persist freely in the environment, infect the widest range of mammalian hosts (including humans) and possession of the largest (and apparently uncut) genome (5.3 Mb) among sequenced *Bordetella* species [8]. Origin of parasitism/pathogenicity (or cases of reductive evolution) is often best understood by studying abridged genomes in comparison with their living relatives that have largely maintained the gene pool of the LCA [13]. Understandably, dearth of whole genome information in the non-pathogenic half of the family was the main reason behind the lack of knowledge on the origin of pathogenic *Alcaligenaceae*. This lacuna has lately been overcome with the publication of the 7,013,095 bp complete genome of the haloaromatic acid-degrading soil isolate A8 of *Achromobacter xylosoxidans* (*Ax*) [17] (referred to hereafter only as A8) and the ∼4.4 Mb shotgun genome of *Tetrathiobacter kashmirensis* WT001^T^ [18] (referred to hereafter as *Tk*), along with several other closely related genomes. While these data paved the way for a broad phylogenomic analysis of *Alcaligenaceae*, the 1.7 Mb genome of the contagious equine metritis-causing *Taylorella equigenitalis* MCE9 [11] (referred to hereafter as *Te*), also released in recent times, defined the lower size-limit of sequenced *Alcaligenaceae* genomes. Building upon these developments we completed the whole genome sequence of *Tk* and attempted a comparative analysis of the genome contents and architectures of some non-pathogenic (viz., A8 and *Tk*) and pathogenic (viz., *Bb* and *Te*) variants of this group. By identifying shared as well as species-specific genes we tried to explain the similarities and differences in their metabolic aptitudes and ecological adaptations. We also tried to detect the potential events of gene loss, gene acquisition and genome rearrangement involved in the divergence of these bacteria. In the process we asked whether *Alcaligenaceae* members can be clubbed along non-pathogenic and pathogenic lines on the basis of categorical genomic trends. It was further inquired whether the differentially adapted *Alcaligenaceae* diverged independently from their putative common ancestor(s) via separate lines of descent or some of them represent evolutionary intermediates. At length, we retraced the evolutionary history of the four *Alcaligenaceae* and tried to elucidate the phylogenomic basis of the origin of pathogenicity in the different lineages of the family.

## Results and Discussion

### General Characteristics of the *Tk* Genome

The complete *Tk* genome was found to encompass a 4,365,995 bp circular chromosome (sequence deposited in the EMBL/GenBank database under the accession no. CP003555) and a 57,884 bp circular plasmid pWTk445 (EMBL/GenBank accession CP003556), which is almost identical with the partially sequenced IncP plasmid pBTK445 of another strain WGT of *T*. *kashmirensis* [19]. G+C content of the *Tk* chromosome (54.9%) was found significantly lower than that of A8 or *Bb* (Table 1), while that of the plasmid pWTk445 was still lower (46.7%). However, the G+C content of neither the *Tk* chromosome nor its plasmid was as low as that of *Te* (37.42%).

**Table 1:** General features of the four studied genomes.

Genomic parameters	A8	*Bb*	*Tk*	*Te*
Genome size (bp)	7,013,095 (chromosome*)	5,339,179 (chromosome)	4,365,995 (chromosome)	1,695,860 (chromosome)
	98,156 (plasmid pA81)		57884 (plasmid pWTk445)	
	247,895 (plasmid pA82)			
GC content (%)	66.0 (chromosome)	68.1 (chromosome)	54.9 (chromosome)	37.4 (chromosome)
	62.2 (plasmid pA81)		46.7 (plasmid pWTk445)	
	61.3 (plasmid pA82)			
Genes	6532 (chromosome)	5072 (chromosome)	4503 (chromosome)	1603 (chromosome)
	108 (plasmid pA81)		61 (plasmid pWTk445)	
	254 (plasmid pA82)			
PEGs/CDSs	6459 (chromosome)	4994 (chromosome)	4452 (chromosome)	1556 (chromosome)
	104 (plasmid pA81)		61 (plasmid pWTk445)	
	252 (plasmid pA82)			
No. of putatively functional genes in the chromosome*	4874	3623	3341	1321
Number of predictedpathway variants governedby the chromosome	467	480	442	292
tRNAs	60 (chromosome)	55 (chromosome)	41 (chromosome)	38 (chromosome)
rRNAs	9 (chromosome)	9 (chromosome)	6 (chromosome)	9 (chromosome)
Pseudogenes	2 (chromosome)	18 (chromosome)	578 (chromosome)	1 (chromosome)
	4 (plasmid pA81)		0 (plasmid pWTk445)	
	2 (plasmid pA82)			
Coding area (%)	91 (chromosome)	92 (chromosome)	85 (chromosome)	93 (chromosome)
	90 (plasmid pA81)		82 (plasmid pWTk445)	
	77 (plasmid pA82)			
Phages-related genes	29 (chromosome)	230 (chromosome)	12 (chromosome)	4
	0 (plasmid pA81)		1 (plasmid pWTk445)	
	1 (plasmid pA82)			
Transposases/Integrases	22 (chromosome)	14 (chromosome)	12 (chromosome)	3
	13 (plasmid pA81)		3 (plasmid pWTk445)	
	1 (plasmid pA82)			

GenBank accession numbers: *A*. *xylosoxidans* A8 chromosome, CP002287; plasmid pA81, CP002288; plasmid pA82, CP002289. *B. bronchiseptica* RB50 (*Bb*) chromosome, NC_002927. *T*. *kashmirensis* WT001^T^ (*Tk*) chromosome, CP003555; plasmid pWTk445, CP003556. *T. equigenitalis* MCE9 (*Te*) chromosome, CP002456.

* This is equal to the number of genes involved in the constitution of complete metabolic subsystems or pathway variants.

The *Tk* chromosome encompasses 4503 genes, out of which 4456 are protein-encoding genes (PEGs). There are two copies each of the three rRNA genes organized in two paralogous gene clusters (having 100% mutual sequence identity), plus 41 tRNA genes distributed throughout the chromosome. pWTk445, in its turn, encodes 61 putative proteins, out of which 11 have no homolog in the database, three are integrases/transposases, five are transcriptional regulators, and one is a prophage-related protein. Other than its own replication and partition machineries (for which five genes could be attributed), an 11-gene Type IV secretion system (T4SS) and a two-gene UV tolerance and mutagenic DNA repair system (*umuDC* homolog) appeared to be the only functions encoded completely by the plasmid. Besides these, seven components of the *tra* locus characteristic of IncP plasmids could be identified alongside 17 more PEGs associated with a wide variety of basic metabolic processes such as lactate utilization (lactate to pyruvate), ribonucleotide reduction etc. Notably, 28 out of the 61 PEGs borne on pWTk445 have at least one homologous copy in the chromosome, with the number of such chromosomal counterpart ranging from one (as in case of the PEGs for autotransporter adhesin, maltose operon transcriptional repressor MalR, UDP-3-O-acyl-N-acetylglucosamine deacetylase, TraL and VirB6) to as many as 125 (as observed for the putative exported protein belonging to the extra-cytoplasmic solute receptor family COG3181). In contrast, 22 pWTk445 PEGs (including nine T4SS genes, three replication/partition genes, two integrase/transposase genes, one prophage- and six *tra*-related genes) have no other copy in the *Tk* genome. Notably however, T4SS homologs are chromosome-borne in *Bb* and *Te*, while in A8 they are located in the plasmid pA81 (Figure A in File S1). These facts collectively indicate that in the evolutionary past pWTk445, could have well been a part of the *Tk* chromosome.

Two striking feature of the *Tk* genome are its exceptionally low coding area percentage and the occurrence of unusually high number (578) of PEGs having potential frameshifts. Given the high level of read accuracy and coverage achieved in the sequencing and assembly of the *Tk* genome these are very unlikely to be sequencing errors. As such, a large majority of these frame-shifted coding sequences (CDSs) could be pseudogenes. In contrast, A8, *Bb* and *Te* all have significantly fewer pseudogenes and relatively higher coding densities.

### Genome Inflation as an Important Driver of *Alcaligenaceae* Evolution

The close taxonomic relationship of A8, *Bb*, *Tk* and *Te* is corroborated by their comparable gene contents. The four genomes encode a large number of similar (BLASTP E-values <10^−5^) protein sequences, many of which have orthologous relationships. At >25% sequence identity level, 78% (3496/4503) of *Tk* gene models aligned with those from A8, while 64% (4159/6532) of A8 counterparts aligned with *Tk* genes. Among these pairs, 2637 were reciprocal best hits, and hence likely to be orthologs. At the same time, 74% (3343/4503) of *Tk* genes aligned with *Bb* counterparts whereas 69% (3485/5072) of *Bb* genes aligned with those of *Tk*. In all 2373 *Tk-Bb* pairs were reciprocal best hits. On the other hand, 81% (5264/6532) of A8 gene models aligned with those from *Bb* and 84% (4264/5072) of *Bb* genes aligned with A8 counterparts, with 3419 A8*-Bb* pairs being bidirectional best hits. At >60% identity level, 31% (1410/4503) of *Tk* gene models aligned with those from A8, whereas just 22% (1408/6532) of A8 counterparts aligned with *Tk*’s (with 1344 reciprocal best hits). 27% (1230/4503) of *Tk* genes aligned with *Bb* counterparts, corroborating which 24% (1241/5072) of *Bb* genes aligned with those of *Tk* (in this case 1177 *Tk-Bb* pairs were reciprocal best hits). In contrast to the two relationships of *Tk*, 44% (2853/6532) of A8 gene models aligned with those from *Bb* and 56% (2850/5072) of *Bb* genes aligned with A8 counterparts, with the number of bidirectional best hits being 2774. Retrieval of more reciprocal best hits in *Tk-*A8 comparisons than in case of *Tk-Bb* implies that the gene content of *Tk* is more similar to that of A8 than *Bb*. With much higher numbers of mutual reciprocal best hits at both >25 and >60% identity levels, the A8*-Bb* relationship is clearly the closest, followed by *Tk-*A8 and *Tk-Bb*.

Similar analyses for *Te* indicated that its genome is more similar to *Tk* than to *Bb* or A8, with the latter pair being almost equidistant to *Te*. At >25% identity levels, 75% (1194/1603) of its predicted genes aligned with those from A8, while 27% (1733/6532) of A8 counterparts aligned back to *Te* homologs. Amongst these pairs, 1118 were reciprocal best hits. 74% (1180/1603) of *Te* genes also aligned with *Bb* counterparts whereas 32% (1630/5072) of *Bb* genes aligned with those from *Te.* 1111 *Te-Bb* pairs were reciprocal “best hits”. On the other hand, 76% (1209/1603) of *Te* genes aligned with *Tk* counterparts whereas 36% (1596/4503) of *Tk* genes aligned with those from *Te.* In all, 1137 *Te-Tk* pairs were found to be reciprocal “best hits”. At >60% sequence identity levels, 27% (433/1603) of *Te* gene models aligned with those from A8, while only 7% (448/6532) of A8 counterparts aligned with those from *Te.* Amongst these pairs, 432 were reciprocal best hits. ∼27% (429/1603) of *Te* genes also aligned with *Bb* counterparts whereas 9% (449/5072) of *Bb* genes aligned with those of *Te.* In all 428 *Te-Bb* pairs were reciprocal best hits. 31% (500/1603) of *Te* genes aligned with *Tk* counterparts and 12% (514/4503) of *Tk* genes aligned with those of *Te.* In all 498 *Te-Tk* pairs were reciprocal best hits.

One remarkable aspect of the above data is that at >25% identity level, the number of hits retrieved on comparing the gene content of a larger genome against that of a relatively smaller one is always more than the number of hits retrieved the other way round (i.e., on comparing a smaller genome against a bigger one). However, at >60% identity levels the number of hits retrieved in both ways of comparison remain more or less the same. This implies that in any pair-wise comparison net excess of shared paralogous genes in the larger genome accounts significantly for its relatively inflated genome size. As such, ∼60% (∼1 Mb out of the total 1.7 Mb) size difference between the A8 and *Bb* genomes can be attributed to the presence (in the A8 genome) of 1000 net extra copies of shared paralogous genes [1000 X 965 bp (the average length of A8 genes) = 0.965 Mb ≈ 1 Mb]. On the other hand, only 24% and 14% of the size differences between the A8 and *Tk* [∼0.64 Mb out of ∼2.7 Mb difference; this estimate is based on the fact that A8 has 663 net additional copies of shared paralogous genes in comparison to *Tk*], and *Bb* and *Tk* [only ∼0.14 Mb out of ∼1 Mb difference; this estimate is based on the fact that *Bb* possesses 142 net additional copies of shared paralogous genes in comparison to *Tk* and the average gene length of *Bb* is 983 bp] genomes can be attributed to excess gene paralogy. Similarly, with respect to the tiny *Te* genome, net excess of paralogous genes in A8, *Bb* and *Tk* can account for only ∼10% (∼0.52 Mb out of 5.3 Mb difference), ∼12% (∼0.44 Mb of 3.6 Mb difference) and ∼12% (∼0.32 Mb of 2.6 Mb difference) of their respective inflated genome sizes.

**Figure 1:**
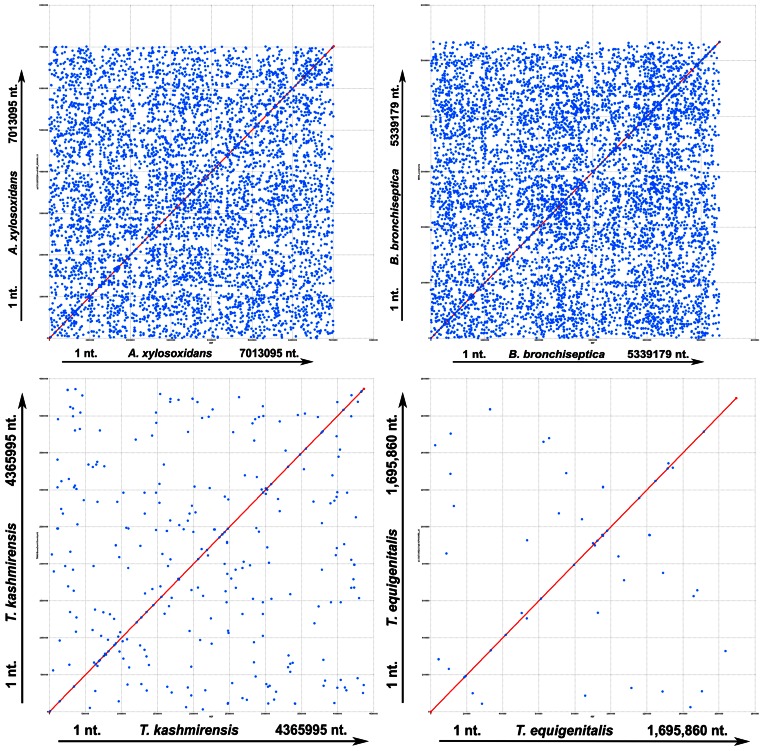
Dot-plot comparison of the four studied genomes against themselves using mummer. The *x*- and *y*-axes represent the same genome. Nucleotide numbers along the chromosomes are plotted along the axes from the origin onwards. All the unique maximal exact matches of minimum nucleotide sequence lengths between reference and query sequences on both the forward and reverse strands were identified and all the match positions relative to the forward strand reported. Direct and inverted matches are represented in red and blue respectively. Multiplicated stretches in the genomes are represented by the numerous dots appearing in the plots in addition to the main undisrupted diagonal lines.

The key role of gene multiplication in the development of the A8 and *Bb* genomes was also confirmed when dot-plot comparison of the four genomes was done against themselves using the programs mummer (Figure 1) as well as promer (data not shown) from the MUMmer 3.0 package. Though not totally absent, abundance of multiplicated stretches in the *Tk* genome was found to be far less than that in A8 or *Bb*, while in *Te* it was still lower. In all these plots *x*- and *y*-axes represented the same genome; that is why undisrupted diagonal lines were generated as usual; but numerous dots representing identical sequences scattered all over the genomes additionally appeared in various degrees of abundance depending on the profusion of multiplicated stretches in the concerned genome.

### Limited Role of HGT in the Evolution of *Alcaligenaceae*

Presence of genes conserved in *Alcaligenaceae* alongside unique ones lacking any counterpart in the sequenced genomes of the family illustrates the partially mosaic nature of the *Tk* genome. More than 70% of the *Tk* gene models shared highest sequence similarity with homologs from within the family, whereas less than 30% showed highest homology with genes from phylogenetically distinct bacteria. Deviations from average G+C content facilitates the identification of recent gene acquisitions, as foreign DNA typically possesses lower G+C content. As such, a sum total of at least 111,549 bp genomic region (∼2.6% of the genome) distributed over 64 genomic loci and encompassing 129 gene models, including one tRNA and two phage-related genes, were predicted to be derived from horizontal gene transfer (HGT) by virtue of having G+C contents below 50% in tandem with rare codon usage (Table A in File S1). Interestingly, among the HGT-affected *Tk* gene loci TkWG_22890 and TkWG_22895 showed highest (46% and 54%) BLASTP hits with hypothetical proteins of the *Burkholderia* phage φ52237. At the same time, five more putative HGT products showed affinity with homologs from *Burkholderia*, thereby reiterating that at some point of evolution the *Tk* genome might have been infected by burkholderial phages and the two bacterial genera have had extensive exchange of genetic material.

HGT (attributed on the basis of having G+C contents below 61% in tandem with rare codon usage) contributed to at least 242,145 bp of genomic region (∼3.5% of the genome) of A8. This length is distributed over 111 genomic loci encompassing 247 gene models and includes three identical *rrn* operons, three transposases, nine integrases and three phage-related genes (Table B in File S1). One of these phage-related genes and 20 other putative foreign genes showed highest BLASTP hits with burkholderial homologs, thereby buttressing the above-envisaged close genomic ties between burkholderias and *Alcaligenaceae*.

Again, despite containing several phage-related genes, the extent of HGT in the *Bb* genome was largely comparable to that in *Tk*. As such, 151,861 bp genomic stretch (∼2.8% of the genome) encompassing 145 predicted gene models distributed over 43 genomic loci [and including three identical *rrn* operons and 26 phage–related genes] were identified as derived from HGT on the basis of G+C contents below 62% and rare codon usage (Table C in File S1). *Te*, on the other hand, had the maximum portion of its genome (∼7.2% or 122,362 bp) attributable to HGT. This included 136 gene models (one phage-related integrase and two phage repressors) distributed over 66 genomic loci (Table D in File S1). In all four cases HGT products were predicted on the basis of minimum 8–10% deviation from the average G+C content of the genome in question and/or more than 15–20% deviation from the average codon adaptation index of the genome.

In terms of dinucleotide composition, very little difference was observed between the low G+C regions and the average G+C regions of all the genomes in question. Notably again, a large majority of the PEGs conjectured to be HGT products did have homologs across the *Betaproteobacteria*. Moreover, many of those ‘foreign’ genes exhibited highest sequence similarity with betaproteobacterial counterparts, and more often than not with homologs from within *Alcaligenaceae*. These facts collectively implied that most of the putative foreign genes had been acquired long before the divergence of the four organisms.

### Recombination-driven Genome Reorganization

**Figure 2:**
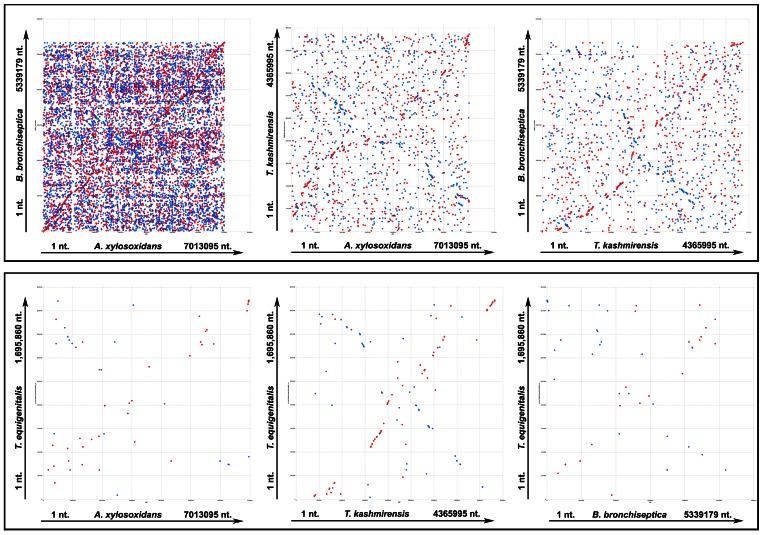
Pair-wise global co-linearity analysis between the studied genomes using mummer. The *x*- and *y*-axes represent the two genomes being compared. Nucleotide numbers along the chromosomes are plotted along the axes from the origin onwards. Maximal exact matches of minimum nucleotide sequence lengths between two genomes were identified. All maximal unique matches between reference and query sequences on both the forward and reverse strands were recognized and all the match positions relative to the forward strand reported. In order to get correct comparative pictures, the GenBank-retrieved genome sequences of *B. bronchiseptica* RB50 and *T. equigenitalis* MCE9 were reorganized before these analyses so as to make *dnaA* the first gene. Direct and inverted matches are represented in red and blue respectively.

Global as well as local co-linearity analyses were done to assess the contribution of genome rearrangements in the evolution of the four taxonomically close *Alcaligenaceae*. These data could also be used as a measure of relative affinity between the compared genomes. The comparable levels of pair-wise co-linearity observed in mummer (Figure 2) as well as promer (data not shown) plots iterated relatively closer relationships between A8 and *Bb*, and *Tk* and *Te*. It was also clear from these comparisons that the genome of *Tk*, or for that matter *Te*, was equidistant from A8 or *Bb*, even as *Tk* showed some semblance of closeness with the latter pair. The A8 versus *Bb* mummer comparison yielded a somewhat wobbly diagonal line interspersed with numerous disruptions along its length. The high frequency of interruption and disorder witnessed in this plot indicated umpteen number of recombination events, while the occurrence of multiple copies of several loci in either genome was evidenced by the mesh of dots appearing all over the plot area. Patterns witnessed in the other five plots were all the more haphazard, and involved fewer and shorter stretches of matching sequence. This observation pointed out the lack of significant genome-wide co-linearity between pairs other than A8-Bb. However, two extremely sketchy and interrupted diagonal lines were recognizable in the comparisons between *Tk* and A8, and *Tk* and *Bb*, while another imprecise diagonal line (accompanied by several interruptions reflecting multiple recombination events) also appeared in the *Tk* versus *Te* plot.

The above observations comprehensively imply that rampant recombination-driven genome reorganizations have played a central role in the recent divergence and evolution of these organisms. Repeated rearrangements have rendered extensive disarray in the genome architecture of these bacteria. Consequently, in several cases, genes expected to be co-localized in operon constructs have either been separated from each other or spiked by functionally unrelated genes. Whether these genes are at all functional in these bacteria, and if so how their functions are regulated, would be worth-exploring in the coming days.

**Figure 3:**
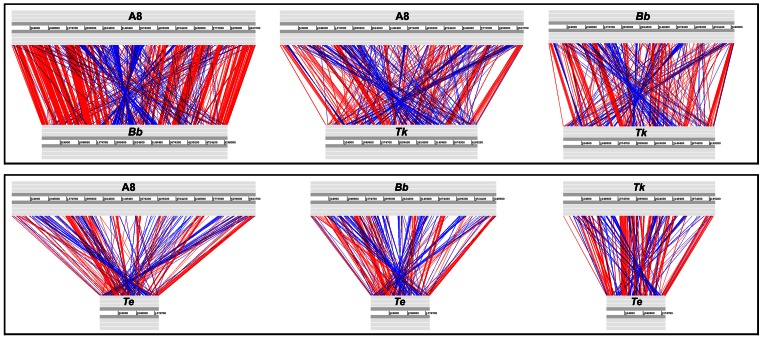
Protein-encoding genes shared pair-wise between the four studied genomes at ≥60% identity levels (TBLASTX). The gray bars represent forward and reverse DNA strands. Potential recombinatorial events between the genome pairs can be comprehended by comparing the red and blue lines that represent direct and inverted matches respectively. In order to get correct comparative pictures the GenBank-retrieved genome sequences of *Bb* and *Te* were reorganized before these analyses so as to make *dnaA* the first gene. **A8**, *A. xylosoxidans* A8; ***Bb***, *B. bronchiseptica* RB50; ***Tk***, *T*. *kashmirensis* WT001^T^; ***Te***, *T. equigenitalis* MCE9.

Pair-wise linear genomic comparison of similar translated protein sequences (TBLASTX with 70% average identity between orthologs) further elucidated the nature and extent of the recombination experienced by the four genomes (Figure 3). Random genomic arrangement of several shared orthologs (referred to as singletons or matching orthologs not adjacent to others) notwithstanding, local co-linearity [mutual order of arrangement with respect to the origin of replication (*ori*) of the genome] of an equally high number of gene clusters (syntenic regions) was found conserved in the compared genomes. Constituents of most of these conserved syntenic regions have also maintained their reading frame orientation with reference to *ori* (direct matches). Some of the conserved syntenies, however, involved opposite matches where collinear genes have reversed their reading frame orientation with respect to the *ori* but maintained their mutual arrangement. Remarkably again, if one discounts the additional segments of the larger genomes, a number of conserved syntenies (involving direct as well as opposite matches) appear to have maintained their location in the 360 degree genomic context. Examples of such positionally static loci include clusters involving (i) DNA gyrase subunit encoding genes, (ii) ribosomal genes, (iii) cell division genes, (iv) lipid A biosynthesis genes, (v) genes encoding Respiratory Complex I, (vi) the subunits B and A of topoisomerase IV etc. In another interesting case a conserved syntenic stretch (encompassing genes for the heat shock protein 60 family chaperone, bacterial signal recognition particle, *ssu* rRNA small subunit methyltransferase D, outer membrane lipoprotein LolB, glycerolipid and glycerophospholipid metabolism, thiamin biosynthesis, etc.) was found to have conserved ORF orientations as well as overall genomic localization in A8, *Bb* and *Tk*, but in *Te* localization of this segment and the orientation of the ORFs are both changed 180 degrees.

In terms of the total length of syntenic regions shared (direct as well as opposite matches involving two or more pairs of orthologs) as well as the number of singletons, A8 and *Bb* appeared to be closest to each other, whereas *Tk* was equidistant from both A8 and *Bb*. That the *Te* genome was closest to *Tk*, and equidistant from A8 or *Bb*, was also evident from the length as well as the arrangement of the pair-wise shared syntenic regions. Local co-linearity of two or more pairs of syntenic genes in several locations of the compared genomes reflected these relationships. For example the subunits B and A of topoisomerase IV are always co-localized but there are a few subtle discrepancies of the local synteny in A8-*Bb* versus *Tk*–*Te*. The former pair has a hypothetical gene inserted between the B and A subunits, whereas in *Tk* and *Te* the two genes are adjacent. Again, the synteny of genes upstream of subunit B is conserved in all the four genomes but for those downstream of subunit A the scenario in A8-*Bb* is different from that in *Tk*–*Te*.

As such, the close affinity between the A8 and *Bb*, or *Tk* and *Te* genomes is best reflected in the shared syntenies of their gene clusters for T4SS (Figure A in File S1), tight adherence (Tad) transport system (Figure B in File S1) and DnaK heat shock chaperone (Figure C in File S1).

### Functional Overview of the Four Genomes: Gene Allocation to Different Metabolic Categories

**Figure 4:**
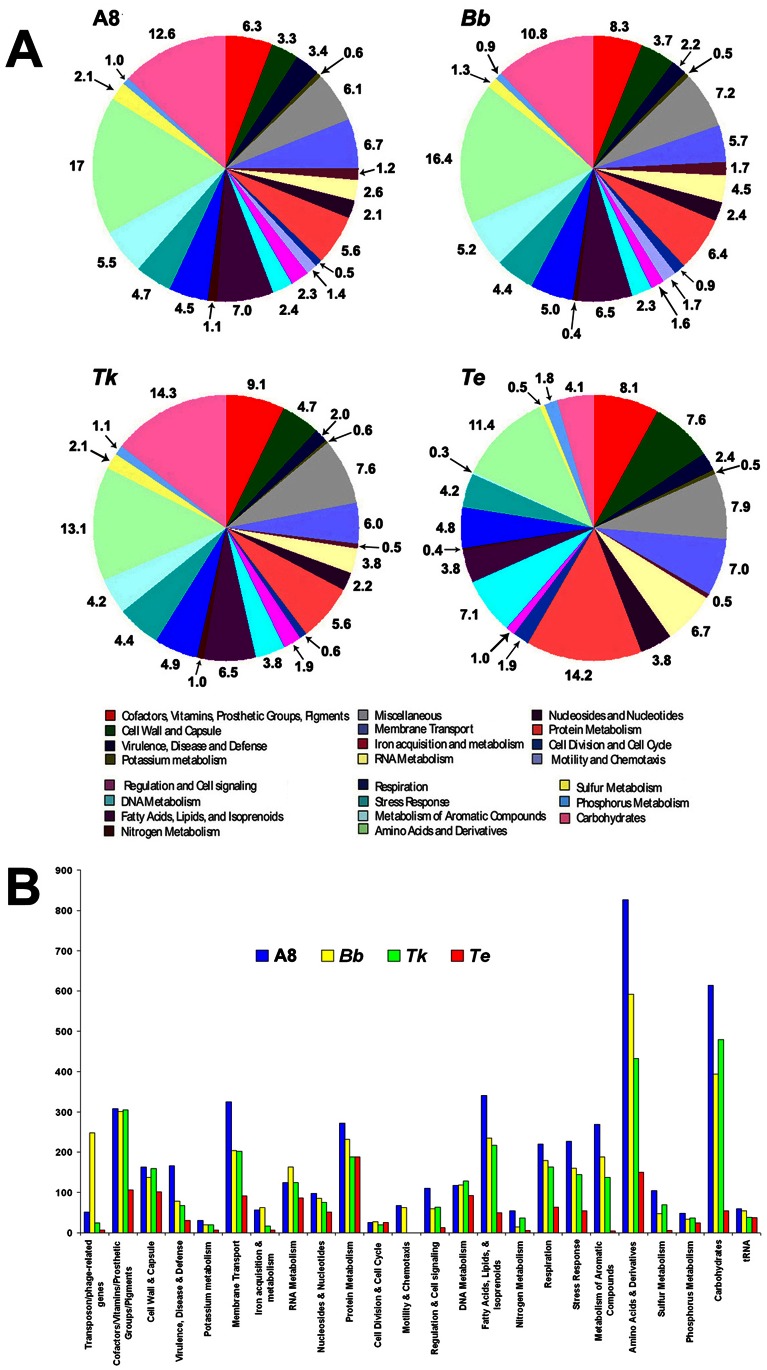
Functional classification of the gene content of the studied genomes. All predicted PEGs are classified and colored according to the different categories of the cluster of orthologous groups (COG) database. Proteins matching COG database entries not assigned to a particular COG category are classified as ‘Miscellaneous’ category. 25%, 28%, 30% and 15% of all the genes of A8, *Bb*, *Tk* and *Te* respectively could not be classified into any COG category and were not included in these diagrams. **A8**, *A. xylosoxidans* A8; ***Bb***, *B. bronchiseptica* RB50; ***Tk***, *T*. *kashmirensis* WT001^T^; ***Te***, *T. equigenitalis* MCE9. (**A**) Classification is represented in the form of percentage of total PEG content. (**B**) Classification is represented in the form of actual number of PEGs ascribable to each functional category.

In order to compare the gene contents of these bacteria and understand their metabolic and adaptive strategies we first used the cluster of orthologous groups (COG) database located at http://www.ncbi.nlm.nih.gov/COG/to classify all predicted gene models according to the COG to which they belonged. Subsequent to this we compared the distribution of the COG categories over the four genomes (Figure 4). Again, when genes for all the functional steps necessary to give rise to a complete pathway variant were present in a genome, they were regarded as constituting a metabolic subsystem or pathway variant [20], whereas the other predicted PEGs that did not conform to this criterion were considered not to be in subsystem. In this way functional classification followed by holistic comparison of the individual gene contents helped identify the common capabilities of the four *Alcaligenaceae* in conjunction with their species-specific aptitudes. While genes shared by all the species in question offered insight into the survival and growth strategies of the putative LCA, loci unique to any one, or some of them, explained the origins of various adaptive divergences.

**Figure 5:**
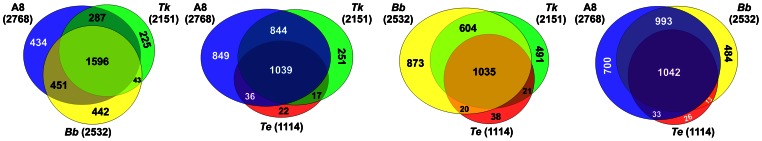
Tripartite comparison of the gene contents of the four *Alcaligenaceae* in question. The Venn diagrams show the number of shared as well as species-specific genes among three genomes at a time. These calculations include only those genes of a given genome which constitute complete pathway variants or metabolic subsystems (when genes for all the functional steps necessary to give rise to a complete pathway variant are present in a genome they are said to be constitute a metabolic subsystem). Numbers in parentheses indicate the total number of genes in metabolic subsystems in the organism in question. **A8**, *A. xylosoxidans* A8; ***Bb***, *B. bronchiseptica* RB50; ***Tk***, *T*. *kashmirensis* WT001^T^; ***Te***, *T. equigenitalis* MCE9.

Although the four Alcaligenaceae are taxonomically separated at the genus level and have discrete eco-physiological adaptations, they share remarkably high number of orthologs (PEGs having ≥60% identity). This observation points towards a high degree of functional conservation in the entire family. Notably, >50% of the PEGs that are in subsystems in any of the four genomes were found to be shared by at least one of the other compared genomes (Figure 5).

A clear proportionality exists between the relative size of the studied genomes and their respective number of predicted gene models (Table 1). This proportionality, however, does not hold when the number of complete pathway variants (or metabolic subsystems) encoded by the respective genomes is considered. In other words, the reduced genome sizes of *Bb* and *Tk* with respect to A8, or for that matter *Te* with respect to the other three, are not complemented by proportionate cutbacks in the number of pathway variants predicted for them (Table 1). Greater gene contents not effectively translating into added metabolic aptitudes imply that the genomes of A8 and *Bb* have, over evolution, been significantly inflated by multiplication of functionally similar CDSs via large scale events of gene paralogy.

Current analyses showed that A8 and *Bb* have dedicated almost equal percentages of their respective genetic inventories towards their 23 different metabolic categories (Figure 4A). However, a close numerical comparison revealed that *Bb* has undergone significant reduction in gene counts pertaining to stress response, membrane transport, amino acids utilization, carbohydrate utilization, nitrogen metabolism, sulfur metabolism and even virulence and defense. It is mostly with respect to phage-related genes, and partly iron-acquisition and RNA metabolism, that Bb has got more enriched than A8 (Figure 4B). The pattern in which *Tk* distributes its coding resources over the 22 metabolic categories is essentially similar to that of A8 and *Bb*. Notably however, the former completely lacks the flagellar motility and chemotaxis gene loci (Figure 4A), which in A8 and *Bb* are organized in syntenic clusters located in comparable positions in the 360 degree genomic context. Interestingly again, *Tk*’s gene count for carbohydrate utilization, nitrogen, sulfur, and phosphorus metabolism is only next to those of A8, even as genes for stress responses and amino acids utilization are less than the number found in A8 as well as *Bb* (Figure 4B).

Among the four species, *Te* alone has a completely distinctive plan for genomic resource allocation (Figure 4A). It dedicates relatively smaller fractions of its total gene content towards the metabolism of carbohydrates, sulfur compounds, aromatic compounds and fatty acids, lipids and isoprenoids, and at the same time devotes disproportionately more genes for cell wall and capsule; membrane transport; and metabolism of nucleotides, nucleosides, DNA, proteins and phosphorus. Interestingly, when we look at how *Bb*, the other pathogen in question, has spread its genetic resources over these very metabolic categories, we find closer strategic resemblance with A8 (or even *Tk*), rather than *Te*. This discrepancy is most pronounced in case of categories like metabolism of aromatic compounds, fatty acids, lipids, and isoprenoids and phosphorus, where *Bb*, A8 and *Tk* have a far higher percentage of gene allocation than *Te* (Figure 4A). So far as metabolizing carbohydrates or sulfur compounds are concerned, *Bb* has substantially less provisions than the two environmental *Alcaligenaceae*, but definitely not as low as *Te*. Again, for the metabolism of nucleotides and nucleosides *Te* has allocated a much higher percentage of its genetic repertoire than the equivalently low fractions devoted by A8, *Bb* and *Tk*. A similar scenario is observed in the case of phosphorus metabolism, where *Te* has devoted almost double percentage of genes than A8, *Tk* or *Bb*. Partitioning of more genomic resources to membrane transport and development of cell wall and capsule by *Te* is justified by its host-adapted existence. But why *Tk*, out of its relatively smaller genetic inventory, dedicates equivalent (or even higher, as in the case of cell wall and capsule) proportions (Figure 4A) as well as actual numbers (Figure 4B) of genes for these purposes than *Bb* is difficult to explain solely on the basis of their adaptive relevance. So far as membrane transport is concerned, it is again equally intriguing to note that A8, despite being a free living soil bacterium, has also dedicated much higher proportion (Figure 4A) as well as actual number (Figure 4B) of genes than *Bb*.

Other strategic anomalies notwithstanding, characteristics like fewer genes for nitrogen metabolism, absence of the sulfur oxidation (*sox*) locus, and relatively higher allocations for RNA metabolism and cell division and cell cycle do unite the two pathogens in opposition to the two environmental isolates. Again, *Te*, corroborating its host-adaptation, devotes an exceptionally high percentage of genes (Figure 4) for the metabolism of proteins. *Bb* expectedly devotes the next highest percentage towards protein metabolism.

### Functional Overview of the Four Genomes: Shared Genetic Features

Structural and functional faculties for which all essential genetic complements are present in *Bb* and A8, but not in *Tk* and *Te*, include flagellar motility and chemotaxis; glutathione utilization (as sulfur source); tetrathionate reduction/respiration; trehalose biosynthesis, maltose and maltodextrin utilization (some genes for this are present in *Tk* WT001^T^, which is phenotypically maltose –ve; *Tk* strain WGT is however maltose +ve), and glycogen metabolism; D-alanyl lipoteichoic acid (Gram +ve cell wall component) biosynthesis; quorum sensing; central meta-cleavage pathway of aromatics degradation; and triacylglycerol metabolism. Complete absence of loci for chemotactic response and flagellar structure and function notwithstanding, 17 response regulators consisting of CheY-like receiver domains, at least eleven signal transduction histidine kinases (associated with different metabolic loci), and five more two-component sensor kinases/response regulators (out of which four have been pseudogenized) could be detected in the *Tk* genome. In contrast, A8 and *Bb* were found to possess a minimum of 51 and 35 two-component sensor kinases/response regulators respectively. The genome of *Te* in its turn encompasses two histidine kinases and eight two-component sensor kinases/response regulators. Limited environmental-sensing capabilities and very few regulators of gene expression are normal characteristics of a critically host-adapted pathogen like *Te* that is restricted to a defined resource base and stable set of environmental conditions. But how a free-living facultative autotroph like *Tk* responds and adapts to diverse external stimuli with such meager sensory resources is a riddle worth investigating. Although its large repertoire of transcriptional regulators (Table 2) may be significantly useful in this direction, the issue still remains perplexing in view of the fact that the Himalayan apple orchard soil (in an area that enjoys long frozen winters and brief hot-humid summers), from where *Tk* was isolated, experiences remarkable seasonal fluctuations in its physico-chemical characteristics like temperature, humidity, nutrient availability etc.

**Table 2:** Number of major regulatory gene types present in Tetrathiobacter kashmirensis WT001^T^ (Tk), Achromobacter xylosoxidans A8, Bordetella bronchiseptica RB50 (Bb) and Taylorella equigenitalis MCE9 (Te).

PEG identified	A8	*Bb*	*Tk*	*Te*
Transcriptional regulators belonging tothe LysR family	202	128	96	2
Transcriptional regulators belonging tothe TetR family	32	24	23	1
Transcriptional regulators belonging tothe MarR family	21	10	16	1
Transcriptional regulators belonging tothe MerR family	06	06	04	01
Transcriptional regulators belonging tothe GntR family	72	52	41	0
RNA polymerase sigma factors	34	15	12	2

*Bb*, in its turn, shares a handful of such unique genes with *Tk*, which are partially or completely missing in A8 or *Te*. These include capsular polysaccharides biosynthesis, export and assembly (many of these genes are however present in A8 and *Te*); pyruvate-alanine-serine interconversion; urea decomposition (several urea transporters are, anyway, present in A8) and transport of nickel and cobalt. Partial conservation of attributes like urea decomposition (conversion of urea to ammonia and carbon dioxide) points towards ancient acid adaptation of *Alcaligenaceae*. Urease converts urea to ammonia and carbon dioxide, and the former buffers acidic environments by increasing their pH [13]. Nickel has been proved essential for the formation of the catalytic centre of the urease complex of *Helicobacter* species [13]. Notably, both *Tk* and *Bb* have a HupE/UreJ family metal transporter (putatively involved in the uptake and transport of nickel) and a Ni^2+^-binding GTPase (UreG, putatively involved in regulation of expression and maturation of ureases and hydrogenases) nested within their urease gene clusters (two in case of *Tk* and one for *Bb*). This indicates that the urease of both these organisms could be similar metalloproteins having bound nickel ions.

Insinuation of ancient acid adaptation in *Alcaligenaceae* is also apparent from the occurrence of putative tetrathionate reduction/respiration systems in the genomes of *Achromobacter* and *Bordetella* spp., in conjunction with proven tetrathionate oxidation in *Tk* [6]. Tetrathionate, in its stable form, is infrequent in the environment, and occurs only in some typical acidic habitats. As such, across the board use of this rare compound for various redox purposes could well have an ancient background. Corroborating this hypothesis, large numbers of organic acid metabolizing systems – e.g., pathways for tricarballylate utilization, methylcitrate cycle, glycerate metabolism, propionate-CoA to succinate conversion lactate utilization, etc. - are encoded by A8, *Bb* and *Tk*, but not *Te*. In this category, the *Te* genome encodes only an L-lactate dehydrogenase and a *tcuAB* homolog that putatively oxidize tricarballylate to cis-aconitate. Corroborating these attributes, various tripartite ATP-independent periplasmic (TRAP) solute transporters, specific for the uptake of organic acids, were found to be conserved in the *Alcaligenaceae*.

Shared gene clusters of the two soil dwellers, A8 and *Tk*, which are absent in the two pathogens include loci governing xylose utilization, arsenic resistance, benzoate degradation, Entner-Doudoroff pathway, zinc-regulated enzymes, molybdenum cofactor biosynthesis, phospholipid & fatty acid biosynthesis, alkylphosphonate utilization and inorganic sulfur oxidation.

*Tk* and *Te*, in their turn, share only a few unique genes like the co-transcribed *umuD* (encoding the error-prone DNA repair protein UmuD) and *umuC* (encoding error-prone lesion bypass DNA polymerase V) homologs. In case of *Te*, *umuDC* is located in the chromosome adjacent to one of the overall two DNA helicase IV genes possessed by this organism. But in *Tk*, the pair is plasmid borne and not associated with any ATP-dependent DNA helicase *uvrD*/*pcrA*. There are, nevertheless, three *uvrD*/*pcrA* homologs in the *Tk* chromosome, out of which one is a pseudogene. In this context it is worth mentioning that a detailed comparison of the DNA metabolizing machineries of A8, *Bb*, *Tk* and *Te* (described in File S2) revealed various degrees of shortcomings in their gene contents for replication, recombination and repair. Relevant data suggested that all the four genomes could, more or less, be intrinsically prone to the incorporation of global mutations at abnormally high rates, but high mutability and reduced ability to accommodate foreign DNA via homologous recombination could be much more acute in case of *Tk* and *Te*. Elimination of several DNA repair genes may have put a bias mutational pressure upon the *Te* genome that in its turn has probably led to the unusual increase in its A+T content. It is tempting to conjecture that a similar fate awaits the *Tk* genome because it has not only got a large number of its repair and recombination genes pseudogenized, but has also assumed one of the highest A+T contents among the free-living *Alcaligenaceae*. Evolutionary implications of these degenerative genomic trends have been discussed in the subsequent sections.

### Functional Overview of The Four Genomes: Unique Genetic Features

Unique aptitudes encoded by the *Tk* genome include carbon fixation (Calvin-Benson cycle, CO_2_ uptake, photorespiration/oxidative C_2_ cycle, pyrroloquinoline quinone biosynthesis) and utilization of certain simple carbohydrates like acetone, malonate, L-fucose, D-galactarate, D-glucarate, D-glycerate, D-gluconate and ketogluconates. In addition, there are a few more exclusive genetic factors that could have been instrumental in getting *Tk* selected in its apple orchard soil habitat rich in typical carbon and nitrogen sources. Genes for malonate transport (MadL and MadM subunit) and utilization (malonate decarboxylase alpha, beta, delta, and gamma subunits, malonate utilization transcriptional regulator, malonyl CoA acyl carrier protein transacylase, phosphoribosyl-diphospho-CoA transferase and triphosphoribosyl-diphospho-CoA synthetase) are striking examples in this regard. In addition, genes encoding (i) acetone carboxylase (the key enzyme of acetone metabolism which enables a bacterium to grow using acetone as the primary source of carbon and energy), (ii) quinoproteins (that enable growth with various alcohols as the sole source of carbon and energy), (iii) pyrroloquinoline quinone (PQQ, typical of methylotrophic bacteria, which have selective advantage in aerobic phosphate-limiting environments) biosynthesis, (iv) PQQ dependent glucose dehydrogenase, methanol dehydrogenase, and quino (hemo)protein alcohol dehydrogenase, (v) periplasmic nitrate reductases like ferredoxin-type protein NapG and polyferredoxin NapH (which catalyze electron transport from the membrane-seated quinol pool to the periplasmic nitrate reductase), and (vi) the periplasmic aromatic aldehyde oxidoreductase (involved in purine utilization) could also confer crucial selective advantage to *Tk*. Again, genes governing the catechol and protocatechuate branches of the beta-ketoadipate pathway (more specifically mandelate racemase and protocatechuate 3,4,-dioxygenase alpha and beta chain, and salicylate esterase and salicylate hydroxylase for salicylate and gentisare degradation) could provide further adaptive edge to this organism in its pesticide-laden soil habitat.

On the other hand, only few complete genetic systems such as those for lactate fermentation, mixed acid fermentation and biphenyl degradation could be identified in A8 which were totally missing in the other three including *Bb*.

Similarly, a close scrutiny of the *Bb* genome reveals only a handful of such exclusive metabolic systems that are missing in A8, *Tk* and *Te*. These include pertussis toxin production, type III and type V protein secretion systems (T3SS and T5SS) and phosphonoalanine utilization (notably, T5SS autotransporters are there in A8 and *Tk*, albeit in numbers far less than *Bb*). Pertussis toxin and the two secretion systems clearly confer on *Bb* a parasitic edge over its phylogenomic relatives [21], [22], but with the exception of these attributes, the *Bb* genome is pretty much a subset of A8, with all the basic structural and functional genes needed for host-adaptation being already there in A8, and more interestingly sometimes in *Tk* also. Accordingly, it is no wonder that *Ax* strains, like *Bordetella* species, are often infectious [23] and at times even get misidentified as bordetellae [24].

Notably, there are only a few such unique genes in *Te* which do not have homologs in any of the compared *Alcaligenaceae* (Table E in File S1). None of these genes appear to be decisive for the survival of *Te* in its equine genitalia, even though some of them may add to its adaptive fitness. Except for these few unique elements, the *Te* genome is essentially a small subset of the consensus *Alcaligenaceae* genome, and more specifically that of *Tk*. Most significantly, orthologs of all the *Te* genes putatively involved in host cell binding and colonization [25] were found to be present in the genome of *Tk*, or for that matter A8 and *Bb*. These included genes for (a) O-antigens, (b) proteins containing eukaryotic ankyrin or tetratricopeptide repeat motifs, (c) hemagglutinin-related proteins, (d) RND efflux systems, (e) four secretion systems including T4SS, (f) YadA and Hep_Hag domains containing proteins, (f) TonB-dependent receptors, and (g) the chaperonin GroEL (HSP60 family). Again, no proven or putative cytotoxic or cytolytic factor is noticeable when one looks at the few features that *Te* exclusively shares with the other pathogen *Bb* (Table E in File S1). So it seems quite reasonable to infer that basic calibers for attaching, colonizing and persisting in host tissues could have had an early origin in *Alcaligenaceae*. Logically, this means facultative asymptomatic commensalism or opportunistic pathogenicity as practised by species of *Taylorella* [25], [26] or *Achromobacter* [5], [27] may also be intrinsic to *Tk*.

### Ubiquity of Virulence Factors in Pathogenic as well as Non-pathogenic *Alcaligenaceae*

The *Alcaligenaceae* in question (together with several other pathogenic as well as non-pathogenic members of the family) were found to share a large number of genetic systems that have been experimentally or theoretically earmarked as virulence factors in pathogenic bacteria including *Bordetella* [28], [29] and/or *Taylorella* [25]. These genomic features included secretion mechanisms such as the Tad macromolecular transport system that assembles adhesive Flp (fimbrial low-molecular-weight protein) pili in diverse pathogenic bacteria, Type 4, Type 6 and Type 7 (Chaperone-Usher) secretion systems, plus several complements of T5SS autotransporters (see details in File S3); various protein degradation mechanisms (Table F in File S1); and diverse resources for the development of lipopolysaccharide envelops and surface antigens (File S4). Iron uptake mechanisms (including those contrived for obtaining iron from iron-complexed host proteins), which are central to the virulence of *Bordetella* and several other pathogenic bacteria living in perpetually iron-impoverished environments [13], are also very well developed across *Alcaligenaceae* (see details in File S5). These findings collectively provoke the conjecture that this betaproteobacterial family as a whole could be a storehouse of potent host-infecting aptitudes, which may be summoned by the relevant possessors under situations of compulsion or opportunity. It is also not unlikely that some rudimentary set of virulence factors had already been there in the LCA of these closely related *Alcaligenaceae*, and subsequent to their ecological radiation that ancestral repertoire of genes got elaborated or economized depending on the adaptive requirement (or the lack of it) of the different members. At this point of understanding it is worth remembering that the so called virulence factors like secretion systems, lipopolysaccharides etc. are often used by bacteria in non-pathogenic contexts to render different physiological functions in response to various environmental challenges. As such, it would be more prudent to refer to *Tk* or A8 homologs of the so-called virulence-related genes as potential host-interaction factors unless their precise roles in these environmental *Alcaligenaceae* are experimentally elucidated by transcriptomic and proteomic investigations. Such studies should be aimed at identifying the plausible conditions that can trigger the expression of these genes, besides which it would also be imperative to know the actual substrates of the protein products of these genes.

### Identification of Potent Virulence Factors in the *Tk* Genome

Identification of potential virulence factors in the *Tk* genome was especially interesting since, until now, this free-living facultative chemoautotroph had no direct or indirect inkling of host-interaction, whatsoever. As such, future in-depth scrutiny of its haemolysins, autotransporter adhesins, surface antigens and protein secretion systems would be of immense significance in our understanding of the emergence of novel pathogens. Over and above those host-interaction factors which could be used in pathogenic as well as non-pathogenic contexts, the following genomic features of *Tk* specifically drew our attention with reference to its potential as a future pathogen:

- A five-gene locus (nt. position 3686491 to 3697360) encoding one hemagglutinin/hemolysin-like pseudogene; three filamentous hemagglutinin family outer membrane exoproteins putatively involved in heme utilization or adhesion and one hemolysin activation/secretion protein. Interestingly, putative products of these genes showed maximum sequence identities (in the range of 35–50%) with homologs from animal pathogens like *Neisseria meningitides* and *Klebsiella* spp.; plant pathogens like Banana blood disease bacterium R229, *Ralstonia* spp. [30] and *Xylella fastidiosa* [31]; and even rhizosphere-colonizing and plant growth-promoting bacteria like *Pseudomonas fluorescens* F113, which biocontrols fungal plant pathogens [32]. In contrast, homology with similar gene products from other *Alcaligenaceae* was always a few percentages lower than these. Percentage G+C content of this locus (57%) was more or less same as that of the rest of the *Tk* genome. These facts indicate a long residence time of these genes in various *Alcaligenaceae* genomes and differential accumulation of mutations therein.
- Two hemolysin genes ascribed to COG3176 and COG3042 respectively and encoding putative products that belong to two different groups of hemolysins. Out of the two, the former exhibited maximum sequence identity (∼56%) with homologs from A8, *Bb*, *Bp*, *Bpp* or *Te*, while the other showed highest (∼54%) identity with several DUF333 domain-containing homologs from *Comamonadaceae* of *Burkholderiales*. Notably, the second gene does not have any homolog in *Alcaligenaceae*, except *Alcaligenes faecalis* (45% identity) and *Bordetella avium* (38% identity).
- One MviN-like transmembrane protein having maximum (∼53%) identity with homologs from diverse *Bordetella* and *Achromobacter* species, plus a host of other betaproteobacteria. However, the related homolog from *Te* as well as the proven virulence factor MviN of *Salmonella enterica* subsp. enterica serovar Typhimurium [33] had 44% identity with the *Tk* gene. MviN homologs are widespread in bacteria as diverse as pathogens, non-pathogens and plant-symbionts [34], and are often associated with flagellation and motility [33], [35].
- The plasmid pWTk445, in its turn, encompassed a putative outer membrane protein A having OmpA/MotB and SmpA/OmlA domains (encoded by TKWG_25829) with maximum (45–50%) identity with homologs from *Neisseria* spp., followed by several other pathogenic bacteria. Presence of another ortholog in the *Tk* chromosome (TKWG_04980 having 44% identity) notwithstanding, this kind of a double domain outer membrane PEG is not present in any other sequenced *Alcaligenaceae* except *Pusillimonas* sp. T7-7. Notably however, there is a third comparable PEG (TKWG_17880) in the *Tk* chromosome, which has only the SmpA/OmlA domain but is highly conserved in pathogenic as well as non-pathogenic *Alcaligenaceae* (>60% mutual identities).
- The plasmid also has an autotransporter adhesin (TKWG_25524) located next to *ompA* and having a YadA adhesin-like C-terminal domain that is typical of “essentially virulence-related” type Vc secretion systems [22]. This putative gene product also encompasses a HIM motif that is often found associated with YadA domains in diverse invasins and haemagglutinins. The translated amino acid sequence of TKWG_25524 showed maximum (∼50%) identity with several homologs from *Neisseriales* and *Pasteurellales*, besides only A8, *Te* and *Pusillimonas* sp. T7-7 among the *Alcaligenaceae*. There is also one chromosomal counterpart (TKWG_16370) of this autotransporter adhesin having a maximum of 30% identity with several YadA-like homologs from *Yersinia* spp. and only 25% identity with TKWG_25524.
- Another pWTk445 gene worth mentioning in this connection is TKWG_25809 which encodes a hemolysin III superfamily membrane protein having highest (∼45%) identities with homologs from *Pseudomonas* spp. Homologs of this putative PEG have been reported as having cytolytic activities elsewhere [36].

### Hemolytic Activity of *Tk*

**Figure 6:**
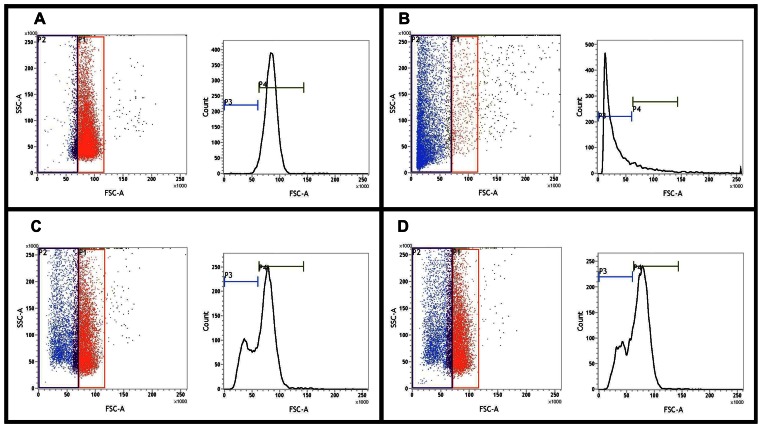
Hemolytic activity of *T kashmirensis* WT001^T^ (*Tk*) measured by flow cytometry. In all the four documents left panels represent the dot plots of the relevant flow cytometry analysis, while right panels show corresponding histograms depicting the medians of the FSC/SSC ratios. The regions of interest for all these plots were determined by first gating intact uninfected hRBCs (no lysis) in the red bordered area of **A**, which interned ∼87% of all hRBCs. **B** shows the level of hRBC lysis achieved by treatment with water. Copious shift of the hRBCs towards the left of the main red gate is noticeable with ∼83% of all hRBCs figuring in the blue bordered area on the left of the main red gate. In all subsequent calculations this level was taken as the maximum possible level of lysis or positive control. **C** and **D** respectively shows the level of hRBC lysis achieved (after four hours of infection) by *Tk* cells grown in iron-depleted and iron-repleted media at hRBC:bacterial cell ratio of 1∶100. In **C** and **D** respectively, ∼44% and ∼43% of the total hRBCs were found to occur in the blue bordered area on the left of the main red gate.

Since several hemolysin genes were identified in its genome we deemed it imperative to test the hemolytic potential of *Tk*. Significant hemolytic activity of *Tk* cells was observed against human red blood cells (hRBC). Remarkably, this activity was not affected by the availability of iron in the bacterial growth medium. A maximum of 53% hemolytic activity [relative to water-lysed hRBCs taken as the maximum possible level of lysis or positive control (Figure 6B)] was observed for iron-starved *Tk* cultures at the hRBC:bacterial cell ratio of 1∶100 (Figure 6C). Again, maximum hemolytic activity of *Tk* cells cultured under iron-repleted conditions was ∼52% (relative to the positive control), that too at 1∶100 hRBC:bacteria ratio (Figure 6D). In experiments with *Tk* cells grown in both iron repleted and iron-depleted conditions increase in hemolytic activity was observed proportionate to the increase in the multiplicity of infection (MOI) up to the hRBC:bacterial cell ratio of 1∶100. No more increase in hemolytic activity was observed beyond this MOI level (data not shown).

### Ability of *Tk* to Adhere to Eukaryotic Cells

**Figure 7:**
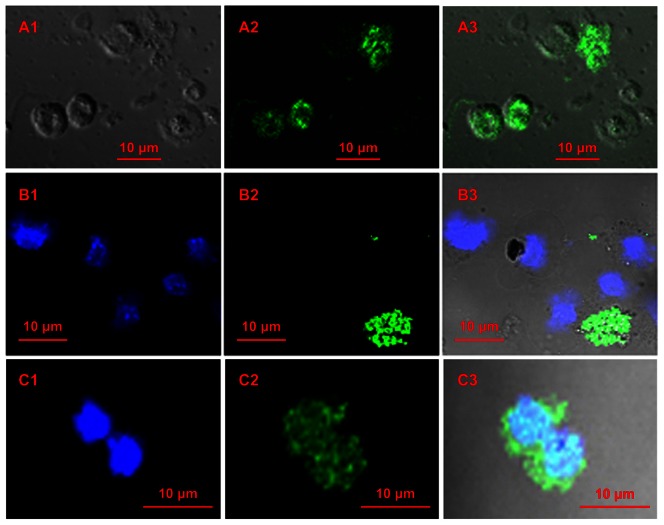
Laser-scanning confocal micrographs showing adhesion of *T kashmirensis* WT001^T^ (*Tk*) to eukaryotic cell lines. (**A**) *Tk*’s adhesion to HeLa cells. A1, DIC image showing HeLa cells; A2, image generated upon exciting only FITC shows the positions of the bacterial cells in the same field; A3, superimposition of A2 upon A1. (**B**) Negative control of the adhesion test involving the environmental betaproteobacterium *Herminiimonas arsenicoxydans* LMG 22961^T^ and HeLa cells. B1, image generated upon exciting only DAPI shows the positions of the HeLa cells; B2, image of clumped bacterial cells from the same field generated upon exciting only FITC; B3, superimposition of B2 upon B1. (**C**) *Tk*’s adhesion to macrophage RAW264.7**.** C1, image generated upon exciting only DAPI shows the position of the macrophages; C2, bacterial image from the same field generated upon exciting only FITC; C3, superimposition of C2 upon C1.

In view of a number of genomic indications we also tested the potential of *Tk* cells to adhere to different eukaryotic cell lines by laser-scanning confocal microscopy. HeLa (Figure 7A) and Macrophage RAW264.7 (Figure 7C) cell lines were tested for this purpose. In either case adherence of virtually 100% bacterial cells was apparent within one hour of infection at all the tested MOIs (eukaryotic cell:bacterial cell ratios 1∶1 to 1∶20). In all the surveyed microscopic fields, little or practically no *Tk*-specific FITC (fluorescein isothiocyanate) signal was detected except from the surface of the eukaryotic cells, which in their turn were identified by DAPI (4',6-diamidino-2-phenylindole) as well as DIC (differential interference contrast) imaging. Notably however, other environmental betaproteobacteria such as *Herminiimonas arsenicoxydans* LMG 22961^T^ showed no binding with eukaryotic cells (Figure 7B). Although we have not yet extended these cytological studies with *Tk* any further, it would surely be imperative to perform more advanced experiments to check whether *Tk* can modify the structure and function of macrophages, or whether it has potent invasive cytotoxic or cytolytic properties or not.

### Retracing *Alcaligenaceae* Evolution

**Figure 8:**
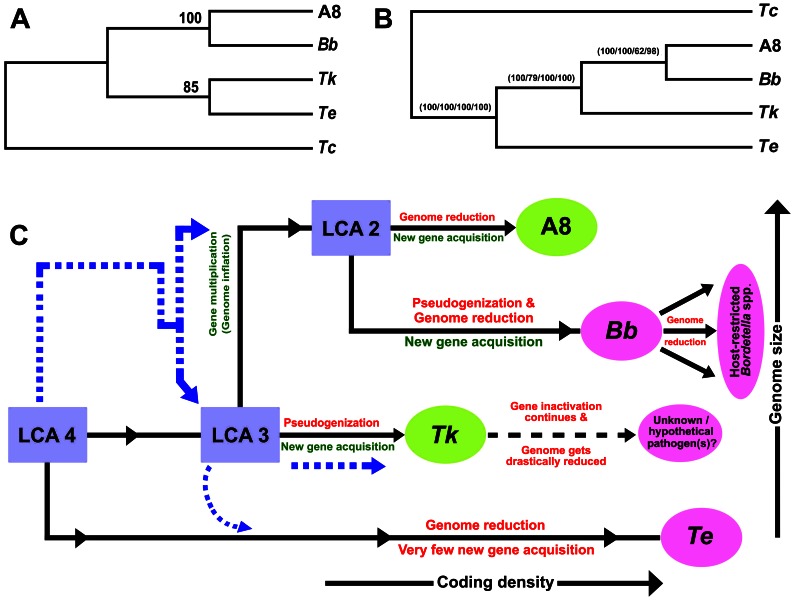
Plausible evolutionary path of the *Alcaligenaceae* in question. **A8**, *A. xylosoxidans* A8; ***Bb***, *B. bronchiseptica* RB50; ***Tk***, *T*. *kashmirensis* WT001^T^; ***Te***, *T. equigenitalis* MCE9. (**A**) Majority rule consensus tree based on 16S rRNA gene sequences. Same tree topologies were obtained by applying distance matrix, maximum likelihood as well as parsimony-based methods. Bootstrap values (100 replicates) are given only for the parsimony analysis. The gammaproteobacterium *Thiomicrospira crunogena* XCL-2 (*Tc*) was used as outgroup in all the analyses. (**B**) Common topology of majority rule consensus trees constructed on the basis of 23S rRNA, *recA*, *dnaA* or *dnaK* gene sequences. Similar tree topologies were obtained by applying distance matrix, maximum likelihood as well as parsimony-based methods. Bootstrap values (100 replicates) are given for the parsimony analyses with 23S rRNA (first value), *recA* (second value), *dnaA* (third value) and *dnaK* (fourth value) gene sequences. *Tc* was used as outgroup in all the analyses. (**C**) Two alternative evolutionary paths suggested by comparative genomic analyses are shown in solid black and dashed blue lines. Dashed black lines represent an uncharted area of evolution. LCA: last common ancestor; LCA4: LCA of all the four species in question, viz., A8, *Bb*, *Tk* and *Te*; LCA3: LCA of the three organisms A8, *Bb* and *Tk* which may or may not have been the stock from where *Te* diverged; LCA2: LCA of the two organisms A8 and *Bb*.

After collating the whole gamut of genomic attributes and relationships it was unambiguous that in the immediate evolutionary past A8 and *Bb* shared a common ancestor, which was distinct from the other contemporary stock that gave rise to *Tk*, and perhaps also *Te*. Branching of *Taylorella* in the phylogeny of *Alcaligenaceae* is uncertain. Past [37], [38], [39] as well as present (Figure 8A) 16S rRNA gene sequence-based analyses clustered *Ax* and *Bb*, and *Tk* and *Te* in two distinct monophyletic branches and supported a clear dichotomy between the two lineages. On the other hand, tree topologies derived from 23S rRNA, *recA*, *dnaA* or *dnaK* gene sequence relatedness suggested an early divergence of *Te*, followed by that of *Tk* and finally the A8-Bb cluster (Figure 8B).

Comparative genomic logics also appeared to be inept in deciding whether *Te* diverged early from the LCA of all the four *Alcaligenaceae* (denoted as LCA4 in Figure 8C) or shared an immediate common ancestor (ICA) with *Tk* (i.e., diverged alongside *Tk* from the LCA3 of Figure 8C tracing the dotted blue lines). As such, there was no means to ascertain whether the genetic subsystems which are totally absent in *Tk* and *Te* but are present in A8 and *Bb* (i) were acquired independently by the LCA2 (this scenario is independent of whether evolution followed the solid black or the dotted blue lines in Figure 8C), or (ii) were present in LCA 4 but got removed at the level of LCA3 (under this scenario evolution is bound to have occurred along the dotted blue lines of Figure 8C since it is very unlikely that such selective cleansing events independently took place twice in sub-populations of LCA4 as well as LCA3). Similarly, unique genes shared by *Tk* and *Te* may be looked upon in two different ways: (i) as ancestral attributes lost during the divergence of LCA2 (this scenario too is independent of whether evolution followed the solid black or the dotted blue lines of Figure 8C) or (ii) as genes not present in LCA4 but acquired discretely by LCA3 (under this scenario evolution is bound to trace the dotted blue path of Figure 8C). Although no genomic data could ascertain whether *Te* diverged from LCA4 or LCA3, detection of at least 36 such *Te* genes which are missing in *Tk* but present in A8 and/or *Bb* (Table E in File S1) confirmed that the reductive divergence of *Te* did not happen via *Tk*. Likewise, it was also certain that none of the four genomes in question was a direct derivative of any of the other three. This inference is buttressed by the fact that all the possible genome pairs (viz., A8-*Bb*, A8-*Tk*, A8-*Te*, *Bb*-*Tk*, *Bb*-*Te*, *Tk*–*Te*) shared at least a few such exclusive genes or genetic systems that were absent in the other two.

**Figure 9:**
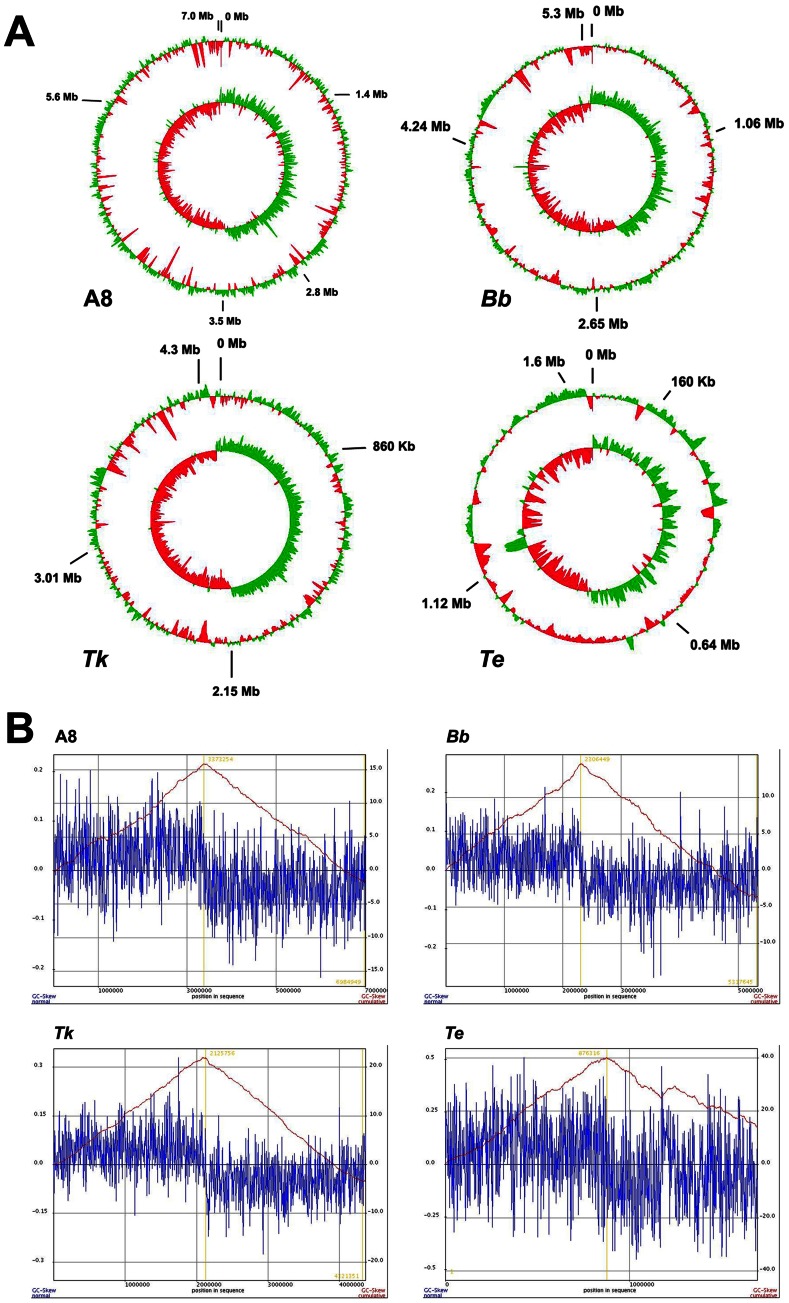
GC-skew in the studied *Alcaligenaceae* genomes. **A8**, *A. xylosoxidans* A8; ***Bb***, *B. bronchiseptica* RB50; ***Tk***, *T*. *kashmirensis* WT001^T^; ***Te***, *T. equigenitalis* MCE9. In order to get correctly comparable pictures the GenBank-retrieved genome sequences of *Bb* and *Te* were reorganized before these analyses so as to make *dnaA* the first gene. (**A**) Circular maps of the four genomes. Outer circles indicate deviations from average percentage G+C contents, while the inner circles denote GC skews which is equal to (G-C)/(G+C). Percentage G+C contents as well as GC skews were calculated using sliding windows of 10,000 bp with a window step of 100. (**B**) Cumulative GC-skews of the four genomes showing minima and maxima at the origin and the terminus of replication respectively.

Preponderance of intra-genomic paralogy in A8 and *Bb*, but not in *Tk* or *Te*, insinuate that LCA4 (or for that matter, LCA3 also) must have had an intermediary genome size close to that of *Tk*. The essentially symmetric GC skew of the *Tk* genome (the leading strand being only ∼25 Kb shorter than the lagging strand) also supports its more or less conserved architecture over a long evolutionary time span (Figure 9). However, the exceptionally high number of pseudogenes, in combination with low coding area percentage of the genome (Table 1), does suggest *Tk* to have come out of a recent evolutionary bottleneck where genome degradation has just started. Accordingly, this genome seems to have plenty of scaffolds readied for future modification, which in turn would collectively lead towards speciation.

On the other hand, occurrence of significantly fewer pseudogenes in A8, *Bb* and *Te*, in combination with high coding densities, suggests that these evolutionarily matured genomes are unlikely to be degraded any further. However, the comprehensive evolutionary trend of *Alcaligenaceae* towards smaller genomes is suggested by the fact that the smallest of all the sequenced genomes (*Te*) has the highest coding frequency. Subtractive history of this genome is further evidenced by its GC skew asymmetry of ∼0.13 Mb, which includes a ∼72 Kb translocated segment of the leading strand (Figure 9).

The exclusive inflationary trends of the A8 and *Bb* genomes can be explained further by envisaging an evolutionary scenario where their ICA (i.e., LCA2 of Figure 8) evolved from LCA3 or LCA4 by expanding to more than 7 Mb via extensive paralogy and limited HGT. At that evolutionary juncture, *Tk* apparently managed to retain the core characteristics of the ancestors, even as it upgraded its metabolic aptitudes in tune with the demands of its environment. The more or less conserved architecture of the *Tk* genome suggests that these capacity additions via novel gene acquisition occurred mostly in exchange of such loci that were of no immediate adaptive advantage to the organism. Subsequent to the inflationary cycles at the level of LCA2, the genomes of A8 and *Bb* got differentially down-sized via selective deletions (of their paralogous genes in particular) in accordance with their environmental compulsions. Trimming of the two genomes, however, did occur in tandem with new capacity additions. Out of the two descendents, A8 underwent minimum loss or gain of genes in comparison to the LCA2. This is apparent from the more or less symmetric GC skew of its chromosome and possession of only few such complete gene loci that are all together missing in *Bb*, *Tk* and *Te*. It is however noteworthy that although the 7 Mb A8 genome is closer to the putative LCA2, it is not entirely identical to the latter. The ∼0.2 Mb shorter leading strand of the A8 chromosome does testify that the LCA2 had a genome that was at least somewhat bigger than A8. A huge asymmetry in the GC skew of the *Bb* genome (the leading strand being ∼0.7 Mb shorter than the lagging strand), on the other hand, definitely proves its evolution from a larger genome via large scale deletions (Figure 9). Presumably, this reductive trend died down in the RB50 lineage but persisted in other contemporary populations of the *Bb* ancestor, giving rise to further genome-minimized and host-restricted entities like *Bp*, *Bpp* and *Ba*. The ∼0.7 Mb genomic erasure in conjunction with the 1 Mb difference already attributed to gene multiplication accounts for almost the entire size difference between A8 and *Bb*. Corroborating this observation, exhaustive subsystem-wise comparisons (using RAST) revealed that the *Bb* genome was deficient in only a few genetic features (such as those rendering cytotoxicity) in comparison to A8. The unique metabolisms of A8, or for that matter *Tk*, are likely to be independent adaptive acquisitions, while such shared gene clusters of the two soil dwellers that are wanting in *Bb* could be viewed as ancestral traits disposed of by *Bb* owing to their irrelevance to its adaptation to mammalian hosts.

Although the common ancestry of the soil isolate A8 and the pathogen *Bb* was unambiguous it was not possible to ascertain whether genome economization in *Bordetella* spp. started after the chance introduction (seeding) of strains to particular host environments (presumably because metabolic processes became superfluous after host-adaptation) or whether host-confinements were direct consequences of self-degeneration of the genomes. This lacuna of understanding is also conspicuous in several other instances where genome reduction accompanies pathogen evolution [8], [12], [40]. Absence of such evolutionary links (extant species or strains) that could represent the bottleneck from where the pathogens in question purportedly came out is primarily responsible for this shortcoming. In most of the known cases of reductive evolution only the abridged genomes of the host-restricted pathogens and the unperturbed genomes of their free-living relatives are available for scrutiny, but here with regard to the origin of pathogenicity in *Taylorella* we were fortunate to get hold of that rarely-captured missing link (in the form of *Tk*) where degeneration of the genome (primarily in the form of sweeping pseudogenization) has already started but selective host-confinement has not yet set in. As such, auto-degradation of the genome of this soil-dwelling sulfur-chemolithoautotroph is currently occurring regardless of whether the organism can eventually mobilize itself to a suitable host refuge or not. This exceptional case prompted us to conclude that host-confinement was the inevitable destiny of a self-degenerating genome confronting meltdown or implosion.

### Genome Self-destruction Drives *Alcaligenaceae* Members Pathogenic

The convoluted overlaps of unique diversities and strategic commonalities observed among the *Alcaligenaceae* genomes explain two important aspects of their evolution. (1) They define those characteristic aptitudes (departures from the LCA) of species/strains/populations which make them best-fit variants to cope with specific environmental conditions. (2) They also show that diverged *Alcaligenaceae* genomes hold varying degrees of competence to acquire and/or evolve novel functions to usher their foray into uncharted ecological niches.

If one considers *Achromobacter*, the group has an extremely ramified taxonomic (infra-generic and infra-specific) structure and population dynamics [24], and consequentially a remarkably broad ecological niche width. Strains of *Achromobacter* spp., particularly those of *Ax*, are physiologically so versatile as to be able to opportunistically infect a wide variety of host tissues [5], [23], [41], [42] and at the same time live freely in natural habitats like fresh or marine waters, soils, etc [24]. Genomic data suggested that the extraordinary metabolic and adaptive plasticity of these bacteria (resulting in their wide ecological amplitude) stems from their huge genome content, and above all the advantage of having abundant alleles for a large majority of genes. Paralogous genes, per se, are prospective reservoirs of novel gene functions [43]. Because, whatever may be their source of origin in a genome, paralogs, over prolonged evolution, accumulate large number of mutations and eventually under appropriate selections emerge as the key to coping with new environmental challenges [44]. In this way they can also potentially compensate odd gene losses in the concerned genome.

Like achromobacters (and unlike other host-confined bordetellae), *Bb* possesses a copious genome content and multitude of alleles for a large majority of its genes. In addition, *Bb* still has sufficiently robust indigenous capacities for DNA metabolism, energy metabolism and ion transport, besides most of the other basic metabolic pathways and circuits of regulation and cell signaling that are typical of its environmental relatives. As in A8, signal transduction genes are also numerous in *Bb*, concurrent to which both the bacteria have several transcriptional regulators and sigma factors, which presumably act under different environmental conditions and help them occupy diverse ecological niches. All these attributes adequately explain why *Bb*, unlike its genome-downsized and host-obligated derivatives, is still capable of surviving freely in the environment. Its pathogenic aptitudes, like those of achromobacters, thus seem to be optional faculties and not obligated functions.

The ecophysiological status of *Te*, on the other hand, resembles the genome-downsized and host-obligated bordetellae. Its genome content has suffered such drastic decline that it has been left with very little option for further genome innovation. This is reflected in its high coding density, jeopardized DNA recombination and repair machinery, and associated paucity of paralogous genes. Consequently *Te* has got obligatorily confined within a critically specialized niche not by choice but due to compelling metabolic shortcomings taking toll on its sovereign existence in nature.

The case of *Tk* is unique because despite being a free-living chemolithoautotroph with no hitherto known report of intracellular existence, its genome is already on the wane, in addition to which it has also reserved the potential to switch to invasive lifestyle. Simple commonsense would deduce that the intrinsic degenerative trend of its genome (manifested in the form of an exorbitant number of pseudogenes) is the most potent factor that can anytime drive a natural population of *Tk* (or strains derived from *Tk*) pathogenic. The sweeping pseudogenization process underway in this genome primarily stems from its severely compromised DNA repair and recombination faculty. A host of DNA repair-recombination genes are themselves pseudogenization in *Tk*; their translations getting prevented in the first place must have had a global domino effect establishing frame-shift mutations across the genome. Notably, this impairment spree has been functionally indiscriminate and not directed towards any particular metabolic category. As such, potentially impaired *Tk* genes included 46 transcriptional regulator, 56 transporters (out of which 25 were ABC type), 18 other permeases, 10 major facilitator superfamily members, 47 dehydrogenases, 16 oxidoreductases, 14 hydrolases, six organic compound dioxygenases, five quinone and 10 cytochrome *c*-related genes, plus quite a few sensor kinases and response regulators. In addition to these elements crucial to *Tk*’s survival in its soil habitat, prospective host-interaction factors such as genes for LPS/exopolysaccharide biosynthesis, fimbrial biogenesis, hemagglutinin/hemolysin etc. have also been pseudogenized. This clearly implies that the reductive tendencies of the *Tk* genome are not environment-guided or adaptive in nature, but are rather intrinsic and inescapable properties of the genome itself. The most interesting aspect of this genome is that loss of metabolic functions through pseudogene formation has not yet led to any host-dependent niche restriction, as observed in other similar cases [10], [40], [45], [46], [47], [48]. In other words, loss of gene functions has not yet jeopardized *Tk*’s autonomous existence nor have such deficiencies been selected in a specific host environment. But the overall integrity of the *Tk* genome in any case is very delicately poised, if not critically endangered. This is apparent from the fact that genes crucial for genomic integrity, such as those encoding DNA gyrase subunit B, DNA topoisomerase IV subunit A, DNA polymerase III subunit epsilon, DNA helicase II, DNA-directed RNA polymerase subunit beta, chromosome partitioning protein ParB, and DNA primase have already been pseudogenized in *Tk*. And if this degenerative progression keeps reducing *Tk*’s gene content, as has already happened in case of *Te*, future survival and niche adaptation of such abjectly genome-decimated strains (or populations drifting out of the bottleneck) would essentially depend on what genomic resources are leftover in the aftermath of such devastation and what kind of eukaryotic tissue it can latch on to as a refuge. Following such impending primary host-adaptation, secondary or even tertiary bouts of narrower host-restriction can set in if the pseudogenization process continues unabated causing the genetic drift to widen and the niche width to narrow down further. In this scenario stockpiling of host-interaction factors in several environmentally competent strains of *Alcaligenaceae* (including *Tk* and A8) seems to be part of a preparedness plan to counter any imminent loss of the free-living ability arising out of the degradative dynamics of the genomes. Notably however, any future host-adaptation of *Tk* should essentially remain restricted to commensalism (as witnessed in case of *Te* [26]) and not involve lethal pathogenicity unless the drifted populations acquire toxic genetic factors from fellow infective agents in their newfound habitats.

We also need to appreciate that *Tk*, as we see it in our laboratory culture, is like a still photo-frame taken out from the fleeting movie (continuum) of evolution. As such, we really do not know whether in nature events similar to the above-envisaged scenario have already taken place in some yet-unidentified population of this species (or related species). But in case of such an eventuality, how *Tk*, as an organism, would look like can be gauged from the kind of existence *Te* is leading in its current state of evolution. With its DNA repair-recombination mechanism almost gone (thereby making new gene acquisition difficult), and there being virtually no scaffold (such as non-coding regions, paralogous gene copies, pseudogenes etc.) available for further genome innovation or improvisation, death knell of *Te* as an evolutionary lineage has already been tolled. However, the *Te* genome can take some heart from the fact that its pseudogenization onslaught has perhaps come to a halt, at least for the time being, or for that matter, at least in the population that was isolated as MCE9. So before extinction eventually sets in there will perhaps be a last phase of host-facilitated tranquility in the tumultuous life of this genome line.

## Materials and Methods

### Genome Sequencing, Assembly, and Annotation

The *Tk* genome was finished by the following strategy: (1) Deep sequencing was done on the Ion PGM Sequencer using an Ion 316 Sequencing Chip, following which an assembly of 898,717 reads (mean length 231 nucleotides) at an overall coverage of 37X using the MIRA 3.4.0 yielded 52 contigs. (2) Subsequently iterative scaffolding was done by HAPS (http://solidsoftwaretools.com/gf/project/haps/) using the current PGM data (52 ungapped or unpadded contigs) and the previously generated SOLiD 4 data (33,854,957 mate*-*paired 50 bp-long reads) [18]. This was executed in three stages: a) Error correction of the SOLiD 4 reads using SAET tool, b) Mapping & Pairing of the SOLiD 4 reads onto PGM contigs using Bioscope software, c) Scaffolding using SST tool (SOLiD Scaffolding Tools). Iterative scaffolding was performed to improve the results. As such, we first generated scaffolds using the 52 PGM contigs as reference, following which the obtained scaffolds were used as reference to perform the second round of scaffolding. The entire exercise generated seven scaffolds, out of which one represented a ∼ 59 Kb long plasmid and the remaining six the chromosome. (3) Gaps within the scaffolds were finally filled up by capillary sequencing.

Annotation of the *Tk* genome and COG ID assignment of the predicted PEGs was done using the NCBI's Prokaryotic Genomes Automatic Annotation Pipeline (PGAAP). This, together with the other compared genomes, was further processed and analyzed using the various tools available in the RAST annotation platform [20]. ORFs having at least one mutation preventing its translation were considered pseudogenes; all such inactivating mutation positions were reexamined in the original sequencing data using Tablet graphical viewer for next generation sequence assemblies and alignments.

All information pertaining to the *Tk* genome project is available in the GenBank under the BioProject PRJNA67337, while relevant deep-sequencing datasets are deposited in the NIH Short Read Archive (SRA) under the accession number SRP019065. As such, all the 972,013 reads (constituting 252 Mb *Tk* genome sequence) obtained using 200 bp chemistry on Ion Torrent PGM are available at the SRA under the accession number SRX249067, while the sum total of 101.6 million reads amounting to 10.2 Gb sequence data obtained using mate-pair chemistry on ABI SOLiD 4 system is available under the accession number SRX247703.

### Comparative Genomics

Annotated sequences were further collated and analyzed using Artemis [49] and RAST [20]. Comprehensive metabolic subsystem-wise tallying of the genome content of the bacteria in question was achieved manually. Genomes and predicted proteomes were compared at the sequence level using the web-based utility Double ACT (http://www.hpa-bioinfotools.org.uk/pise/double_act.html) in conjunction with the Artemis Comparison Tool [50]. Orthologous genes were identified on the basis of reciprocal best-hits in FASTA comparisons.

Genomes were analyzed for genes potentially acquired via HGT on the basis of DNA base composition and codon usage patterns [51], [52]. Regions having deviated G+C contents were identified by genome plots via Artemis [49]. Artemis was also used to select PEGs for codon count and Karlin signature plot that compares local dinucleotide composition within a sliding window relative to dinucleotide composition of the whole genome [53]. Codon Adaptation Index - which is a measure of codon usage deviation of a particular PEG from the genomic average [54] - was calculated by JCat [55].

For global colinearity analyses genomes were compared using the programs promer and mummer of Mummer 3.0, which uses a suffix tree algorithm to find maximal exact matches of minimum length between two input sequences [56]. All maximal unique matches between reference and query sequences on both the forward and reverse strands were identified and all the match positions relative to the forward strand were reported. These positions were then utilized to generate dot plots. In promer-based genome-wide co-linearity analyses six-frame translations of both genomes were compared and homologous regions plotted as dots that were color coded for percent similarity. Alternatively, in mummer, maximal exact matches of minimum nucleotide sequence lengths between two genomes were identified.

### Phylogenetic Analyses

Different gene sequence-based consensus trees were constructed after comparing the topologies of the relevant phylogenetic trees calculated by distance matrix, maximum parsimony and maximum likelihood analyses. Tools available in MEGA 4 and/or MEGA 5 were used for this purpose [57]. Trees were also re-constructed using tools available in the PHYLIP (Phylogeny Inference Package) version 3.69 distributed by J. Felsenstein, Department of Genome Sciences, University of Washington, Seattle.

### Hemolysis Assay with *Tk*

Washed *Tk* cell suspensions were prepared from overnight cultures in iron-repleted or iron-depleted minimal salts (MS) medium [58] supplemented with 5 gl^−1^ dextrose (MSD). These were mixed with washed 10^6^ hRBC in 1X phosphate-buffered saline (PBS) at various bacteria:RBC ratios (1∶1 to 1∶200), and incubated at 37°C for four hours. hRBC incubated without bacteria (negative control) and hRBC lysed with water (taken as the maximum possible level of lysis or positive control) constituted the two controls. To measure hemolysis quantitatively by flow cytometry-based analyses 10^6^ RBCs were washed three times by centrifugation at 9000*g* for 10 min, resuspended in PBS, and then infected with *Tk* cells for four hours in different MOIs (1∶1 to 1∶200). After infection the cell mixtures were washed in PBS thrice and subjected to flow cytometry measurements using a Becton Dickinson FACSCalibur™ flow cytometer. 20,000 cells (events) were evaluated for each measurement. At the outset we analyzed uninfected hRBCs to define the region of interest in forward side scatter versus sideward side scatter (FSC/SSC) dot plots. This gate setting was kept unchanged for all subsequent datasets. Quantitative comparisons were represented both as percentages of the gated events assigned to the different regions as well as the medians of the FSC/SSC ratios.

### Test of *Tk*’s Ability to Adhere to Eukaryotic Cells

Washed *Tk* or other bacterial cells were incubated overnight in FITC solution. Eukaryotic cells (HeLa [59] or Macrophage RAW264.7 [60]) were infected with the labeled bacteria at various MOIs (1∶1 to 1∶200) and incubated at 37°C for one hour. The bacteria-eukaryotic cell mixtures were then washed thrice with ice-cold PBS to remove unbound cells. The mixtures were then stained with 15 nM DAPI for one minute (to specifically target HeLa or RAW264.7 cells within the mixtures) and mounted in PBS/50% glycerol medium for immediate examination by a Zeiss LSM 510 Meta Confocal Microscope. Excitation of DNA-bound DAPI was done with a 360 nm UV argon laser, while FITC was excited with a 488 nm blue-green argon laser; fluorescence in the former case was detected at band pass (BP) 420–480 nm, whereas FITC emissions were detected at BP 505–530 nm.

## Supporting Information

File S1 **This file contains six tables designated as Table A through F, and three figures designated as Figure A through C.** Tables A, B, C and D respectively enumerate the genes predicted as derived from HGT in the genomes of *Tk*, A8, *Bb* and *Te*. Putative products of HGT were predicted on the basis of minimum 8–10% deviation from the average G+C content of the genome in question and/or more than 15–20% deviation from the average codon adaptation index of the genome. Table E enumerates those *Te* genes which are absent in at least one of the other three *Alcaligenaceae* genomes. Table F encompasses a list of the various protein degradation mechanisms potentially present in the four *Alcaligenaceae* in question. Figure A, B and C depict the syntenies of the gene clusters for T4SS, Tad transport system and DnaK heat shock chaperone respectively.
(DOC)

File S2 **A tabular comparison of the DNA metabolizing machineries of A8,** ***Bb*** **,** ***Tk*** **and** ***Te*** **has been included in this file.**
(DOC)

File S3 **This file includes a brief comparative genomic study of the secretion systems of the four** ***Alcaligenaceae*** **members in question.**
(DOC)

File S4 **Comparison of genes governing the development of lipopolysaccharide envelops and surface antigens in the studied** ***Alcaligenaceae*** **.**
(DOC)

File S5 **Comparative genomics of the iron uptake capabilities of the four** ***Alcaligenaceae*** **.**
(DOC)

## References

[1] Deveryshetty J, Phale PS (2010) Biodegradation of phenanthrene by *Alcaligenes* sp. strain PPH: partial purification and characterization of 1-hydroxy-2-naphthoic acid hydroxylase. FEMS Microbiol Lett 311: 93–101. 20727010 10.1111/j.1574-6968.2010.02079.x

[2] Essam T, Amin MA, El Tayeb O, Mattiasson B, Guieysse B (2010) Kinetics and metabolic versatility of highly tolerant phenol degrading *Alcaligenes* strain TW1. J Hazard Mater 173: 783–788. 19783362 10.1016/j.jhazmat.2009.09.006

[3] Mehdizadeh SN, Mehrnia MR, Abdi K, Sarrafzadeh MH (2011) Biological treatment of toluene contaminated wastewater by *Alcaligenese faecalis* in an extractive membrane bioreactor; experiments and modeling. Water Sci Technol 64: 1239–1246. 22214076 10.2166/wst.2011.756

[4] Uhlik O, Jecna K, Mackova M, Vlcek C, Hroudova M, et al (2009) Biphenyl-metabolizing bacteria in the rhizosphere of horseradish and bulk soil contaminated by polychlorinated biphenyls as revealed by stable isotope probing. Appl Environ Microbiol 75: 6471–6477. 19700551 10.1128/AEM.00466-09 PMC2765145

[5] Aisenberg G, Rolston KV, Safdar A (2004) Bacteremia caused by *Achromobacter* and *Alcaligenes* species in 46 patients with cancer (1989–2003). Cancer 101: 2134–2140. 15389476 10.1002/cncr.20604

[6] Ghosh W, Bagchi A, Mandal S, Dam B, Roy P (2005) *Tetrathiobacter kashmirensis* gen. nov., sp. nov., a novel mesophilic, neutrophilic, tetrathionate-oxidizing, facultatively chemolithotrophic betaproteobacterium isolated from soil from a temperate orchard in Jammu and Kashmir, India. Int J Syst Evol Microbiol 55: 1779–1787. 16166666 10.1099/ijs.0.63595-0

[7] Gibello A, Vela AI, Martin M, Barra-Caracciolo A, Grenni P, et al (2009) Reclassification of the members of the genus *Tetrathiobacter* Ghosh et al. 2005 to the genus *Advenella* Coenye et al. 2005. Int J Syst Evol Microbiol 59: 1914–1918. 19567588 10.1099/ijs.0.007443-0

[8] Sebaihia M, Preston A, Maskell DJ, Kuzmiak H, Connell TD, et al (2006) Comparison of the genome sequence of the poultry pathogen *Bordetella avium* with those of *B*. *bronchiseptica*, *B*. *pertussis*, and *B*. *parapertussis* reveals extensive diversity in surface structures associated with host interaction. J Bacteriol 188: 6002–6015. 16885469 10.1128/JB.01927-05 PMC1540077

[9] Jang SS, Donahue JM, Arata AB, Goris J, Hansen LM, et al (2001) *Taylorella asinigenitalis* sp. nov., a bacterium isolated from the genital tract of male donkeys (Equus asinus). Int J Syst Evol Microbiol 51: 971–976. 11411723 10.1099/00207713-51-3-971

[10] Parkhill J, Sebaihia M, Preston A, Murphy LD, Thomson N, et al (2003) Comparative analysis of the genome sequences of *Bordetella pertussis*, *Bordetella parapertussis* and *Bordetella bronchiseptica* . Nat Genet 35: 32–40. 12910271 10.1038/ng1227

[11] Hebert L, Moumen B, Duquesne F, Breuil MF, Laugier C, et al (2011) Genome sequence of *Taylorella equigenitalis* MCE9, the causative agent of contagious equine metritis. J Bacteriol 193: 1785. 21278298 10.1128/JB.01547-10 PMC3067654

[12] Ahmed N, Dobrindt U, Hacker J, Hasnain SE (2008) Genomic fluidity and pathogenic bacteria: applications in diagnostics, epidemiology and intervention. Nat Rev Microbiol 6: 387–394. 18392032 10.1038/nrmicro1889

[13] Eppinger M, Baar C, Raddatz G, Huson DH, Schuster SC (2004) Comparative analysis of four *Campylobacterales* . Nat Rev Microbiol 2: 872–885. 15494744 10.1038/nrmicro1024

[14] Cummings CA, Brinig MM, Lepp PW, van de Pas S, Relman DA (2004) *Bordetella* species are distinguished by patterns of substantial gene loss and host adaptation. J Bacteriol 186: 1484–1492. 14973121 10.1128/JB.186.5.1484-1492.2004 PMC344407

[15] Diavatopoulos DA, Cummings CA, Schouls LM, Brinig MM, Relman DA, et al (2005) *Bordetella pertussis*, the causative agent of whooping cough, evolved from a distinct, human-associated lineage of B. bronchiseptica. PLoS Pathog 1: e45. 16389302 10.1371/journal.ppat.0010045 PMC1323478

[16] van der Zee A, Mooi F, Van Embden J, Musser J (1997) Molecular evolution and host adaptation of *Bordetella* spp.: phylogenetic analysis using multilocus enzyme electrophoresis and typing with three insertion sequences. J Bacteriol 179: 6609–6617. 9352907 10.1128/jb.179.21.6609-6617.1997 PMC179586

[17] Strnad H, Ridl J, Paces J, Kolar M, Vlcek C, et al (2011) Complete genome sequence of the haloaromatic acid-degrading bacterium *Achromobacter xylosoxidans* A8. J Bacteriol 193: 791–792. 21097610 10.1128/JB.01299-10 PMC3021230

[18] Ghosh W, George A, Agarwal A, Raj P, Alam M, et al (2011) Whole-genome shotgun sequencing of the sulfur-oxidizing chemoautotroph *Tetrathiobacter kashmirensis* . J Bacteriol 193: 5553–5554. 21914874 10.1128/JB.05781-11 PMC3187427

[19] Dam B, Ghosh W, Das Gupta SK (2009) Conjugative Type 4 secretion system of a novel large plasmid from the chemoautotroph *Tetrathiobacter kashmirensis* and construction of shuttle vectors for Alcaligenaceae. Appl Environ Microbiol 75: 4362–4373. 19411426 10.1128/AEM.02521-08 PMC2704820

[20] Aziz RK, Bartels D, Best AA, DeJongh M, Disz T, et al (2008) The RAST Server: rapid annotations using subsystems technology. BMC Genomics 9: 75. 18261238 10.1186/1471-2164-9-75 PMC2265698

[21] Yuk MH, Harvill ET, Cotter PA, Miller JF (2000) Modulation of host immune responses, induction of apoptosis and inhibition of NF-kappaB activation by the *Bordetella* type III secretion system. Mol Microbiol 35: 991–1004. 10712682 10.1046/j.1365-2958.2000.01785.x

[22] Henderson IR, Navarro-Garcia F, Desvaux M, Fernandez RC, Ala'Aldeen D (2004) Type V protein secretion pathway: the autotransporter story. Microbiol Mol Biol Rev 68: 692–744. 15590781 10.1128/MMBR.68.4.692-744.2004 PMC539010

[23] Bador J, Amoureux L, Duez JM, Drabowicz A, Siebor E, et al (2011) First description of an RND-type multidrug efflux pump in *Achromobacter xylosoxidans*, AxyABM. Antimicrob Agents Chemother 55: 4912–4914. 21807978 10.1128/AAC.00341-11 PMC3186983

[24] Spilker T, Vandamme P, Lipuma JJ (2012) A multilocus sequence typing scheme implies population structure and reveals several putative novel *Achromobacter* species. J Clin Microbiol 50: 3010–3015. 22785192 10.1128/JCM.00814-12 PMC3421806

[25] Hebert L, Moumen B, Pons N, Duquesne F, Breuil MF, et al (2012) Genomic characterization of the *Taylorella* genus. PLoS One 7: e29953. 22235352 10.1371/journal.pone.0029953 PMC3250509

[26] Matsuda M, Moore JE (2003) Recent advances in molecular epidemiology and detection of *Taylorella equigenitalis* associated with contagious equine metritis (CEM). Vet Microbiol 97: 111–122. 14637043 10.1016/j.vetmic.2003.08.001

[27] Robert PY, Chainier D, Garnier F, Ploy MC, Parneix P, et al (2008) *Alcaligenes xylosoxidans* endophthalmitis following phacoemulsification and intraocular lens implantation. Ophthalmic Surg Lasers Imaging 39: 500–504. 19065983 10.3928/15428877-20081101-15

[28] Brickman TJ, Cummings CA, Liew SY, Relman DA, Armstrong SK (2011) Transcriptional profiling of the iron starvation response in *Bordetella pertussis* provides new insights into siderophore utilization and virulence gene expression. J Bacteriol 193: 4798–4812. 21742863 10.1128/JB.05136-11 PMC3165686

[29] Nakamura MM, Liew SY, Cummings CA, Brinig MM, Dieterich C, et al (2006) Growth phase- and nutrient limitation-associated transcript abundance regulation in *Bordetella pertussis* . Infect Immun 74: 5537–5548. 16988229 10.1128/IAI.00781-06 PMC1594893

[30] Remenant B, de Cambiaire JC, Cellier G, Jacobs JM, Mangenot S, et al (2011) Ralstonia syzygii, the Blood Disease Bacterium and some Asian *R*. *solanacearum* strains form a single genomic species despite divergent lifestyles. PLoS One 6: e24356. 21931687 10.1371/journal.pone.0024356 PMC3169583

[31] Meidanis J, Braga MD, Verjovski-Almeida S (2002) Whole-genome analysis of transporters in the plant pathogen *Xylella fastidiosa* . Microbiol Mol Biol Rev 66: 272–299. 12040127 10.1128/MMBR.66.2.272-299.2002 PMC120790

[32] Redondo-Nieto M, Barret M, Morrisey JP, Germaine K, Martinez-Granero F, et al (2012) Genome sequence of the biocontrol strain *Pseudomonas fluorescens* F113. J Bacteriol 194: 1273–1274. 22328765 10.1128/JB.06601-11 PMC3294817

[33] Carsiotis M, Stocker BA, Weinstein DL, O'Brien AD (1989) A *Salmonella typhimurium* virulence gene linked to flg. Infect Immun 57: 3276–3280. 2680969 10.1128/iai.57.11.3276-3280.1989 PMC259797

[34] Rudnick PA, Arcondeguy T, Kennedy CK, Kahn D (2001) *glnD* and *mviN* are genes of an essential operon in *Sinorhizobium meliloti* . J Bacteriol 183: 2682–2685. 11274131 10.1128/JB.183.8.2682-2685.2001 PMC95188

[35] O'Connell KP, Raffel SJ, Saville BJ, Handelsman J (1998) Mutants of *Rhizobium tropici* strain CIAT899 that do not induce chlorosis in plants. Microbiology 144: 2607–2617. 9782510 10.1099/00221287-144-9-2607

[36] Baida GE, Kuzmin NP (1996) Mechanism of action of hemolysin III from *Bacillus cereus* . Biochim Biophys Acta 1284: 122–124. 8962879 10.1016/s0005-2736(96)00168-x

[37] Kampfer P, Falsen E, Langer S, Lodders N, Busse HJ (2010) *Paenalcaligenes hominis* gen. nov., sp. nov., a new member of the family *Alcaligenaceae* . Int J Syst Evol Microbiol 60: 1537–1542. 19684310 10.1099/ijs.0.016576-0

[38] Srinivasan S, Kim MK, Sathiyaraj G, Kim YJ, Yang DC (2010) *Pusillimonas ginsengisoli* sp. nov., isolated from soil of a ginseng field. Int J Syst Evol Microbiol 60: 1783–1787. 19749028 10.1099/ijs.0.018358-0

[39] Stolz A, Burger S, Kuhm A, Kampfer P, Busse HJ (2005) *Pusillimonas noertemannii* gen. nov., sp. nov., a new member of the family *Alcaligenaceae* that degrades substituted salicylates. Int J Syst Evol Microbiol 55: 1077–1081. 15879236 10.1099/ijs.0.63466-0

[40] Cole ST, Eiglmeier K, Parkhill J, James KD, Thomson NR, et al (2001) Massive gene decay in the leprosy bacillus. Nature 409: 1007–1011. 11234002 10.1038/35059006

[41] Ahmed MS, Nistal C, Jayan R, Kuduvalli M, Anijeet HK (2009) *Achromobacter xylosoxidans*, an emerging pathogen in catheter-related infection in dialysis population causing prosthetic valve endocarditis: a case report and review of literature. Clin Nephrol 71: 350–354. 19281752 10.5414/cnp71350

[42] Traglia GM, Almuzara M, Merkier AK, Adams C, Galanternik L, et al (2012) *Achromobacter xylosoxidans*: An Emerging Pathogen Carrying Different Elements Involved in Horizontal Genetic Transfer. Curr Microbiol 65: 673–678. 22926720 10.1007/s00284-012-0213-5 PMC3477587

[43] Taylor JS, Raes J (2004) Duplication and divergence: the evolution of new genes and old ideas. Annu Rev Genet 38: 615–643. 15568988 10.1146/annurev.genet.38.072902.092831

[44] Ghosh W, Mallick S, DasGupta SK (2009) Origin of the Sox multienzyme complex system in ancient thermophilic bacteria and coevolution of its constituent proteins. Res Microbiol 160: 409–420. 19616092 10.1016/j.resmic.2009.07.003

[45] Parkhill J, Dougan G, James KD, Thomson NR, Pickard D, et al (2001) Complete genome sequence of a multiple drug resistant *Salmonella enterica* serovar Typhi CT18. Nature 413: 848–852. 11677608 10.1038/35101607

[46] Andersson JO, Andersson SG (1999) Genome degradation is an ongoing process in *Rickettsia* . Mol Biol Evol 16: 1178–1191. 10486973 10.1093/oxfordjournals.molbev.a026208

[47] McClelland M, Sanderson KE, Clifton SW, Latreille P, Porwollik S, et al (2004) Comparison of genome degradation in Paratyphi A and Typhi, human-restricted serovars of *Salmonella enterica* that cause typhoid. Nat Genet 36: 1268–1274. 15531882 10.1038/ng1470

[48] Thomson NR, Howard S, Wren BW, Holden MT, Crossman L, et al (2006) The complete genome sequence and comparative genome analysis of the high pathogenicity *Yersinia enterocolitica* strain 8081. PLoS Genet 2: e206. 17173484 10.1371/journal.pgen.0020206 PMC1698947

[49] Rutherford K, Parkhill J, Crook J, Horsnell T, Rice P, et al (2000) Artemis: sequence visualization and annotation. Bioinformatics 16: 944–945. 11120685 10.1093/bioinformatics/16.10.944

[50] Carver TJ, Rutherford KM, Berriman M, Rajandream MA, Barrell BG, et al (2005) ACT: the Artemis Comparison Tool. Bioinformatics 21: 3422–3423. 15976072 10.1093/bioinformatics/bti553

[51] Lawrence JG, Ochman H (1998) Molecular archaeology of the *Escherichia coli* genome. Proc Natl Acad Sci U S A 95: 9413–9417. 9689094 10.1073/pnas.95.16.9413 PMC21352

[52] Shi SY, Cai XH, Ding DF (2005) Identification and categorization of horizontally transferred genes in prokaryotic genomes. Acta Biochim Biophys Sin (Shanghai) 37: 561–566. 16077904 10.1111/j.1745-7270.2005.00075.x

[53] Karlin S, Campbell AM, Mrazek J (1998) Comparative DNA analysis across diverse genomes. Annu Rev Genet 32: 185–225. 9928479 10.1146/annurev.genet.32.1.185

[54] Sharp PM, Li WH (1987) The codon Adaptation Index-a measure of directional synonymous codon usage bias, and its potential applications. Nucleic Acids Res 15: 1281–1295. 3547335 10.1093/nar/15.3.1281 PMC340524

[55] Grote A, Hiller K, Scheer M, Munch R, Nortemann B, et al (2005) JCat: a novel tool to adapt codon usage of a target gene to its potential expression host. Nucleic Acids Res 33: W526–531. 15980527 10.1093/nar/gki376 PMC1160137

[56] Kurtz S, Phillippy A, Delcher AL, Smoot M, Shumway M, et al (2004) Versatile and open software for comparing large genomes. Genome Biol 5: R12. 14759262 10.1186/gb-2004-5-2-r12 PMC395750

[57] Kumar S, Nei M, Dudley J, Tamura K (2008) MEGA: a biologist-centric software for evolutionary analysis of DNA and protein sequences. Brief Bioinform 9: 299–306. 18417537 10.1093/bib/bbn017 PMC2562624

[58] Ghosh W, Roy P (2006) *Mesorhizobium thiogangeticum* sp. nov., a novel sulfur-oxidizing chemolithoautotroph from rhizosphere soil of an Indian tropical leguminous plant. Int J Syst Evol Microbiol 56: 91–97. 16403872 10.1099/ijs.0.63967-0

[59] Scherer WF, Syverton JT, Gey GO (1953) Studies on the propagation in vitro of poliomyelitis viruses. IV. Viral multiplication in a stable strain of human malignant epithelial cells (strain HeLa) derived from an epidermoid carcinoma of the cervix. J Exp Med 97: 695–710. 13052828 10.1084/jem.97.5.695 PMC2136303

[60] Raschke WC, Baird S, Ralph P, Nakoinz I (1978) Functional macrophage cell lines transformed by Abelson leukemia virus. Cell 15: 261–267. 212198 10.1016/0092-8674(78)90101-0

