# Supplementary material for: Genome Implosion Elicits Host-Confinement in Alcaligenaceae: Evidence from the Comparative Genomics of Tetrathiobacter kashmirensis, a Pathogen in the Making
Source: PLoS One. 2013 May 31;8(5):e64856. doi: 10.1371/journal.pone.0064856 (PMC3669393; doi:10.1371/journal.pone.0064856)
Supplement: File S1 — This file contains six tables designated as Table A through F, and three figures designated as Figure A through C. Tables A, B, C and D respectively enumerate the genes predicted as derived from HGT in the genomes of Tk, A8, Bb and Te. Putative products of HGT were predicted on the basis of minimum 8–10% deviation from the average G+C content of the genome in question and/or more than 15–20% deviation from the average codon adaptation index of the genome. Table E enumerates those Te genes which are absent in at least one of the other three Alcaligenaceae genomes. Table F encompasses a list of the various protein degradation mechanisms potentially present in the four Alcaligenaceae in question. Figure A, B and C depict the syntenies of the gene clusters for T4SS, Tad transport system and DnaK heat shock chaperone respectively. (DOC) [file pone.0064856.s001.doc]

**Table A.** G+C content and Codon Adaptation Index (CAI) of the regions in the *Tetrathiobacter kashmirensis* WT001^T^ genome predicted to have been acquired via HGT. The average G+C % of the genome is 55.88. The mean CAI of the genome is 0.543.

| **Approximate position**  **[Length of total HGT in bp]** | **Locus Tags** | **BLASTp hits** | **G+C %** | **MCAI** |
| --- | --- | --- | --- | --- |
| 6052 – 7556  [1505] | TKWG_00025  +  TKWG_00030 | **Permeases of the drug/metabolite transporter (DMT) superfamily** [67%, *Yersinia intermedia* ATCC 29909]  +  **TetR family transcriptional regulator** [66%, *Yersinia intermedia* ATCC 29909] | 48  +  44 | 0.279  +  0.312 |
| 13783 – 14639  [857] | TKWG_00060  +  TKWG_00065 | **TetR family transcriptional regulator** [59%, *Cronobacter turicensis* z3032]  +  **Hypothetical protein** [47%, *Bacteroides plebeius* DSM 17135] | 46  +  43 | 0.475  +  0.311 |
| 18999 – 20224  [1226] | Pseudogene | **Branched chain amino-acid ABC transporter substrate-binding protein** [87%, *Bordetella parapertussis* 12822] | 49 | - |
| 20541 – 20999  [459] | TKWG_00115 | **COG1917 Uncharacterized protein** [89%, *Bordetella bronchiseptica* RB50] | 49 | 0.499 |
| 116017 – 116325  [309] | TKWG_00640 | **COG2146 Ferredoxin subunit of nitrite reductase and ring–hydroxylating dioxygenase** [58%, *Cupriavidus necator* N–1] | 49 | 0.596 |
| 130717–131441  [725] | TKWG_00755  +  TKWG_00760 | **CsbD like protein** [55%, *Pseudoalteromonas atlantica* T6c]  +  **Hypothetical protein** [67%, *Hirschia baltica* ATCC 49814] | 50  +  50 | 0.538  +  0.581 |
| 456843 – 457729  [887] | TKWG_02635  +  TKWG_02640 | **Hypothetical protein** [38%, *Acidovorax radicis* N35]  +  **Hypothetical protein** [44%, *Ralstonia solanacearum* GMI1000] | 49  +  44 | 0.523  +  0.507 |
| 627753 – 628673  [921] | TKWG_03665 | **LysR family transcriptional regulator** [40%, *Achromobacter xylosoxidans* C54] | 48 | 0.443 |
| 724167–724694  [528] | TKWG_04210 | **Lipoprotein** [53%, *Bordetella petrii* DSM 12804] | 50 | 0.597 |
| 850463 – 850537  [75] | TKWG_t25434 | **tRNA–Arg** | 48 | 0.360 |
| 862167 – 862958  [792] | TKWG_04965 | **COG0784 CheY–like receiver_AraC family transcriptional regulator** [35%,  *Burkholderia phytofirmans* PsJN] | 46 | 0.468 |
| 1087286–1087914  [629] | TKWG_06335  +  TKWG_06340 | **Eggshell protein p48** [63%, *Chlamydia trachomatis* B/Jali20/OT; 65%,  *Bordetella pertussis* Tohama I]  +  **Hypothetical protein** [65%, eggshell protein p48 of *Chlamydophila felis* Fe/C–56] | 49  +  42 | 0.504  +  0.535 |
| 1108580 – 1109193  [614] | TKWG_06460  +  TKWG_06465 | **Addiction module killer protein** [57%,  *Haemophilus somnus* 2336]  +  **COG3636 Predicted transcriptional regulator** [66%, *Desulfonatronospira thiodismutans* ASO3–1] | 46  +  44 | 0.430  +  0.391 |
| 1123246 – 1124136  [891] | TKWG_06545 | **Chromosome segregation ATPase** [27%, *Agrobacterium radiobacter* K84] | 45 | 0.533 |
| 1262517 – 1263236  [720] | TKWG_07310 | **COG3121 P pilus assembly protein, chaperone PapD** [39%, *Variovorax paradoxus* S110] | 48 | 0.421 |
| 1351047–1352494  [1448] | TKWG_07820 | **Hypothetical protein** [30%, *Trypanosoma brucei* *brucei* strain 927/4 GUTat10.1] | 44 | 0.568 |
| 1369631–1370158  [528] | TKWG_07925 | **Fimbrial protein** [45%, *Acinetobacter baumannii* ABNIH3] | 50 | 0.504 |
| 1476173–1477216  [1044] | TKWG_08555 | **Phosphate ABC transport** [82%, *Achromobacter piechaudii* ATCC 43553] | 50 | 0.563 |
| 1511827 – 1512105  [279] | TKWG_08760 | **Hypothetical protein** [31%, *Photorhabdus luminescens* TTO1] | 42 | 0.494 |
| 1553665 – 1554087  [423] | TKWG_09000 | **Hypothetical protein** [62%, *Achromobacter xylosoxidans* A8] | 48 | 0.525 |
| 1616569 – 1617774  [1206] | TKWG_09315 | **COG3203 Outer membrane protein (porin)**  [42%, *Achromobacter xylosoxidans* A8] | 48 | 0.439 |
| 1629262 – 1630461  [1200] | TKWG_09370 | **COG3203 Outer membrane protein (porin)**  [45%, *Achromobacter xylosoxidans* A8] | 48 | 0.512 |
| 1712498 – 1712734  [237] | TKWG_09870 | **COG0236 Acyl carrier protein** [99%, *Bordetella petrii* DSM 12804] | 43 | 0.545 |
| 1814706 – 1816046  [1341] | TKWG_10475 | **Hypothetical protein** [29%, *Shewanella violacea* DSS12] | 44 | 0.395 |
| 2074496 – 2076252  [1757] | Pseudogene  +  TKWG_11970 | **Potential frameshift– Sterol desaturase** [93%, *Bordetella petrii* DSM 12804]  +  **COG0454 Histone acetyltransferase HPA2 and related acetyltransferases** [29%, *Acaryochloris marina* MBIC11017] | 51  +  49 | -  +  0.360 |
| 2189029 – 2189469  [441] | TKWG_12605 | **Hypothetical protein** [38%, *Achromobacter xylosoxidans* C54] | 48 | 0.592 |
| 2243983 – 2244192  [210] | TKWG_12915 | **COG1278 Cold shock proteins** [80%, *Pusillimonas* spp.; 67%, *Bordetella avium* 197N] | 44 | 0.564 |
| 2280550 – 2285271  [4722] | TKWG_13130 to TKWG_13165 | **Hypothetical protein** [32%, *Pseudomonas stutzeri* TS44]  +  **Hypothetical protein** [28%, *Bacteroides cellulosilyticus* DSM 14838]  +  **Hypothetical protein** [43%, *Pectobacterium carotovorum* PC1]  +  **Phage integrase family protein** [67%, *Haemophilus parainfluenzae* T3T1]  +  **Xylulose–5–phosphate/fructose–6–phosphate phosphoketolase** [75%, *Pasteurella multocida* HN06]  +  **Plasmid maintenance system antidote protein, XRE family/addiction module antidote protein, HigA family / PSEEN1946 proteic killer active protein** [70%, *Aggregatibacter actinomycetemcomitans* SC1083]  +  **Plasmid maintenance system antidote protein**  [68%, *Rhodoferax ferrireducens* T118]  +  **Plasmid maintenance system killer protein** [52%, *Comamonas testosteroni* CNB–2] | 41  +  41  +  44  +  52  +  45  +  50  +  47  +  48 | 0.380  +  0.386  +  0.348  +  0.421  +  0.551  +  0.348  +  0.524  +  0.423 |
| 2286657 – 2290266  [3610] | TKWG_13185  +  TKWG_13190  +  TKWG_13195 | **Hypothetical protein** [39%, *Pseudomonas syringae* M302091]  +  **Hypothetical protein** [32%, *Prevotella oulorum* F0390]  +  **Short–chain dehydrogenase; 3–oxoacyl–ACP reductase** [65%, *Cupriavidus metallidurans* CH34] | 42  +  40  +  50 | 0.319  +  0.412  +  0.341 |
| 2292048 – 2294905  [2858] | TKWG_13210 to TKWG_13225 | **Hypothetical protein** [37%, *Ralstonia eutropha* JMP134]  +  **COG0183 Acetyl–CoA acetyltransferase** [66%, *Bordetella bronchiseptica* RB50]  +  **Hypothetical protein** [31%, TetR family transcriptional regulator, *Enhydrobacter aerosaccus* SK60]  +  **TetR family transcriptional regulator** [30%, *Desulfatibacillum alkenivorans* AK–01] | 46  +  50  +  43  +  44 | 0.416  +  0.374  +  0.357  +  0.400 |
| 2295983 – 2298588  [2606] | TKWG_13235 to TKWG_13245 | **Hypothetical protein** [30%, *Vibrio parahaemolyticus* AQ3810]  +  **Uracil–DNA glycosylase superfamily protein**  [38%, *Chlorobium phaeobacteroides* DSM 266]  +  **Hypothetical protein** [27%, *Starkeya novella* DSM 506] | 39  +  45  +  43 | 0.298  +  0.376  +  0.320 |
| 2301205 – 2302434  [1230] | TKWG_13265  +  TKWG_13270 | **Hypothetical protein** [33%, *Bacteroides fragilis* NCTC 9343]  +  **Hypothetical protein** [57%, *Burkholderia terrae* BS001] | 45  +  41 | 0.355  +  0.430 |
| 2366641–2367219  [579] | TKWG_13670 | **4–carboxymuconolactone decarboxylase** [81%, *Burkholderia ambifaria* AMMD] | 49 | 0.537 |
| 2393372–2394667  [1296] | TKWG_13830 | **carboxyl transferase domain–containing protein** [75%, *Achromobacter xylosoxidans* A8] | 50 | 0.415 |
| 2407082–2407510  [429] | TKWG_13900 | **hypothetical protein** [28%, *Cronobacter sakazakii* ES15] | 44 | 0.394 |
| 2411644–2412183  [540] | TKWG_13930 | **hypothetical protein** [56%, *Citrobacter rodentium* ICC168] | 46 | 0.419 |
| 2451651–2451854  [204] | TKWG_14145 | **hypothetical protein** [32%, *Burkholderia ambifaria* MEX-5] | 43 | 0.504 |
| 2459375 – 2463506  [4132] | TKWG_14215  +  TKWG_14220  +  TKWG_14225 | **COG1217 Predicted membrane GTPase involved in stress response** [45%, *Acetobacter tropicalis* NBRC 101654]  +  **Hypothetical protein** [44%, *Acetobacter tropicalis* NBRC 101654]  +  **Hypothetical protein** [45%, *Acidiphilium cryptum* JF–5] | 46  +  45  +  47 | 0.490  +  0.399  +  0.375 |
| 2617591–2617863  [273] | TKWG_15170 | **DNA–binding protein HU–beta** [89%, *Bordetella pertussis* Tohama I] | 48 | 0.519 |
| 2785741–2786631  [891] | TKWG_16185 | **Hypothetical protein** [53%, *Geobacter metallireducens* GS-15] | 49 | 0.570 |
| 2798982 – 2800196  [1215] | TKWG_t25480  +  TKWG_16260 | **tRNA–Met**  +  **Hypothetical protein** [32%, *Streptomyces flavogriseus* ATCC 33331] | 64  +  44 | 0.555  +  0.468 |
| 3125480—3125689  [210] | TKWG_18145 | **Hypothetical protein** [35%, *Bacillus subtilis* subsp. spizizenii ATCC 6633] | 43 | 0.609 |
| 3232271–3232546  [276] | TKWG_18775 | **Hypothetical protein** [88%, *Alcaligenes faecalis* subsp. faecalis NCIB 8687] | 47 | 0.606 |
| 3462811–3463845  [1035] | TKWG_20090 | **AraC family transcriptional regulator** [59%, *Methylophaga thiooxidans* DMS010] | 47 | 0.438 |
| 3577500 – 3582003  [4504] | TKWG_20820 to TKWG_20840 | **Hypothetical protein** [37%, *Coprococcus comes* ATCC 27758]  +  **Hypothetical protein** [33%, *Xanthomonas albilineans* GPE PC73]  +  **Putative transcriptional regulator** [54%, *Agrobacterium tumefaciens* 5A]  +  **Conjugal transfer protein VirB6** [56%, *Salmonella enterica* Dublin]  +  **Conjugal transfer protein VirB5** [59%, *Providencia rettgeri*] | 41  +  46  +  43  +  47  +  47 | 0.284  +  0.345  +  0.500  +  0.327  +  0.390 |
| 3591411–3591950  [540] | TKWG_20885 | **Hypothetical protein** [28%, *Leeuwenhoekiella blandensis* MED217] | 42 | 0.404 |
| 3594216 – 3596939  [2724] | TKWG_20905 to TKWG_20915 | **Hypothetical protein** [29%, *Bordetella bronchiseptica* RB50]  +  **COG4271 Predicted nucleotide–binding protein containing TIR–like domain** [53%, *Burkholderia pseudomallei* 7894]  +  **COG0362 6–phosphogluconate dehydrogenase** [63%, *Pseudoalteromonas haloplanktis* ANT/505] | 41  +  48  +  47 | 0.381  +  0.326  +  0.409 |
| 3599125 – 3605996  [6872] | TKWG_20935 to TKWG_20950 | **Hypothetical protein** [67%, *Burkholderia multivorans* ATCC 17616]  +  **Hypothetical protein** [27%, *Ruminococcus torques* L2–14]  +  **Hypothetical protein** [39%, *Lactobacillus brevis* ATCC 367]  +  **Hypothetical protein** [26%, *Ktedonobacter racemifer* DSM 44963] | 54  +  35  +  32  +  43 | 0.331  +  0.295  +  0.502  +  0.366 |
| 3611398–3613927  [2530] | TKWG_21000  +  TKWG_21005 | **Hypothetical protein** [64%, *Methylobacterium radiotolerans* JCM 2831]  +  **Hypothetical protein** [42%, *Achromobacter piechaudii* ATCC 43553] | 47  +  48 | 0.451  +  0.361 |
| 3623238–3624221  [983] | TKWG_21070 | **Hypothetical protein** [85%, *Achromobacter xylosoxidans* A8] | 48 | 0.547 |
| 3647149–3649460  [2312] | TKWG_21210 to  TKWG_21220 | **Hypothetical protein** [35%, *Chitinophaga pinensis* DSM 2588]  +  **Hypothetical protein** [52%, *Shewanella baltica* OS183]  +  **Restriction endonuclease** [69%, *Escherichia coli* H730] | 41  +  49  +  49 | 0.406  +  0.361  +  0.361 |
| 3679176–3680838  [1663] | TKWG_21400  +  Pseudogene | **ABC transporter ATP–binding protein/permease** [49%, *Acinetobacter baumannii* AB900]  +  **ABC–type transporter** [39%, *Escherichia coli* MS 196-1] | 45  +  46 | 0.428  +  - |
| 3683841–3686488  [2648] | TKWG_21440 to  TKWG_21455 | **Hypothetical protein** [29%, *Dethiobacter alkaliphilus* AHT 1]  +  **Hypothetical protein** [31%, *Escherichia coli* M718]  +  **Hypothetical protein** [48%, *Pectobacterium atrosepticum* SCRI1043]  +  **Hypothetical protein** [27%, *Pseudoalteromonas rubra* ATCC 29570] | 41  +  46  +  37  +  32 | 0.302  +  0.449  +  0.231  +  0.390 |
| 3697750–3699328  [1579] | TKWG_21490 to  TKWG_21500 | **Hypothetical protein** [28%, *Gordonibacter pamelaeae* 7-10-1-b]  +  **Hypothetical protein** [28%, *Populus trichocarpa*] [37%, *Tetraodon nigroviridis*]  +  **Hpothetical protein** [41%, *Myxococcus xanthus* DK 1622] | 46  +  43  +  41 | 0.402  +  0.445  +  0.429 |
| 3752975–3753844  [870] | TKWG_21845 | **Taurine catabolism dioxygenase tauD/tfdA** [46%, *Sphingobium chlorophenolicum* L-1] | 49 | 0.468 |
| 3759511–3767554  [8044] | TKWG_21885 to  TKWG_21920 | **Hypothetical protein** [72%, *Bordetella petrii* DSM 12804]  +  **Hypothetical protein** [42%, South polar skua adenovirus-1] [39%, *Plasmodium knowlesi* strain H]  +  **Hypothetical protein** [29%, *Collinsella tanakaei* YIT 12063]  +  **Hypothetical protein** [28%, *Oryza sativa* Japonica Group], [31%, Saccharopolyspora erythraea NRRL 2338]  +  **Hypothetical protein** [31%, *Bordetella petrii* DSM 12804]  +  **Pentapeptide repeat–containing protein** [28%, *Variovorax paradoxus* EPS]  +  **Hypothetical protein** [40%, *Variovorax paradoxus* S110]  +  **Hypothetical protein** [31%, *Halomicrobium mukohataei* DSM 12286] | 51  +  42  +  43  +  39  +  44  +  44  +  43  +  41 | 0.548  +  0.445  +  0.423  +  0.417  +  0.429  +  0.431  +  0.451  +  0.420 |
| 3926680 – 3928658  [1979] | TKWG_22890  +  TKWG_22895  +  TKWG_22900 | **Hypothetical phage–associated protein** [46%, *Burkholderia* phage phi52237]  +  **COG3600 Uncharacterized phage–associated protein** [54%, *Burkholderia* phage phi52237]  +  **Hypothetical protein** [39%, *Trichophyton equinum* CBS 127.97] | 40  +  39  +  50 | 0.358  +  0.35  +  0.371 |
| 4002082 – 4005472  [3391] | TKWG_23325  +  TKWG_23330 | **Hypothetical protein** [36%, *Agrobacterium radiobacter* K84]  +  **COG0836 Mannose–1–phosphate guanylyltransferase** [59%, *Xanthomonas vesicatoria* ATCC 35937] | 50  +  50 | 0.518  +  0.457 |
| 4009008 – 4030347  [21340] | TKWG_23345 to TKWG_23420 | **Hypothetical protein** [22%, *Helcococcus kunzii* ATCC 51366]  +  **FkbM family methyltransferase** [46%, *Burkholderia ambifaria* MEX-5]  +  **COG3013 Uncharacterized conserved protein** [37%, *Cupriavidus necator* N-1]  +  **Hypothetical protein** [28%, *Bacillus cereus* W]  +  **Hypothetical protein** [27%, *Francisella philomiragia* ATCC 25015]  +  **Serine acetyltransferase** [44%, *Pseudoalteromonas arctica* A 37–1–2]  +  **Hypothetical protein** [32%, *Bacillus cereus* CI]  +  **Hypothetical protein** [31%, *Comamonas testosteroni* KF–1]  +  **Type 12 methyltransferase** [46%, *Campylobacter coli* 111–3]  +  **Hypothetical protein** [28%, *Trichomonas vaginalis* G3]  +  **UDP–N–acetyl–D–mannosamine dehydrogenase** [68%, *Burkholderia ambifaria* MEX–5]  +  **UDP–N–acetylglucosamine 2–epimerase**  [71%, *Rheinheimera* *nanhaiensis* E407–8]  +  **COG1134 ABC–type polysaccharide / polyol phosphate transport system, ATPase component** [69%, *Bordetella petrii* DSM 12804]  +  **COG1682 ABC–type polysaccharide / polyol phosphate export systems, permease component**  [51%, *Bordetella petrii* DSM 12804]  +  **COG1596 Periplasmic protein involved in polysaccharide export**  [66%, *Bordetella petrii* DSM 12804]  +  **MPA2 family protein involved in capsular polysaccharide export**  [53%, *Bordetella petrii* DSM 12804] | 48  +  47  +  42  +  43  +  41  +  42  +  42  +  41  +  41  +  38  +  45  +  44  +  44  +  40  +  49  +  42 | 0.404  +  0.408  +  0.352  +  0.383  +  0.353  +  0.348  +  0.416  +  0.379  +  0.397  +  0.347  +  0.367  +  0.338  +  0.364  +  0.348  +  0.359  +  0.36 |
| 4072384–4073853  [1470] | TKWG_23620 | **Succinate semialdehyde dehydrogenase** [61%, *Chromohalobacter salexigens* DSM 3043] | 48 | 0.403 |
| 4076753–4077259  [507] | TKWG_23635 | **Hypothetical protein** [43%, *Parvibaculum lavamentivorans* DS-1] | 47 | 0.442 |
| 4111406–4111672  [267] | TKWG_23850 | **Hypothetical protein** [38%, *Chlamydophila psittaci* Cal10] | 49 | 0.320 |
| 4325136 – 4327446  [2311] | TKWG_25205 to TKWG_25215 | **tRNA uridine 5–carboxymethylaminomethyl modification enzyme GidA (COG0445 NAD/FAD–utilizing enzyme apparently involved in cell division)** [85%, *Collimonas fungivorans* Ter331]  +  **Methionine aminotransferase (COG0436 Aspartate/tyrosine/aromatic aminotransferase)**  [68%, *Bordetella avium* 197N]  +  **Hypothetical protein** [30%, *Blastopirellula marina* DSM 3645] | 50  +  46  +  46 | 0.359  +  0.444  +  0.366 |
| 4338068–4339018  [951] | TKWG_25265 | **Hypothetical protein** [86%, *Azotobacter vinelandii* DJ] | 44 | 0.519 |
| **Total HGT =** 114,843 bp | | | | |

**Table B.** G+C content and Codon Adaptation Index (CAI) of the regions in the *Achromobacter xylosoxidans* A8 genome predicted to have been acquired via HGT. The average G+C % of the genome is 66.0. The mean CAI of the genome is 0.524. NA = not applicable.

| **Approximate position**  **[Length of total HGT in bp]** | **Locus Tags** | **BLASTp hits** | **G+C %** | **MCAI** |
| --- | --- | --- | --- | --- |
| 82175 – 82465  [291] | AXYL_00079 | **Barstar family protein**  [52%, *Pseudomonas fluorescens* A506] | 51 | 0.168 |
| 84188 – 90508  [6321] | AXYL_00082  +  AXYL_00083 | **Restriction endonuclease family protein** [40%, *Chlorobium phaeobacteroides* BS1]  +  **Hypothetical protein** [37%, *Acidithiobacillus ferrivorans* SS3] | 49  +  60 | 0.134  +  0.217 |
| 388745 – 389305  [561] | AXYL_00369  +  AXYL_00370 | **Hypothetical protein** [45%, *Edwardsiella tarda* ATCC 23685]  +  **ATP synthase protein I** [95%, *Achromobacter piechaudii* ATCC 43553] [77%, *Bordetella petrii* DSM 12804] | 57  +  59 | 0.322  +  0.145 |
| 438063 – 438137  [75] | AXYL_00416 | **tRNA–Arg** | 53 | NA |
| 474806 – 475675  [870] | AXYL_00449 | **Bacterial regulatory LysR family protein** [35%, *Burkholderia vietnamiensis* G4] | 60 | 0.289 |
| 666134 – 667191  [1058] | AXYL_00622  +  AXYL_00623 | **Thioredoxin family protein**  [89%, *Achromobacter arsenitoxydans* SY8] [68%, *Bordetella pertussis* Tohama I]  +  **3–dehydroquinate dehydratase** [97%, *Achromobacter piechaudii* ATCC 43553] [87%, *Bordetella petrii* DSM 12804] | 58  +  59 | 0.254  +  0.270 |
| 728620 – 730545  [1926] | AXYL_00678 | **PrkA serine protein kinase C–terminal domain protein** [99%, *Achromobacter arsenitoxydans* SY8] [94%, *Bordetella petrii* DSM 12804] | 60 | 0.595 |
| 808933 – 809397  [465] | AXYL_00760 | **Hypothetical protein** [92%, *Achromobacter arsenitoxydans* SY8] [88%, *Bordetella petrii* DSM 12804] | 59 | 0.407 |
| 816517 – 823555  [7039] | AXYL_00769 to AXYL_00775 | **Leucine–responsive regulatory protein**  [98%, *Achromobacter piechaudii* ATCC 43553] [88%, *Bordetella pertussis* Tohama I]  +  **16S rRNA** + **two tRNAs** + **23S rRNA** + **two 5S rRNAs** | 60  +  53 | 0.305  +  NA |
| 824623 – 824901  [279] | AXYL_00777 | **SWIB/MDM2 domain protein** [100%, *Bordetella petrii* DSM 12804] | 57 | 0.549 |
| 854106 – 857651  [3546] | AXYL_00800 | **Phospholipase D active site motif protein** [49%, *Xanthomonas gardneri* ATCC 19865] | 60 | 0.260 |
| 930556 – 931904  [1349] | AXYL_00869  +  AXYL_00870 | **Integrase family protein** [69%, *Burkholderia ambifaria* MC40–6]  +  **Hypothetical protein** [68%, *Achromobacter piechaudii* ATCC 43553] [50%, *Burkholderia multivorans* ATCC 17616] | 57  +  57 | 0.329  +  0.237 |
| 1111075 – 1112079  [1005] | AXYL_01038 | **Hypothetical protein**  [41%, *Burkholderia multivorans* ATCC 17616] | 58 | 0.275 |
| 1115176 – 1115703  [528] | AXYL_01041 | **Hypothetical protein** [57%, *Burkholderia ambifaria* MEX–5] | 57 | 0.432 |
| 1187479 – 1188915  [1437] | AXYL_01120 | **Sensor kinase CusS** [98%, *Achromobacter piechaudii* ATCC 43553] [98%, Pseudomonas aeruginosa DK2] | 56 | 0.202 |
| 1197754 – 1198038  [285] | AXYL_01130 | **Hypothetical protein** [98%, *Stenotrophomonas maltophilia* D457] | 59 | 0.271 |
| 1199165 – 1200297  [1133] | AXYL_01132 to AXYL_01134 | **Helix–turn–helix family protein** [98%, *Pseudomonas aeruginosa* 152504]  +  **LysR family regulatory helix–turn–helix protein** [98%, *Pseudomonas aeruginosa* 152504]  +  **Hypothetical protein** [96%, *Pseudomonas aeruginosa* 39016] | 60  +  55  +  60 | 0.138  +  0.146  +  0.278 |
| 1251446 – 1252705  [1260] | AXYL_01185 | **O–antigen polymerase family protein** [75%, *Achromobacter piechaudii* ATCC 43553] [55%, *Bordetella petrii* DSM 12804] | 59 | 0.273 |
| 1314402 – 1314671  [270] | AXYL_01243 | **Ribosomal protein S15 (rpsO)** [99%, *Bordetella petrii* DSM 12804] | 57 | 0.389 |
| 1541925 – 1542284  [360] | AXYL_01458 | **NADH–quinone oxidoreductase subunit A** [99%, *Achromobacter piechaudii* ATCC 43553] [97%, *Bordetella avium* 197N] | 58 | 0.418 |
| 1906931 – 1907479  [549] | AXYL_01790 | **Hypothetical protein** [93%, *Achromobacter arsenitoxydans* SY8] [86%, *Bordetella pertussis* Tohama I] | 57 | 0.342 |
| 1949285 – 1951176  [1892] | AXYL_01826  +  AXYL_01827 | **Glyoxalase/bleomycin resistance protein / dioxygenase superfamily protein** [54%, *Thermomonospora curvata* DSM 43183]  +  **LysR family regulatory protein** [47%, *Sphingomonas wittichii* RW1] | 58  +  60 | 0.316  +  0.255 |
| 1987670 – 1988344  [675] | AXYL_01870 | **Putative lipoprotein** [50%, *Achromobacter arsenitoxydans* SY8] [34%, *Desulfovibrio salexigens* DSM 2638] | 57 | 0.361 |
| 2059730 – 2060609  [880] | AXYL_01940  +  AXYL_01941 | **Methyltransferase domain protein** [52%, *Bordetella bronchiseptica* RB50]  +  **tRNA–Met** | 59  +  58 | 0.350  +  NA |
| 2079326 – 2079598  [273] | AXYL_01959 | **DNA–binding protein HU–beta** [100%, *Bordetella pertussis* Tohama I] | 60 | 0.462 |
| 2153368 – 2153958  [591] | AXYL_02024 | **Tripartite ATP–independent periplasmic transporter, DctQ component family protein** [51%, *Pelagibacterium halotolerans* B2] | 56 | 0.259 |
| 2176331 – 2177149  [819] | AXYL_02061 | **Hypothetical protein**  [42%, *Pectobacterium atrosepticum* SCRI1043] | 53 | 0.180 |
| 2200780 – 2201217  [438] | AXYL_02093 | **Hypothetical protein** [63%, *Bordetella pertussis* Tohama I] | 55 | 0.131 |
| 2206254 – 2211587  [5334] | AXYL_02096 to AXYL_02100 | **16S rRNA** + **two tRNAs** + **23S rRNA** + **5S rRNA** | 53 | NA |
| 2309219 – 2310366  [1148] | AXYL_02185  +  AXYL_02186 | **Phosphoribosyl transferase domain protein** [97%, *Achromobacter piechaudii* ATCC 43553] [88%, *Bordetella avium* 197N]  +  **rpsU / ribosomal protein S21** [100%, *Bordetella parapertussis*] | 59  +  60 | 0.487  +  0.472 |
| 2357562 – 2361752  [4191] | AXYL_02227 | **Hemolysin–type calcium–binding repeat family protein** [56%, *Bordetella bronchiseptica* RB50] | 60 | 0.326 |
| 2384362 – 2386455  [2094] | AXYL_02242  +  AXYL_02243 | **tRNA–Val**  +  **Diguanylate cyclase, GGDEF domain protein** [72%, *Achromobacter piechaudii* ATCC 43553] [73%, *Bordetella parapertussis* 12822] | 56  +  59 | NA  +  0.234 |
| 2629584 – 2631191  [1608] | AXYL_02483  +  AXYL_02484 | **Helix–turn–helix family protein** [75%, *Verminephrobacter eiseniae* EF01–2]  +  **HipA N–terminal domain protein** [70%, *Achromobacter piechaudii* ATCC 43553] [64%, *Advenella kashmirensis* WT001] | 58  +  59 | 0.224  +  0.216 |
| 2698078 – 2707220  [4143] | AXYL_02545 to AXYL_02552 | **Flavin reductase–like domain protein**  [83%, *Pseudomonas putida* ND6]  +  **5–methyltetrahydropteroyltriglutamate–homocysteine S–methyltransferase**  [95%, *Tetrathiobacter kashmirensis* WT001]  +  **Hypothetical protein** [87%, *Pseudomonas fluorescens* Pf–5]  +  **HTH–type transcriptional regulator MetR** [89%, *Herbaspirillum seropedicae* SmR1]  +  **Superfamily I DNA and RNA helicases and helicase subunits–like protein** [59%, *Polaromonas naphthalenivorans* CJ2]  +  **Hypothetical protein** [54%, *Polaromonas naphthalenivorans* CJ2]  +  **Hypothetical protein** [32%, *Methylomicrobium album* BG8]  +  **Hypothetical protein** [51%, *Methylomicrobium album* BG8] | 58  +  57  +  53  +  61  +  57  +  55  +  55  +  58 | 0.299  +  0.400  +  0.327  +  0.426  +  0.354  +  0.266  +  0.261  +  0.294 |
| 2748935 – 2749648  [714] | AXYL_02587 | **LuxR family regulatory protein** [56%, *Burkholderia ubonensis* Bu] | 57 | 0.217 |
| 2767986 – 2776100  [8115] | AXYL_02598  +  AXYL_02599 | **Syringomycin synthetase** [68%, *Variovorax paradoxus* EPS]  +  **Hypothetical protein** [70%, *Achromobacter arsenitoxydans* SY8] [63%, *Rubrivivax gelatinosus* IL144] | 60  +  52 | 0.222  +  0.403 |
| 2807260 – 2808099  [840] | AXYL_02626 | **Integrase family protein** [69%, *Burkholderia ambifaria* MC40–6] | 57 | 0.329 |
| 2926006 – 2926329  [324] | AXYL_02731 | **Flagellar transcriptional activator FlhD family protein** [99%, *Achromobacter piechaudii* ATCC 43553] [93%, *Bordetella parapertussis* 12822] | 57 | 0.350 |
| 2981058 – 2982443  [1386] | AXYL_02785 | **Flagellar hook–associated protein** [62%, *Achromobacter arsenitoxydans* SY8] [50%, *Bordetella petrii* DSM 12804] | 58 | 0.315 |
| 3018898 – 3020001  [1103] | AXYL_02821 | **Hypothetical protein** [59%, *Desulfomonile tiedjei* DSM 6799] | 55 | 0.246 |
| 3201407 – 3202664  [1258] | AXYL_02990  +  AXYL_02991 | **Hypothetical protein** [49%, *Burkholderia ambifaria* IOP40–10]  +  **Hypothetical protein** [64%, *Achromobacter piechaudii* ATCC 43553] [58%, *Burkholderia pseudomallei* K96243] | 57  +  57 | 0.302  +  0.229 |
| 3214328 – 3215252  [925] | AXYL_03006  +  AXYL_03007 | **Acetyltransferase, GNAT family protein**  [63%, *Pseudomonas putida* GB–1]  +  **Glyoxalase/bleomycin resistance protein/dioxygenase superfamily protein** [73%, *Pseudomonas entomophila* L48] | 57  +  57 | 0.244  +  0.301 |
| 3303314 – 3304471  [1158] | AXYL_03085 | **Fic family protein** [50%, *Variovorax paradoxus* S110] | 60 | 0.249 |
| 3436646 – 3439198  [2553] | AXYL_03198  +  AXYL_03199 | **Major facilitator superfamily protein**  [48%, *Pseudomonas mendocina* ymp]  +  **LysR family regulatory protein** [42%, *Pseudomonas mendocina* ymp] | 60  +  57 | 0.273  +  0.178 |
| 3616286 – 3616603  [318] | AXYL_03356 | **Hypothetical protein** [55%, *Acidithiobacillus thiooxidans* ATCC 19377] | 53 | 0.284 |
| 3745003 – 3746553  [1551] | AXYL_03479 | **Putative membrane protein** [34%, *Microbacterium testaceum* StLB037] | 57 | 0.180 |
| 3814258 – 3823959  [9702] | AXYL_03547 to AXYL_03557 | **Protein involved in meta-pathway of phenol degradation** [35%, *Pseudomonas putida* KT2440]  +  **Hypothetical protein** [31%, *Mesorhizobium amorphae* CCNWGS0123]  +  **Transposase, IS3 family protein** [71%, *Herminiimonas arsenicoxydans*]  +  **Hypothetical protein** [52%, *Variovorax paradoxus* EPS]  +  **Cysteinyl-tRNA synthetase** [81%, *Burkholderia terrae* BS001]  +  **Putative membrane protein** [91%, *Achromobacter arsenitoxydans* SY8] [58%, *Cupriavidus metallidurans* CH34]  +  **Ribosomal RNA large subunit methyltransferase N** [89*%, Bordetella avium* 197N]  +  **D-alanyl-D-alanine carboxypeptidase family protein** [45%, *Myxococcus xanthus* DK 1622]  +  **Hypothetical protein** [24%, *Hyphomicrobium denitrificans* 1NES1]  +  **Hypothetical protein** [47%, *Bordetella bronchiseptica* RB50]  +  **Hypothetical protein** [82%, *Ralstonia eutropha* H16] | 59  +  54  +  50  +  55  +  57  +  63  +  65  +  53  +  53  +  61  +  53 | 0.309  +  0.189  +  0.189  +  0.175  +  0.239  +  0.271  +  0.419  +  0.183  +  0.202  +  0.274  +  0.181 |
| 3861372 – 3863700  [2329] | AXYL_03597 to AXYL_03599 | **Hypothetical protein** [47%, *Methylocella silvestris* BL2]  +  **OmpA family protein** [44%, *Methylococcus capsulatus* str. Bath]  +  **AraC family regulatory helix-turn-helix protein** [29%, *Methylobacterium chloromethanicum* CM4] | 53  +  57  +  61 | 0.145  +  0.245  +  0.279 |
| 3864481 – 3865923  [1443] | AXYL_03601 | **Hypothetical protein** [84%, *Sinorhizobium meliloti* CCNWSX0020] | 60 | 0.363 |
| 4037872 – 4052316  [14445] | AXYL_03763 to AXYL_03778 | **Hypothetical protein** [97%, *Achromobacter arsenitoxydans* SY8] [85%, *Burkholderia graminis* C4D1M]  +  **Hypothetical protein** [51%, *Cupriavidus basilensis* OR16]  +  **Hypothetical protein** [47*%, Bordetella parapertussis* 12822]  +  **Hypothetical protein** [49%, *Novosphingobium nitrogenifigens* DSM 19370]  +  **Hypothetical protein** [55%, *Gallionella capsiferriformans* ES-2]  +  **Hypothetical protein** [39%, *Leptonema illini* DSM 21528]  +  **Hypothetical protein** [33%, *Escherichia coli* 96.154]  +  **Hypothetical protein** [35%, *Methylobacterium extorquens* DM4]  +  **Hypothetical protein** [19%, *Shewanella baltica* OS185]  +  **Hypothetical protein** [31%, *Xanthomonas campestris* pv. vesicatoria str. 85-10]  +  **Putative membrane protein** [24%, *Vibrio rotiferianus* DAT722]  +  **Hypothetical protein** [47%, *Pseudomonas fluorescens* F113]  +  **Transposase, IS3/IS911 family protein** [54%, *Rhodanobacter fulvus* Jip2]  +  **Integrase family protein** [53%, *Rhodanobacter fulvus* Jip2]  +  **Hypothetical protein** [80%, *Achromobacter arsenitoxydans* SY8] [79%, *Advenella kashmirensis* WT001]  +  **Hypothetical protein** [51%, *Herbaspirillum seropedicae* SmR1] | 60  +  62  +  55  +  47  +  51  +  49  +  53  +  53  +  39  +  53  +  52  +  57  +  56  +  60  +  60  +  60 | 0.455  +  0.313  +  0.287  +  0.162  +  0.141  +  0.120  +  0.201  +  0.124  +  0.086  +  0.184  +  0.144  +  0.156  +  0.275  +  0.262  +  0.227  +  0.233 |
| 4071668 – 4074008  [2341] | AXYL_03797 to AXYL_03800 | **HD domain protein** [83%, *Comamonas testosteroni* KF-1]  +  **Hypothetical protein** [52%, *Achromobacter arsenitoxydans* SY8] [39%, *Pantoea ananatis* PA13]  +  **Hypothetical protein** [60%, *Achromobacter piechaudii* ATCC 43553] [55%, *Alcaligenes faecalis* subsp. faecalis NCIB 8687]  +  **Hypothetical protein** [62%, *Pseudomonas aeruginosa* 138244] | 58  +  55  +  62  +  57 | 0.384  +  0.321  +  0.462  +  0.304 |
| 4197668 – 4200894  [3227] | AXYL_03898 to AXYL_03901 | **AMP-binding enzyme family protein** [49%, *Streptosporangium roseum* DSM 43021]  +  **MaoC like domain protein** [60%, *Cupriavidus metallidurans* CH34]  +  **Acyl dehydratase** [59%, *Cupriavidus metallidurans* CH34]  +  **Hypothetical protein** [56%, *Sphingobium chlorophenolicum* L-1] | 59  +  56  +  56  +  56 | 0.278  +  0.259  +  0.240  +  0.302 |
| 4204157 – 4207582  [3426] | AXYL_03905 to AXYL_03907 | **Amidohydrolase domain protein** [44%, *Comamonas testosteroni* ATCC 11996]  +  **Hypothetical protein** [28%, *Rhodospirillum centenum* SW]  +  **Hypothetical protein** [35%, *Asticcacaulis biprosthecum* C19] | 59  +  57  +  59 | 0.194  +  0.218  +  0.254 |
| 4281467 – 4282366  [900] | AXYL_03977  +  AXYL_03978 | **Cold-shock DNA-binding domain protein** [100%, *Achromobacter piechaudii* ATCC 43553] [97%, *Bordetella petrii* DSM 12804]  +  **Hypothetical protein** [95%, *Achromobacter arsenitoxydans* SY8] [73%, *Bordetella petrii* DSM 12804] | 56  +  56 | 0.565  +  0.263 |
| 4298710 – 4299465  [756] | AXYL_04001 | **Hypothetical protein** [27%, *Advenella kashmirensis* WT001] | 57 | 0.158 |
| 4300875 – 4302577  [1703] | AXYL_04005 to AXYL_04007 | **Hypothetical protein** [48%, *Achromobacter piechaudii* ATCC 43553] [35%, *Streptomyces sviceus* ATCC 29083]  +  **Hypothetical protein** [33%, *Helicobacter cetorum* MIT 00-7128]  +  **Hypothetical protein** [71%, *Bordetella petrii* DSM 12804] | 55  +  55  +  56 | 0.195  +  0.145  +  0.215 |
| 4304907 – 4315522  [10616] | AXYL_04011 to AXYL_04019 | **Hypothetical protein** [51%, *Klebsiella pneumoniae subsp. rhinoscleromatis* ATCC 13884]  +  **Hypothetical protein** [33%, *Acinetobacter baumannii* AB900]  +  **Hypothetical protein** [30%, *Acinetobacter baumannii* AB900]  +  **Hypothetical protein** [34%, *Acinetobacter baumannii* ATCC 19606]  +  **Hypothetical protein** [36%, *Acinetobacter baumannii* AB058]  +  **Phage tail tape measure protein, lambda family** [37%, *Bordetella pertussis Tohama* I]  +  **Hypothetical protein** [47%, *Stenotrophomonas maltophilia* JV3]  +  **Hypothetical protein** [36%, *Stenotrophomonas maltophilia* D457]  +  **Phage tail family protein** [45%, *Methyloversatilis universalis* FAM5] | 60  +  58  +  56  +  56  +  57  +  60  +  59  +  57  +  58 | 0.246  +  0.223  +  0.168  +  0.156  +  0.194  +  0.208  +  0.174  +  0.408  +  0.363 |
| 4320914 – 4323086  [2173] | AXYL_04028  +  AXYL_04029 | **Phage terminase family protein** [70%, *Burkholderia thailandensis* E264]  +  **Hypothetical protein** [47%, *Pseudomonas psychrotolerans* L19] | 59  +  60 | 0.304  +  0.278 |
| 4324237 – 4326270  [2034] | AXYL_04032 to AXYL_04034 | **Hypothetical protein** [62%, *Achromobacter arsenitoxydans* SY8] [51%, *Burkholderia terrae* BS001]  +  **Hypothetical protein** [32%, *Clostridium saccharolyticum* WM1]  +  **Hypothetical protein** [38%, *Pseudomonas psychrotolerans* L19] | 59  +  55  +  50 | 0.287  +  0.158  +  0.191 |
| 4330851 – 4334349  [3499] | AXYL_04043 to AXYL_04049 | **Hypothetical protein** [42%, *Burkholderia pseudomallei* MSHR346]  +  **Repressor protein C2 domain protein** [58%, *Bordetella bronchiseptica* RB50]  +  **Hypothetical protein** [26%, *Shewanella halifaxensis* HAW-EB4]  +  **Hypothetical protein** [42%, *Vibrio shilonii* AK1]  +  **Hypothetical** protein [82%, *Achromobacter arsenitoxydans* SY8] [43%, *Desulfobacter postgatei* 2ac9**]**  +  **Hypothetical protein** [45%, *Methylibium petroleiphilum* PM1]  +  **Cold shock protein CapA** [94%, *Achromobacter piechaudii* ATCC 43553] [83%, *Alcaligenes faecalis subsp. faecalis* NCIB 8687] | 56  +  57  +  54  +  66  +  60  +  61  +  50 | 0.393  +  0.200  +  0.216  +  0.412  +  0.327  +  0.389  +  0.287 |
| 4334936 – 4335565  [630] | AXYL_04052 | **Hypothetical protein** [42%, *Burkholderia pseudomallei* MSHR346] | 52 | 0.142 |
| 4340311 – 4340853  [543] | AXYL_04063 | **Hypothetical protein** [41%, *Vibrio cholerae* HE-09] | 55 | 0.178 |
| 4424768 – 4425553  [786] | AXYL_04133  +  AXYL_04134 | **ATP-dependent Clp protease adaptor protein** [99%, *Achromobacter piechaudii* ATCC 43553] [92%, *Bordetella petrii* DSM 12804]  +  **ClpS + cold shock-like protein CspG** [100%, *Achromobacter piechaudii* ATCC 43553] [99%, *Bordetella petrii* DSM 12804] | 60  +  52 | 0.361  +  0.271 |
| 4538715 – 4545927  [7213] | AXYL_04254 to AXYL_04262 | **Hypothetical protein** [52%, *Bordetella bronchiseptica* RB50]  +  **Hypothetical protein** [54%, *Bordetella bronchiseptica* RB50]  +  **Putative lipoprotein 11** [70%, *Achromobacter arsenitoxydans* SY8] [33%, *Variovorax paradoxus* S110]  +  **Putative membrane protein 54** [27%, *Lentisphaera araneosa* HTCC2155]  +  **Hypothetical protein** [28%, *Burkholderia ambifaria* MEX-5]  +  **Putative lipoprotein** [33%, *Rhodothermus marinus* DSM 4252]  +  **Integrase family protein** [56%, *Sideroxydans lithotrophicus* ES-1]  +  **Transposase, IS3/IS911 family protein** [57%, *Sideroxydans lithotrophicus* ES-1]  +  **Putative membrane protein** [38%, Burkholderia multivorans ATCC 17616] | 57  +  56  +  57  +  63  +  52  +  56  +  60  +  56  +  50 | 0.249  +  0.263  +  0.307  +  0.343  +  0.156  +  0.236  +  0.262  +  0.275  +  0.141 |
| 4605201 – 4605805  [605] | AXYL_04305  +  AXYL_04306 | **Prevent-host-death family protein** [95%, *Achromobacter arsenitoxydans* SY8] [68%, *Pseudomonas fluorescens* Q2-87]  +  **Plasmid stabilization system family protein** [76%, *Achromobacter arsenitoxydans* SY8] [45%, *Methylobacter tundripaludum* SV96] | 52  +  52 | 0.329  +  0.265 |
| 4799228 – 4800277  [1050] | AXYL_04478 | **Polysaccharide export outer membrane protein EpsA** [89%, *Achromobacter arsenitoxydans* SY8] [53%, *Cupriavidus necator* N-1] | 59 | 0.312 |
| 4816374 – 4817978  [1605] | AXYL_04494 | **Hypothetical protein** [89%, *Achromobacter arsenitoxydans* SY8] [31%, *Maritimibacter alkaliphilus* HTCC2654] | 59 | 0.331 |
| 4881898 – 4882737  [840] | AXYL_04555 | **Integrase family protein** [69%, *Burkholderia ambifaria* MC40-6] | 57 | 0.329 |
| 4959830 – 4964159  [4330] | AXYL_04645 to AXYL_04652 | **Hypothetical protein** [61%, *Burkholderia graminis* C4D1M]  +  **Hypothetical protein** [75%, *Achromobacter arsenitoxydans* SY8] [41%, *Photorhabdus asymbiotica subsp. asymbiotica* ATCC 43949]  +  **Hypothetical protein** [48%, *Cupriavidus basilensis* OR16]  +  **Hypothetical protein** [30%, *Burkholderia pseudomallei* 112]  +  **Hypothetical protein** [32%, *Bacillus cereus* AH621]  +  **Hypothetical protein** [44%, *Yersinia aldovae* ATCC 35236]  +  **Transposase, IS3 family protein** [95%, *Bordetella pertussis* Tohama I]  +  **Integrase core domain protein** [79%, *Comamonas testosteroni* KF-1] | 59  +  54  +  53  +  54  +  49  +  56  +  62  +  58 | 0.386  +  0.185  +  0.154  +  0.192  +  0.352  +  0.202  +  0.454  +  0.280 |
| 5038198 – 5038834  [14207] | AXYL_04719  +  AXYL_04720 | **YcfA family protein** [97%, *Bordetella pertussis* Tohama I]  +  **hypothetical protein** [98%, *Bordetella pertussis* Tohama I] | 54  +  57 | 0.184  +  0.298 |
| 5052078 – 5052404  [327] | AXYL_04736 | **Bacterial regulatory protein, ArsR family protein** [83%, *Bordetella avium* 197N] | 61 | 0.244 |
| 5055719 – 5061463  [5745] | AXYL_04741 to AXYL_04744 | **Arsenate reductase (glutaredoxin)** [86%, *Ralstonia pickettii* 12D]  +  **Arsenical resistance protein ArsH 2** [88%, *Bordetella bronchiseptica* RB50]  +  **SEC-C motif family protein** [61%, *Burkholderia pseudomallei* 305]  +  **Hypothetical protein** [50%, *Vibrio mimicus* VM603] | 57  +  61  +  58  +  52 | 0.323  +  0.298  +  0.243  +  0.153 |
| 5071457 – 5072721  [1265] | AXYL_04753  +  AXYL_04754 | **DJ-1/PfpI family protein** [80%, *Pseudomonas brassicacearum subsp. brassicacearum* NFM421]  +  **Bacterial regulatory protein, TetR family protein** [74%, *Pseudomonas fluorescens* Q8r1-96] | 58  +  59 | 0.182  +  0.183 |
| 5073896 – 5074186  [291] | AXYL_04756 | **Hypothetical protein** [90%, *Achromobacter arsenitoxydans* SY8] [*Methylobacterium extorquens* AM1] | 60 | 0.447 |
| 5156381 – 5161714  [5334] | AXYL_04831  to  AXYL_04835 | **5S rRNA** +**23S rRNA + two tRNAs + 16S rRNA** | 53 | NA |
| 5293356 – 5293691  [336] | AXYL_04932 | **Hypothetical protein** [41%, *Halomonas boliviensis* LC1] | 59 | 0.439 |
| 5346955 – 5347653  [699] | AXYL_04982 | **Transcription elongation factor GreA** [97%, *Achromobacter arsenitoxydans* SY8] [93%, *Bordetella pertussis* Tohama I] | 61 | 0.302 |
| 5399348 – 5399767  [420] | AXYL_05026 | **Hypothetical protein** [82%, *Pseudomonas syringae pv. lachrymans* str. M302278] | 58 | 0.244 |
| 5529695 – 5530522  [828] | AXYL_05155 | **Integrase family protein** [69%, *Burkholderia ambifaria* MC40-6] | 57 | 0.326 |
| 5541502 – 5542126  [625] | AXYL_05167 to AXYL_05171 | **Five tRNAs** | 57 | NA |
| 5902160 – 5902852  [693] | AXYL_05512 | **Bacterial GntR family regulatory protein** [40%, *Pseudomonas aeruginosa* PAO1] | 60 | 0.238 |
| 5905688 – 5907118  [1431] | AXYL_05515 | **Aminotransferase class-V family protein** [47%, *Phenylobacterium zucineum* HLK1] | 58 | 0.297 |
| 5921695 – 5924299  [2605] | AXYL_05531  +  AXYL_05532 | **Major facilitator superfamily protein** [57%, *Pseudomonas syringae pv. actinidiae* str. M302091]  +  **Helix-turn-helix regulatory protein** [72%, *Photorhabdus luminescens subsp. laumondii* TTO1] | 59  +  59 | 0.179  +  0.213 |
| 5928176 – 5929714  [1539] | AXYL_05537  +  AXYL_05538 | **Helix-turn-helix AraC family regulatory**  **protein** [58%, *Ochrobactrum intermedium* LMG 3301]  +  **Acetyltransferase, GNAT family protein** [72%, *Ochrobactrum intermedium* LMG 3301] | 57  +  59 | 0.339  +  0.420 |
| 6128811 – 6131959  [3149] | AXYL_05705 to AXYL_05707 | **Putative membrane protein** [30%, *Pseudomonas syringae pv. oryzae str*. 1_6]  +  **Putative membrane protein**[25%, *Filifactor alocis* ATCC 35896]  +  **Phospholipase D active site motif protein** [28%, *Ralstonia solanacearum* CFBP2957] | 53  +  58  +  59 | 0.179  +  0.247  +  0.304 |
| 6169312 – 6171504  [2193] | AXYL_05735 | **Peptidase family M48 family protein** [28%, *Burkholderia pseudomallei* 1655] | 51 | 0.115 |
| 6252129 – 6253112  [984] | AXYL_05808 | **Extra–cytoplasmic solute receptor family protein** [88%, *Bordetella petrii DSM* 12804] | 58 | 0.310 |
| 6289208 – 6289837  [630] | AXYL_05845 | **HAD–superfamily hydrolase, subfamily IA, variant 1 family protein** [60%, *Achromobacter arsenitoxydans* SY8] [46%, *Pseudomonas stutzeri* A1501] | 57 | 0.222 |
| 6292841 – 6293680  [840] | AXYL_05849 | **Hypothetical protein** [66%, *Burkholderia ubonensis* Bu] | 54 | 0.233 |
| 6430261 – 6431190  [930] | AXYL_05972 | **Abi-like family protein** [39%, *Xanthomonas perforans* 91-118] | 56 | 0.183 |
| 6484065 – 6484694  [630] | AXYL_06024 | **LuxR family regulatory protein** [58%, *Bordetella petrii* DSM 12804] | 54 | 0.281 |
| 6492771 – 6493781  [1011] | AXYL_06030 | **Fimbrial family protein** [37%, *Collimonas fungivorans* Ter331] | 56 | 0.300 |
| 6523535 – 6523846  [312] | AXYL_06052 | **RplU / ribosomal protein L21** [100%, *Achromobacter piechaudii* ATCC 43553] [97%, *Bordetella avium* 197N] | 57 | 0.395 |
| 6528368 – 6529614  [1247] | AXYL_06059  +  AXYL_06060 | **Helix-turn-helix family protein** [63%, *Bordetella parapertussis* 12822]  +  **Integrase family protein** [72, *Alcaligenes faecalis subsp. faecalis* NCIB 8687] | 60  +  57 | 0.247  +  0.329 |
| 6596383 – 6597731  [1349] | AXYL_06125  +  AXYL_06126 | **Hypothetical protein** [68%, *Achromobacter piechaudii* ATCC 43553] [50%, *Burkholderia multivorans* ATCC 17616]  +  **Integrase family protein** [72%, *Alcaligenes faecalis subsp. faecalis* NCIB 8687] | 57  +  57 | 0.237  +  0.329 |
| 6612616 – 6612954  [339] | AXYL_06142 | **Ribosomal subunit interface protein raiA** [97%, *Achromobacter piechaudii* ATCC 43553] [94%, *Bordetella petrii* DSM 12804] | 55 | 0.254 |
| 6750401 – 6761834  [11434] | AXYL_06282 to AXYL_06291 | **Hypothetical protein** [26%, *Collimonas fungivorans* Ter331]  +  **Putative membrane protein** [38%, *Pseudomonas aeruginosa*]  +  **Bacterial transferase hexapeptide family protein** [39%, *Xenorhabdus nematophila* ATCC 19061]  +  **N-acetyl glucosamine deacetylase family protein** [51%, *Ferroglobus placidus* DSM 10642, an archaeon]  +  **NAD-binding Rossmann fold family oxidoreductase protein** [81%, *Rhodanobacter thiooxydans* LCS2]  +  **Bacterial transferase hexapeptide family protein** [81%, *Roseovarius nubinhibens* ISM]  +  **Pleiotropic regulatory protein** [78%, *Acinetobacter calcoaceticus* PHEA-2]  +  **Hypothetical protein** [42%, *Variovorax paradoxus* S110]  +  **Glycosyl transferase, group 1 family protein** [42%, *Hoeflea phototrophica* DFL-43]  +  **LPS biosynthesis protein WbpG** [72%, *Pseudomonas aeruginosa* PA7] | 56  +  57  +  61  +  56  +  55  +  60  +  61  +  47  +  44  +  57 | 0.246  +  0.280  +  0.262  +  0.336  +  0.313  +  0.420  +  0.402  +  0.082  +  0.126  +  0.482 |
| 6772372 – 6773436  [1065] | AXYL_06302 | **dTDP-glucose 4,6-dehydratase** [75%, *Pseudomonas aeruginosa* PAO1] | 57 | 0.287 |
| 6775646 – 6785520  [9875] | AXYL_06305 to AXYL_06314 | **Hypothetical protein** [81%, *Achromobacter arsenitoxydans* SY8] [76%, *Bordetella parapertussis* 12822]  +  **Glucose-1-phosphate thymidylyltransferase** [70%, *Rhizobium leguminosarum bv. viciae* 3841]  +  **Putative membrane protein** [39%, *Pseudomonas syringae pv. tabaci* str. ATCC 11528]  +  **Small multidrug resistance family protein** [28%, *Planctomyces maris* DSM 8797]  +  **Methyltransferase domain protein** [53%, *Desulfarculus baarsii* DSM 2075]  +  **Methyltransferase domain protein** [54%, *Desulfarculus baarsii* DSM 2075]  +  **NAD dependent epimerase/dehydratase family** [46%, *Niastella koreensis* GR20-10]  +  **Transketolase, pyrimidine binding domain** [59%, *Desulfarculus baarsii* DSM 2075]  +  **Transketolase** [65%, *Desulfarculus baarsii* DSM 2075]  +  **Putative membrane protein** [37%, *Pseudomonas brassicacearum subsp. brassicacearum* NFM421] | 59  +  52  +  54  +  54  +  53  +  54  +  52  +  59  +  57  +  58 | 0.168  +  0.162  +  0.175  +  0.208  +  0.163  +  0.137  +  0.157  +  0.186  +  0.189  +  0.173 |
| 6820902 – 6837007  [16106] | AXYL_06349 to AXYL_06363 | **Type III restriction enzyme, Res subunit** [69%, *Rhodanobacter thiooxydans* LCS2]  +  **Type I restriction enzyme StySPI specificity** [46%, *Alishewanella agri* BL06]  +  **N-6 adenine-specific DNA methylase** [73%, *Acinetobacter lwoffii* SH145]  +  **Hypothetical protein** [37%, *Ralstonia eutropha* JMP134]  +  **Hypothetical protein** [40%, *Desulfatibacillum alkenivorans* AK-01]  +  **Hypothetical protein** [41%, *Methylosinus trichosporium* OB3b]  +  **Hypothetical protein** [37%, *Pseudomonas syringae pv. tomato* T1]  +  **Hypothetical protein** [43%, *Pseudomonas syringae pv. tomato* Max13]  +  **Papain family cysteine protease** [38%, *Sphingobium japonicum* UT26S]  +  **Hypothetical protein** [44%, *Pseudomonas syringae pv. tomato* Max13]  +  **Hypothetical protein** [65%, *Pseudomonas syringae pv. tomato* T1]  +  **MazG nucleotide pyrophosphohydrolase** [49%, *Pseudomonas syringae pv. tomato* NCPPB 1108]  +  **Hypothetical protein** [70%, *Acidithiobacillus ferrooxidans* ATCC 23270]  +  **Hypothetical protein** [38%, *Clostridium perfringens*; 38%, envelope protein of Feline leukemia virus]  +  **Hypothetical protein** [39%, *Pseudomonas putida* GB-1] | 58  +  50  +  58  +  55  +  57  +  52  +  53  +  54  +  55  +  54  +  53  +  53  +  52  +  48  +  60 | 0.326  +  0.141  +  0.368  +  0.180  +  0.146  +  0.115  +  0.214  +  0.159  +  0.169  +  0.144  +  0.126  +  0.189  +  0.150  +  0.342  +  0.242 |
| 6840529 – 6841785  [1257] | AXYL_06366 | **Hypothetical protein** [35%, *Comamonas testosteroni* S44] | 57 | 0.181 |
| 6908658 – 6910767  [2110] | AXYL_06424 to AXYL_06427 | **30S ribosomal protein S13 / rpsM** [100%, *Achromobacter piechaudii* ATCC 43553] [96%, *Bordetella petrii* DSM 12804]  +  **50S ribosomal protein L36 / rpmJ** [100%, *Bordetella pertussis* Tohama I]  +  **Translation initiation factor IF-1 2** [100%, *Bordetella pertussis* Tohama I]  +  **Preprotein translocase, SecY subunit** [99%, *Achromobacter piechaudii* ATCC 43553] [97%, *Bordetella bronchiseptica* RB50] | 59  +  54  +  54  +  59 | 0.314  +  0.312  +  0.284  +  0.387 |
| 6914167 – 6914487  [321] | AXYL_06436 | **RplX ribosomal protein L24** [98%, *Achromobacter piechaudii* ATCC 43553] [97%, *Bordetella pertussis* Tohama I] | 61 | 0.524 |
| 6914499 – 6914867  [369] | AXYL_06437 | **Ribosomal protein L14 / rplN** [100%, *Achromobacter piechaudii* ATCC 43553] [99%, *Bordetella avium* 197N] | 58 | 0.394 |
| 6925428 – 6925619  [192] | AXYL_06449 | **RpmC ribosomal protein L29** [100%, *Bordetella pertussis* Tohama I] | 58 | 0.348 |
| 6934771 – 6935148  [378] | AXYL_06462 | **Ribosomal protein S12 / rpsL** [100%, *Bordetella pertussis* Tohama I] | 60 | 0.486 |
| 6954327 – 6954707  [381] | AXYL_06476 | **SecE preprotein translocase, SecE subunit** [99%, *Achromobacter arsenitoxydans* SY8] | 60 | 0.384 |
| 6956566 – 6956652  [87] | AXYL_06481 | **tRNA–Tyr** | 56 | NA |
| 6980671 – 6980877  [207] | AXYL_06500 | **Cold shock protein CspB** [100%, *Achromobacter piechaudii* ATCC 43553] [99%, *Bordetella parapertussis* 12822] | 55 | 0.353 |
| 6988312 – 6988989  [678] | AXYL_06507 | **Transcriptional regulatory protein TctD 4** [95%, *Achromobacter arsenitoxydans* SY8] [87%, *Bordetella parapertussis* 12822] | 57 | 0.247 |
| 6990903 – 6991355  [453] | AXYL_06509 | **Acetyltransferase, GNAT family protein** [71%, *Achromobacter arsenitoxydans* SY8] [60%, *Azorhizobium caulinodans* ORS 571] | 61 | o.311 |
| **Total HGT =** 239,444 bp | | | | |

**Table C.** G+C content and Codon Adaptation Index (CAI) of the regions in the *Bordetella bronchiseptica* RB50 genome predicted to have been acquired via HGT. The average G+C % of the genome is 68.1. The mean CAI of the genome is 0.580. NA = not applicable.

| **Approximate position**  **[Length of total HGT in bp]** | **Locus Tags** | **BLASTp hits** | **G+C %** | **CAI** |
| --- | --- | --- | --- | --- |
| 8136 – 9059  [924] | BB0008  +  BB0009 | **Preprotein translocase subunit SecE** [100*%, Bordetella pertussis* Tohama I; 90%, *Achromobacter xylosoxidans* A8]  +  **Transcription antitermination protein NusG** [100%, *Bordetella pertussis* Tohama I; 98%, *Achromobacter xylosoxidans* A8] | 59  +  54 | 0.304  +  0.395 |
| 29103 – 29480  [378] | BB0024 | **30S ribosomal protein S12 (rpsL)** [100%, *Bordetella pertussis* Tohama I; 93%, *Taylorella equigenitalis* MCE9] | 61 | 0.431 |
| 43147 – 43686  [540] | BB0043 | **50S ribosomal protein L5 (rplE)**  [100%, *Bordetella pertussis* Tohama I; 96%, *Achromobacter xylosoxidans* C54] | 58 | 0.508 |
| 46532 – 48640  [2109] | BB0051  +  BB0052  +  BB0053  +  BB0054 | **Preprotein translocase subunit SecY** [99%, *Bordetella pertussis* Tohama I; 97%, *Achromobacter xylosoxidans* A8]  +  **InfA** [100%, *Bordetella pertussis* Tohama I; 97%, *Leptothrix cholodnii* SP-6]  +  **RpmJ** [100%, *Bordetella pertussis* Tohama I; 97%, *Taylorella equigenitalis* MCE9]  +  **RpsM**  [100%, *Bordetella pertussis* Tohama I; 93%, *Achromobacter arsenitoxydans* SY8] | 61  +  55  +  54  +  63 | 0.447  +  0.306  +  0.308  +  0.374 |
| 123421 – 124398  [978] | BB0121 | **NAD-dependent epimerase / dehydratase family protein** [100%, *Bordetella parapertussis* 12822; 90%, *Achromobacter xylosoxidans* C54] | 59 | 0.465 |
| 125694 – 150742  [25049] | BB0123 to BB0141 | **Asparagine synthetase** [79%, *Bordetella parapertussis*; 71%, *Herbaspirillum seropedicae* SmR1]  +  **UDP-glucose 4-epimerase** [34%, *Bacteroides fluxus* YIT 12057]  +  **Phosphoesterase** [35%, *Bacteroides fluxus* YIT 12057]  +  **Carbamoyl phosphate synthase-like protein** [56%, *Bacteroides fluxus* YIT 12057]  +  **C-methyltransferase** [33%, *Bradyrhizobium japonicum* USDA 110]  +  **WbmS / hypothetical protein** [62%,*Bordetella parapertussis* 12822; 44%,*Helicobacter pullorum* MIT 98-5489]  +  **WbmR / formyl transferase** [64%,*Bordetella parapertussis* 12822; 29%,*Citrobacter youngae* ATCC 29220]  +  **WbmO / hypothetical protein** [82%, *Bordetella parapertussis* 12822; 21%, *Methanosarcina acetivorans* C2A]  +  **WbmN / ABC transporter ATP-binding protein** [99%, *Bordetella parapertussis* 12822; 35%, *Aquifex aeolicus* VF5]  +  **WbmM / ABC transporter ATP-binding protein** [100%, *Bordetella parapertussis* 12822; 45%, *Methanosphaera stadtmanae* DSM 3091]  +  **WbmL / ABC transporter** [100%, *Bordetella parapertussis* 12822; 32%, *Clostridium spiroforme* DSM 1552]  +  **WbmK / hypothetical protein** [99%, *Bordetella parapertussis* 12822; 37%, *Laribacter hongkongensis* HLHK9]  +  **WbmJ / hypothetical protein** [99%, *Bordetella parapertussis* 12822; 31%, *Laribacter hongkongensis* HLHK9]  +  **WbmI / asparagine synthetase** [100%, *Bordetella parapertussis* 12822; 60%, *Laribacter hongkongensis* HLHK9]  +  **WbmH / nucleotide sugar epimerase/dehydratase** [100%, *Bordetella parapertussis* 12822; 78%, *Phaeospirillum molischianum* DSM 120]  +  **WbmG / nucleotide sugar epimerase/dehydratase** [100%, *Bordetella parapertussis* 12822; 75%, *Phaeospirillum molischianum* DSM 120]  +  **WbmF / nucleotide sugar epimerase/dehydratase** [100%, *Bordetella parapertussis* 12822; 66%, *Parachlamydia acanthamoebae* UV-7]  +  **WbmE / papain-like transglutaminase superfamily protein** [99%, *Bordetella parapertussis* 12822; 41%, *Acinetobacter lwoffii* SH145]  +  **WbmD / hypothetical protein** [99%, *Bordetella parapertussis* 12822; 37%, *Streptococcus pseudopneumoniae* SK674] | 55  +  61  +  58  +  59  +  57  +  51  +  56  +  50  +  58  +  60  +  54  +  61  +  60  +  55  +  61  +  63  +  60  +  56  +  55 | 0.242  +  0.182  +  0.229  +  0.258  +  0.181  +  0.155  +  0.203  +  0.175  +  0.238  +  0.279  +  0.226  +  0.240  +  0.256  +  0.299  +  0.528  +  0.461  +  0.489  +  0.192  +  0.185 |
| 393007 – 394755  [1749] | BB0381  +  BB0382 | **Hypothetical protein** [40%, *Phycisphaera mikurensis* NBRC 102666]  +  **Hypothetical protein** [45%, *Phycisphaera mikurensis* NBRC 102666] | 54  +  54 | 0.223  +  0.286 |
| 515442 – 530604  [15163] | BB0482 to BB0500 | **Hypothetical protein** [60%, *Bacillus cereus* AH1273]  +  **UsG protein** [61%, *Thiomicrospira crunogena* XCL-2]  +  **Hypothetical protein** [54%, *Pelobacter carbinolicus* DSM 2380]  +  **Hypothetical protein** [75%, *Bordetella avium* 197N; 31%, *Myxococcus fulvus* HW-1]  +  **Hypothetical protein** [95%, *Bordetella avium* 197N; 66%, *Alcaligenes faecalis subsp. faecalis* NCIB 8687]  +  **Hypothetical protein** [85%, *Bordetella avium* 197N; 67%, *Alcaligenes faecalis subsp. faecalis* NCIB 8687]  +  **Hypothetical protein** [81%, *Bordetella parapertussis* 12822; 33%, *Escherichia fergusonii* B253]  +  **VirB6** [98%, *Achromobacter xylosoxidans* C54]  +  **Eex protein** [93%, *Achromobacter xylosoxidans* C54]  +  **Hypothetical protein** [99%, *Bordetella avium* 197N; 98%, *Achromobacter xylosoxidans* C54]  +  **VirB5** [96%, *Achromobacter xylosoxidans* C54]  +  **Hypothetical protein** [96%, *Bordetella avium* 197N; 44%, *Xanthomonas gardneri* ATCC 19865]  +  **VirD2** [96%, *Achromobacter xylosoxidans* C54]  +  **Hypothetical protein** [97%, *Achromobacter xylosoxidans* C54]  +  **Hypothetical protein** [97%, *Bordetella avium* 197N] [97%, *Achromobacter xylosoxidans* C54]  +  **Hypothetical protein** [88%, *Bordetella avium* 197N; 64%, *Alcaligenes faecalis subsp. faecalis* NCIB 8687]  +  **Hypothetical protein** [76%, *Pseudomonas fluorescens* Pf0-1]  +  **Hypothetical protein** [47%, *Klebsiella pneumoniae*]  +  **Hypothetical protein** [97%, *Bordetella avium* 197N; 97%, *Achromobacter xylosoxidans* C54]  +  **Probable integrase / recombinase** [94%, *Achromobacter piechaudii* ATCC 43553] | 50  +  52  +  51  +  47  +  56  +  68  +  66  +  53  +  57  +  57  +  57  +  57  +  60  +  61  +  54  +  56  +  53  +  53  +  54  +  63 | 0.157  +  0.195  +  0.150  +  0.123  +  0.148  +  0.091  +  0.118  +  0.227  +  0.347  +  0.199  +  0.355  +  0.329  +  0.263  +  0.277  +  0.168  +  0.180  +  0.172  +  0.182  +  0.371  +  0.435 |
| 544370 – 554443  [10073] | BB0515 to BB0525 | **Hypothetical protein** [96%, *Bordetella parapertussis* 12822; 88%, *Comamonas testosteroni* KF-1]  +  **Transcriptional regulator** [99%, *Bordetella parapertussis* 12822; 29%, *Chloroflexus aggregans* DSM 9485]  +  **Dioxygenase subunit** [99%, *Bordetella parapertussis* 12822; 43%, *Methylibium petroleiphilum* PM1]  +  **Hydroxylase** [98%, *Bordetella parapertussis* 12822; 35%, *Methylibium petroleiphilum* PM1]  +  **Hypothetical protein** [99%, *Bordetella parapertussis* 12822; 30%, *Dinoroseobacter shibae* DFL 12]  +  **Hypothetical protein** [99%, *Bordetella parapertussis* 12822; 26%, *Eubacterium yurii subsp. margaretiae* ATCC 43715]  +  **Integral membrane protein** [97%, *Bordetella pertussis* Tohama I; 36%, *Grimontia hollisae* CIP 101886]  +  **Oxidoreductase** [97%, *Bordetella parapertussis* 12822; 29%, *Methylobacterium nodulans* ORS 2060]  +  **Ferredoxin** [99%, *Bordetella parapertussis* 12822; 36%, *Methylibium petroleiphilum* PM1]  +  **Hypothetical protein** [99%, *Bordetella parapertussis* 12822; 70%, *Ralstonia pickettii* 12J]  +  **Hypothetical protein** [98%, *Bordetella parapertussis* 12822; 41%, *Alicycliphilus denitrificans* BC] | 61  +  54  +  52  +  45  +  52  +  47  +  49  +  51  +  45  +  52  +  53 | 0.295  +  0.156  +  0.188  +  0.187  +  0.194  +  0.124  +  0.113  +  0.154  +  0.171  +  0.186  +  0.126 |
| 966029 – 981008  [14970] | BB0908 to BB0913 | **Helicase** [83%, *Escherichia coli* CFT073]  +  **Hypothetical protein** [75%, *Escherichia coli* CFT073]  +  **Aaa-family ATPase** [69%, *Thermodesulfatator indicus* DSM 15286]  +  **Serine protease** [90%, *Nitrosomonas eutropha* C91]  +  **Type III restriction-modification system methyltransferase** [61%, *Yersinia rohdei* ATCC 43380]  +  **Type III restriction-modification system stylti enzyme** [83%, *Xanthomonas oryzae pv. oryzicola* BLS256] | 56  +  61  +  53  +  54  +  54  +  58 | 0.374  +  0.443  +  0.170  +  0.195  +  0.234  +  0.444 |
| 1297681 – 1300356  [2676] | BB1205  +  BB1206 | **ATP/GTP-binding hypothetical protein** [50%, *Geobacter sulfurreducens* PCA]  +  **Hypothetical protein with membrane lipoprotein lipid attachment site** [51%, *Pseudomonas fluorescens* F113] | 48  +  43 | 0.094  +  0.096 |
| 1576648 – 1578006  [1359] | BB1473 | **Oxidoreductase OrdL** [38%, *Colwellia psychrerythraea* 34H] | 61 | 0.294 |
| 1765780 – 1766739  [960] | BB1661 | **Bacteriophage HK022 integrase** [84%, *Achromobacter piechaudii* ATCC 43553] | 59 | 0.268 |
| 1775766 – 1777050  [1285] | BB1677  +  BB1678 | **Hypothetical protein** [31%, *Acinetobacter baumannii* 6014059]  +  **Phage repressor** [58%, *Achromobacter xylosoxidans* A8] | 49  +  60 | 0.159  +  0.263 |
| 1792737 – 1793476  [740] | BB1704  +  BB1705 | **Hypothetical protein** [39%, *Faecalibacterium prausnitzii* M21/2]  +  **Hypothetical protein** [46%, *Delftia acidovorans* SPH-1] | 58  +  49 | 0.160  +  0.140 |
| 1972922 – 1977927  [5006] | BBr01 to BBr02 | **16S rRNA** + **two tRNAs** + **23S rRNA** | 54 | NA |
| 2055721 – 2056551  [831] | BB1933 | **Putative hydrolase** [99%, *Bordetella parapertussis* 12822; 63%, *Cupriavidus metallidurans* CH34] | 61 | 0.264 |
| 2153607 – 2153852  [246] | BB2016 | **Cold-shock protein** [100%, *Bordetella pertussis* Tohama I; 96%, *Achromobacter xylosoxidans* A8] | 52 | 0.254 |
| 2192119 – 2193210  [1092] | BB2044 | **Hypothetical protein** [96%, *Bordetella pertussis* Tohama I; 27%, *Clostridium sporogenes* ATCC 15579] | 54 | 0.188 |
| 2343690 – 2345480  [10653] | BB2198 | **Phage–related hypothetical protein**  [57%, *Salmonella enterica subsp. enterica serovar Newport* str. CVM 19443] | 57 | 0.201 |
| 2361261 – 2362873  [1613] | BB2226 to BB2228 | **Phage-related hypothetical protein** [98%, *Bordetella pertussis* Tohama I] [55%, *Xanthomonas fuscans subsp. aurantifolii* str. ICPB 10535]  +  **Hypothetical protein** [98%, *Bordetella pertussis* Tohama I] [50%. *Methyloversatilis universalis* FAM5]  +  **Hypothetical protein** [99%, *Bordetella pertussis* Tohama I] [42%, *Achromobacter xylosoxidans* C54] | 59  +  56  +  57 | 0.218  +  0.260  +  0.294 |
| 2365819 – 2373075  [7257] | BB2231  to  BB2239 | **Bacteriophage hypothetical protein** [87%, *Bordetella pertussis* Tohama I; 34%, *Klebsiella oxytoca* 10-5250]  +  **Bacteriophage hypothetical protein** [89%, *Bordetella pertussis* Tohama I; 46%, *Magnetospirillum magneticum* AMB-1]  +  **Bacteriophage hypothetical protein** [84%, *Bordetella pertussis* Tohama I; 43%, *Magnetospirillum magneticum* AMB-1]  +  **Bacteriophage hypothetical protein** [94%, *Bordetella pertussis* Tohama I; 44%, *Magnetospirillum magneticum* AMB-1]  +  **Hypothetical protein** [99%, *Bordetella pertussis* Tohama I; 32%, *Achromobacter xylosoxidans* C54]  +  **Hypothetical protein** [99%, *Bordetella pertussis* Tohama I; 43%, *Desulfovibrio magneticus* RS-1]  +  **Hypothetical protein** [99%, *Bordetella pertussis* Tohama I; 32*%, Gallibacterium anatis* UMN179]  +  **Hypothetical protein** [99%, *Bordetella pertussis* Tohama I; 75%, *Achromobacter xylosoxidans* AXX-A] | 56  +  55  +  55  +  58  +  59  +  59  +  57  +  54 | 0.159  +  0.167  +  0.150  +  0.159  +  0.268  +  0.182  +  0.136  +  0.187 |
| 2379173 – 2379418  [246] | BB2249 | **Cold-shock protein** [100%, *Bordetella parapertussis* 12822; 93%, *Achromobacter piechaudii* ATCC 43553] | 54 | 0.356 |
| 2472130 – 2472804  [675] | BB2322 | **Hypothetical protein** [99%, *Bordetella parapertussis* 12822; 41%, *Marinobacter algicola* DG893] | 57 | 0.239 |
| 2642893 – 2648414  [5522] | BB2490 to BB2494 | **Abortive infection phage resistance protein** [53%, *Shewanella baltica* OS183]  +  **Transposase/Integrase (pseudogene)**  Similar to *Xanthomonas campestris* transposase  +  **Transposase** [93%, *Achromobacter arsenitoxydans* SY8]  +  **Phage-related DNA-binding protein** [70%, *Pseudomonas savastanoi pv. savastanoi* NCPPB 3335]  +  **Phage-like hypothetical protein** [38%, *Escherichia coli* O127:H6 str. E2348/69] | 52  +  58  +  55  +  55  +  53 | 0.136  +  -  +  0.360  +  0.151  +  0.133 |
| 2884328 – 2884699  [372] | BB2721 | **Transcriptional regulator** [98%, *Bordetella parapertussis* 12822; 33%, *Pelobacter propionicus* DSM 2379] | 61 | 0.389 |
| 3183761 – 3184366  [606] | BB2992 | **Fimbrial protein** [99%, *Bordetella parapertussis*; 35%, *Escherichia coli* 83972] | 58 | 0.230 |
| 3196207 – 3196890  [684] | BB2994 | **Virulence factors transcription regulator** [100%, *Bordetella parapertussis* 12822; 62%, *Pseudomonas fluorescens* Pf0-1] | 58 | 0.415 |
| 3617172 – 3622466  [5294] | BBr04 to BBr06 | **5S rRNA** + **23S rRNA** + **two tRNAs** + **16S rRNA** | 54 | NA |
| 3686694 – 3687074  [381] | BB3443 | **50S ribosomal protein L19** [100%, *Bordetella pertussis* Tohama I; 91%, *Achromobacter piechaudii* ATCC 43553] | 61 | 0.580 |
| 3719946 – 3720527  [582] | BB3474 | **Outer membrane protein A**  **precursor** [100%, *Bordetella parapertussis* 12822; 95%, *Achromobacter piechaudii* ATCC 43553] | 61 | 0.456 |
| 3725058 – 3738505  [13448] | BB3480 to BB3494 | **Hypothetical protein** [33%, *Desulfotalea psychrophila* LSv54]  +  **Hypothetical protein** [38%, *Wolinella succinogenes* DSM 1740]  +  **Phage lysozyme** [90%, *Bordetella pertussis* Tohama I; 77%, *Achromobacter xylosoxidans* AXX-A]  +  **Homolog of Serratia marcescens phage-holin analog protein regA** [98%, *Bordetella pertussis* Tohama I; 32%, *Gallibacterium anatis* UMN179]  +  **Hypothetical protein** [93%, *Bordetella pertussis* Tohama I; 44%, *Desulfovibrio magneticus* RS-1]  +  **Hypothetical protein** [98%, *Bordetella pertussis* Tohama I; 47%, *Pelagibacterium halotolerans* B2]  +  **Hypothetical protein** [97%, *Bordetella pertussis* Tohama I; 31%, *Achromobacter xylosoxidans* C54]  +  **Hypothetical phage protein** [94%, *Bordetella pertussis* Tohama I; 44%, *Magnetospirillum magneticum* AMB-1]  +  **Hypothetical phage protein** [84%, *Bordetella pertussis* Tohama I; 43%, *Magnetospirillum magneticum* AMB-1]  +  **Hypothetical phage protein** [89%, *Bordetella pertussis* Tohama I; 46%, *Magnetospirillum magneticum* AMB-1]  +  **Hypothetical phage protein** [87%, *Bordetella pertussis* Tohama I; 34%, *Klebsiella oxytoca* 10-5250]  +  **Phage hk97 tail length tape measure-related protein** [71%, *Bordetella pertussis* Tohama I; 35%, *Escherichia coli* E482]  +  **Hypothetical phage protein** [96%, *Bordetella pertussis* Tohama I; 49%, *Pseudomonas aeruginosa* PA7]  +  **Hypothetical phage protein** [99%, *Bordetella pertussis* Tohama I; 42%, *Achromobacter xylosoxidans* C54]  +  **Phosphoglycerate mutase family protein** [98%, *Bordetella pertussis* Tohama I; 50%, *Methyloversatilis universalis* FAM5]  +  **Hypothetical phage protein** [98%, *Bordetella pertussis* Tohama I; 55%, *Xanthomonas fuscans subsp. aurantifolii* str. ICPB 10535] | 53  +  65  +  64  +  55  +  56  +  60  +  60  +  58  +  55  +  55  +  56  +  62  +  60  +  57  +  56  +  59 | 0.157  +  0.204  +  0.256  +  0.189  +  0.134  +  0.186  +  0.284  +  0.159  +  0.150  +  0.167  +  0.159  +  0.239  +  0.329  +  0.294  +  0.260  +  0.218 |
| 3753781 – 3756123  [2343] | BB3522 to BB3526 | **Phage repressor** [51%, *Bordetella avium* 197N; 55%, *Acinetobacter baumannii* ABNIH3]  +  **Hypothetical transcriptional regulator** [33%, *Bordetella avium* 197N; 32%, *Ralstonia solanacearum* K60-1]  +  **Hypothetical protein** [42%, *Klebsiella pneumoniae* subsp. *rhinoscleromatis* ATCC 13884]  +  **Hypothetical protein** [47%, *Bordetella avium* 197N; 42%, *Rahnella aquatilis* HX2]  +  **Hypothetical protein** [23%, *Parabacteroides merdae* ATCC 43184] | 57  +  57  +  57  +  55  +  60 | 0.208  +  0.166  +  0.145  +  0.187  +  0.608 |
| 3859012 – 3861507  [2496] | BB3640 to BB3644 | **Hypothetical protein** [27%, *Mycobacterium parascrofulaceum* ATCC BAA-614]  +  **Hypothetical protein** [43%, *Pseudomonas synxantha* BG33R]  +  **Phage-related hypothetical protein** [49%, *Pasteurella multocida* subsp. *multocida* str. 3480]  +  **Hypothetical protein** [56%, *Alicycliphilus denitrificans* BC]  +  **Hypothetical protein** [54%, *Pseudomonas synxantha* BG33R] | 53  +  56  +  59  +  60  +  60 | 0.137  +  0.160  +  0.178  +  0.480  +  0.325 |
| 4023544 – 4024419  [876] | BB3801  +  BB3802 | **Intracellular septation protein** [100%, *Bordetella pertussis* Tohama I; 74%, *Achromobacter xylosoxidans* A8]  +  **BolA-like protein** [96%, *Bordetella pertussis* Tohama I; 79%, *Achromobacter xylosoxidans* C54] | 61  +  55 | 0.538  +  0.158 |
| 4135574 – 4139436  [3863] | BB3893 to BB3895 | **Alcaligin biosynthesis protein AlcA** [99%, *Bordetella pertussis* Tohama I; 79%, *Achromobacter xylosoxidans* C54]  +  **Alcaligin biosynthesis protein AlcB** [100%, *Bordetella pertussis* Tohama I; 76%, *Achromobacter xylosoxidans* C54]  +  **Alcaligin biosynthesis protein AlcC** [100%, *Bordetella parapertussis* 12822; 81%, *Achromobacter xylosoxidans* AXX-A] | 58  +  61  +  59 | 0.308  +  0.369  +  0.325 |
| 4401461 – 4406711  [5251] | BB4137 to BB4142 | **Hypothetical protein** [93%, *Bordetella pertussis* Tohama I; 90%, *Achromobacter xylosoxidans* A8]  +  **Phage-related integrase** [69%, *Alcaligenes faecalis subsp. faecalis* NCIB 8687]  +  **Hypothetical protein** [47%, *Alcaligenes faecalis subsp. faecalis* NCIB 8687]  +  **Hypothetical phage protein** [38%, *Neisseria sicca* ATCC 29256]  +  **Hypothetical protein** [30%, *Rhodobacter capsulatus* SB 1003]  +  **Hypothetical protein** [46%, *Methylovorus glucosetrophus* SIP3-4] | 61  +  64  +  60  +  60  +  61  +  48 | 0.465  +  0.407  +  0.279  +  0.234  +  0.203  +  0.172 |
| 4409209 – 4410352  [1144] | BB4148 to BB4149A | **Hypothetical protein**  [36%, *Pectobacterium wasabiae* WPP163]  +  **Hypothetical protein**  [26%, *Pectobacterium wasabiae* WPP163]  +  **Hypothetical protein**  [50%, *Shewanella woodyi* ATCC 51908] | 46  +  48  +  51 | 0.123  +  0.103  +  0.102 |
| 4481211 – 4486505  [5295] | BBr07 to BBr09 | **5S rRNA** + **23S rRNA** + **two tRNAs** + **16S rRNA** | 54 | NA |
| 4494933 – 4495397  [465] | BB4221 | **Hypothetical protein**  [99%, *Bordetella pertussis* Tohama I; 90%, *Achromobacter piechaudii* ATCC 43553] | 59 | 0.389 |
| 5337182 – 5337388  [207] | BB5013 | **Cold shock–like protein**  [100%, *Bordetella parapertussis* 12822; 99%, *Achromobacter xylosoxidans* C54] | 55 | 0.279 |
| **Total HGT =** 150,346 bp | | | | |

**Table D.** G+C content and Codon Adaptation Index (CAI) of the regions in the *Taylorella equigenitalis* MCE9 genome predicted to have been acquired via HGT. The average G+C % of the genome is 38. The mean CAI of the genome is 0.452. NA = not applicable.

| **Approximate position**  **[Length of total HGT in bp]** | **Locus Tags** | **Nearest BLASTP hit** | **G+C %** | **MCAI** |
| --- | --- | --- | --- | --- |
| 1001-4706  [3705] | TEQUI_0001  +  TEQUI_0002 | **Hypothetical protein** [91%, *Taylorella*  *asinigenitalis* MCE3] [32%, *Advenella kashmirensis* WT001]  +  **Hypothetical protein** [90%, *Taylorella asinigenitalis* MCE3] [36%, *Advenella kashmirensis* WT001] | 31  +  33 | 0.490  +  0.481 |
| 31363-37301  [5939) | TEQUI_0023  to  TEQUI_0028 | **EF hand domain protein** [94%, *Taylorella asinigenitalis* MCE3] [36%, *Pantoea ananatis* LMG 20103]  +  **Hypothetical protein** [97%, *Taylorella asinigenitalis* MCE3] [25%, *Streptococcus macedonicus* ACA-DC 198]  +  **Hypothetical protein** [*Taylorella asinigenitalis* 14/45] [25%, *Bacillus licheniformis* DSM 13]  +  **Putative cytoplasmic protein** [98%, *Taylorella asinigenitalis* MCE3] [55%, *Xanthomonas albilineans* GPE PC73]  +  **EF hand domain protein** [85%, *Taylorella asinigenitalis* MCE3] [38%, *Pantoea ananatis* LMG 20103]  +  **Hypothetical protein** [89%, *Taylorella asinigenitalis* MCE3] [25%, *Bacillus cereus* Rock4-18] | 30  +  28  +  28  +  33  +  28  +  28 | 0.494  +  0.511  +  0.515  +  0.492  +  0.517  +  0.523 |
| 41091-42576  [1486) | TEQUI_0032  +  TEQUI_0033 | **Hypothetical protein** [44%, *Campylobacter curvus* 525.92]  +  **Pyridoxamine 5'-phosphate oxidase family protein** [85%, *Taylorella asinigenitalis* MCE3] [46%, *Campylobacter curvus* 525.92] | 31  +  33 | 0.460  +  0.506 |
| 64780-65334  [555] | TEQUI_0053 | **Lipoprotein signal peptidase** [77%, *Taylorella asinigenitalis* MCE3] [48%, *Achromobacter xylosoxidans* AXX-A] | 32 | 0.457 |
| 73655-76318  [2664) | TEQUI_0060  +  TEQUI_0061 | **ATP-dependent Clp protease ATP-binding subunit ClpA** [64%, *Taylorella asinigenitalis* MCE3] [44%, *Rubrivivax gelatinosus* IL144]  +  **COLD-SHOCK DNA-binding domain protein** [91%, *Taylorella asinigenitalis* MCE3] [69%, *Bordetella pertussis* Tohama I] | 33  +  32 | 0.480  +  0.648 |
| 84114-85362  [1249) | TEQUI_0069  to  TEQUI_72 | **Modification methylase BepI-like protein** [85%, *Taylorella asinigenitalis* MCE3] [58%, *Belliella baltica* DSM 15883]  +  **Hypothetical protein** [79%, *Taylorella asinigenitalis* MCE3] [45%, *Belliella baltica* DSM 15883]  +  **Modification methylase BepI-like protein** [87%, *Taylorella asinigenitalis* MCE3] [54%, *Mycoplasma canis* UF31] [48%, *Belliella baltica* DSM 15883]  +  **Hypothetical protein** [48%, *Aspergillus niger* CBS 513.88] | 33  +  31  +  33  +  32 | 0.634  +  0.540  +  0.487  +  0.566 |
| 136359-138120  [1762) | TEQUI_0123  to  TEQUI_0125 | **Hypothetical protein** [32%, *Methanosarcina barkeri str. Fusaro*]  +  **Hypothetical protein** [44%, *Bacillus cereus* ATCC 10876]  +  **Leucine rich repeat variant** [37%, *Ktedonobacter racemifer* DSM 44963] | 29  +  30  +  33 | 0.581  +  0.516  +  0.456 |
| 138679-141054  [2376) | TEQUI_0127  to  TEQUI_0129 | **Hypothetical protein** [28%, *Drosophila mojavensis*]  +  **Hypothetical protein** [33%, *Natranaerobius thermophilus* JW/NM-WN-LF]  +  **Hypothetical protein** [35%, *Saprospira grandis* str. Lewin] | 30  +  29  +  28 | 0.600  +  0.542  +  0.558 |
| 157157-157887  [731) | TEQUI_0152  +  TEQUI_153 | **Leucine rich repeat variant** [29%, *Methanosaeta thermophila* PT]  +  **Leucine rich repeat variant** [36%, *Ktedonobacter racemifer* DSM 44963] | 30  +  31 | 0.430  +  0.454 |
| 158969- 165437  [6469] | TEQUI_0155  to  TEQUI_0162 | **Hypothetical protein** [29%, *Advenella kashmirensis* WT001]  +  **Hypothetical protein** [31%, *Kingella denitrificans* ATCC 33394]  +  **Relaxase/mobilization nuclease domain containing protein** [40%, *Neisseria weaveri* LMG 5135]  +  **Hypothetical protein** [36%, *Escherichia coli*]  +  **Hypothetical protein** [34%, *Neisseria wadsworthii* 9715]  +  **Hypothetical protein** [36%, *Kingella oralis* ATCC 51147]  +  **Hypothetical protein** [85%, *Taylorella asinigenitalis* MCE3] [44%, *Leptotrichia buccalis* C-1013-b]  +  **Hypothetical protein** [55%, *Helicobacter canadensis* MIT 98-5491] | 32  +  28  +  32  +  32  +  29  +  22  +  29  +  28 | 0.517  +  0.497  +  0.499  +  0.434  +  0.532  +  0.731  +  0.502  +  0.551 |
| 166605- 171494  [4890) | TEQUI_0164  to  TEQUI_0169 | **Putative phage repressor** [42%, *Bordetella avium* 197N]  +  **AbiQ-like protein** [70%, *Taylorella asinigenitalis* 14/45] [41%, *Lactococcus lactis subsp. lactis* CV56]  +  **Protein kinase** [40%, *Salmonella enterica subsp. enterica serovar Dublin*]  +  **Putative phage repressor** [45%, *Bordetella petrii* DSM 12804]  +  **Hypothetical protein** [29%, *Burkholderia vietnamiensis* G4]  +  **Phage integrase** [33%, *Xanthobacter autotrophicus* Py2] | 33  +  23  +  33  +  33  +  31  +  32 | 0.464  +  0.567  +  0.529  +  0.516  +  0.471  +  0.496 |
| 445441-445833  [393) | TEQUI_0429 | **Hypothetical protein** [37%, *Streptococcus infantis* ATCC 700779] | 32 | 0.436 |
| 448318-449076  [759] | TEQUI_0431 | **Hypothetical protein** [46%, *Capnocytophaga sputigena Capno*] | 29 | 0.470 |
| 451841-452179  [339] | TEQUI_0433 | **Hypothetical protein** [40%, *Stigmatella aurantiaca* DW4/3-1] | 30 | 0.467 |
| 642825-646230  [3406] | TEQUI_0621  to  TEQUI_0623 | **Glucose inhibited division protein** [65%, *Taylorella asinigenitalis* MCE3]  +  **tRNA uridine 5-carboxymethylaminomethyl modification enzyme GidA** [90%, *Taylorella asinigenitalis* MCE3] [72%, *Bordetella avium* 197N]  +  **Putative Hit-like protein** [74%, *Taylorella asinigenitalis* 14/45] [50%, *Ralstonia pickettii* 12J] | 30  +  33  +  30 | 0.481  +  0.490  +  0.605 |
| 671801-673249  [1449] | TEQUI_0646  +  TEQUI_0647 | **Hypothetical protein** [43%, *Taylorella asinigenitalis* MCE3] [30%, *Psychrobacter cryohalolentis* K5]  +  **Periplasmic thiol:disulfide interchange protein DsbA** [74%, *Taylorella asinigenitalis* MCE3] [48%, *Achromobacter piechaudii* HLE] | 31  +  32 | 0.516  +  0.593 |
| 758296-758916  [621] | TEQUI_0726 | **Hypothetical protein** [77%, *Taylorella asinigenitalis* MCE3] [34%, *Bacillus subtilis subsp. spizizenii* TU-B-10] | 30 | 0.469 |
| 761760-766175  [4416] | TEQUI_0729  to  TEQUI_0731 | **Hypothetical protein** [37%, *Taylorella asinigenitalis* 14/45] [25%, *Pantoea ananatis* LMG 5342]  +  **Hypothetical protein** [32%, *Taylorella asinigenitalis* 14/45] [24%, *Photorhabdus asymbiotica subsp. asymbiotica* ATCC 43949]  +  **Hypothetical protein** [32%, *Taylorella asinigenitalis* 14/45] [25%, *Serratia odorifera* DSM 4582] | 30  +  28  +  28 | 0.477  +  0.522  +  0.535 |
| 766809- 768177  [1375] | TEQUI_0733  +  TEQUI_0734 | **Hypothetical protein** [31%, *Shewanella piezotolerans* WP3] [48%, *Pseudoalteromonas haloplanktis* TAC125]  +  **Hypothetical protein** [88%, *Taylorella asinigenitalis* 14/45] [30%, *Selaginella moellendorffii*] | 23  +  29 | 0.662  +  0.572 |
| 789692-790093  [402] | TEQUI_0759 | **Hypothetical protein** [35%, *Burkholderia ambifaria* MEX-5] | 31 | 0.513 |
| 790215-790415  [201] | TEQUI_0761 | **Hypothetical protein** [49%, *Enterobacter cloacae subsp. cloacae* ENHKU01] | 27 | 0.548 |
| 824973- 826128  [1156] | TEQUI_0791  to  TEQUI_0793 | **Hypothetical protein** [57%, *Taylorella asinigenitalis* 14/45] [39%, *Erwinia amylovora* ATCC BAA-2158]  +  **Hypothetical protein** [50%, *Erwinia amylovora* ATCC BAA-2158]  +  **Hypothetical protein** [99%, *Taylorella asinigenitalis* 14/45] [41%, *Erwinia amylovora* ATCC BAA-2158] | 29  +  25  +  32 | 0.636  +  0.500  +  0.539 |
| 828113-829055  [943] | TEQUI_0797  +  TEQUI_0798 | **Hypothetical protein** [38%, *Methanococcoides burtonii* DSM 6242]  +  **Hypothetical protein** [50%, *Taylorella asinigenitalis* 14/45] [25%, *Pasteurella multocida* 36950] | 29  +  32 | 0.331  +  0.354 |
| 866230-868314  [2085] | TEQUI_0834  to  TEQUI_0836 | **Hypothetical protein** [32%, *Pseudoalteromonas atlantica* T6c]  +  **Hypothetical** protein [50%, *Photorhabdus luminescens subsp. laumondii* TTO1]  +  **Hypothetical protein** [30%, *Pseudoalteromonas atlantica* T6c] | 30  +  30  +  30 | 0.450  +  0.495  +  0.429 |
| 897125-897649  [525] | TEQUI_0873  +  TEQUI_0874 | **Putative membrane protein** [59%, *Taylorella asinigenitalis* 14/45] [72%, *Lautropia mirabilis* ATCC 51599] [69%, *Advenella kashmirensis* WT001]  +  **Putative membrane protein** [45%, *Lautropia mirabilis* ATCC 51599] | 33  +  31 | 0.574  +  0.423 |
| 1052325-1066732  [14408] | TEQUI_1010  to  TEQUI_1016 | **O-antigen export system permease protein RfbD** [49%, *Acidovorax citrulli* AAC00-1]  +  **Teichoic acid export ATP-binding protein TagH** [39%, *Acidovorax citrulli* AAC00-1]  +  **Hypothetical protein** [33%, *Acidovorax citrulli* AAC00-1]  +  **Hypothetical protein** [36%, *Octadecabacter arcticus* 238]  +  **Hypothetical protein** [32%, *Vibrio cholerae bv. albensis* VL426]  +  **Asparagine synthetase (glutamine-hydrolyzing)** [47%, *Acidovorax citrulli* AAC00-1]  +  **Glycosyl transferase, group 1** [30%, *Laribacter hongkongensis* HLHK9] | 31  +  33  +  31  +  31  +  33  +  33  +  30 | 0.431  +  0.444  +  0.493  +  0.471  +  0.469  +  0.442  +  0.484 |
| 1069144-1071227  [2084] | TEQUI_1019  +  TEQUI_1020 | **Lipid carrier : UDP-N-acetylgalactosaminyltransferase** [63%, *Photorhabdus luminescens subsp. laumondii* TTO1]  +  **Hypothetical protein** [36%, *Taylorella asinigenitalis* 14/45] [29%, *Arcobacter butzleri* JV22] | 33  +  32 | 0.412  +  0.432 |
| 1072597-1074438  [1842] | TEQUI_1022 | **Probable acetyltransferase** [67%, *Taylorella asinigenitalis* 14/45] [39%, *Pseudomonas aeruginosa* PAO1] | 33 | 0.464 |
| 1167935-1168279  [345] | TEQUI_1110 | **Hypothetical protein** [56%, *Taylorella asinigenitalis* 14/45] [31%, *Achromobacter xylosoxidans* AXX-A] | 30 | 0.484 |
| 1178817-1178984  [168] | TEQUI_1121 | **LSU ribosomal protein L33p** [87%, *Advenella kashmirensis* WT001] | 33 | 0.696 |
| 1202866-1203315  [450] | TEQUI_1147 | **Hypothetical protein** [77%, *Taylorella asinigenitalis* 14/45] [29%, *Advenella kashmirensis* WT001] | 32 | 0.553 |
| 1255810-1256910  [1101] | TEQUI_1200 | **tRNA(Ile)-lysidine synthetase** [47%, *Taylorella asinigenitalis* MCE3] [35%, *Alcaligenes faecalis subsp. faecalis* NCIB 8687] | 33 | 0.409 |
| 1257063-1257386  [324] | TEQUI_1202 | **Hypothetical protein** [72%, *Taylorella asinigenitalis* 14/45] [35%, *Sphingomonas elodea* ATCC 31461] | 29 | 0.502 |
| 1282717-1283034  [318] | TEQUI_1225 | **Thioredoxin** [89%, *Taylorella asinigenitalis* MCE3] [59%, *Ramlibacter tataouinensis* TTB310] | 32 | 0.642 |
| 1303043-1303942  [900] | TEQUI_1248 | **Hypothetical protein** [59%, *Taylorella asinigenitalis* MCE3] [38%, *Escherichia coli* IHE3034] | 32 | 0.481 |
| 1308425-1309276  [852] | TEQUI_1252 | **Hypothetical protein** [45%, *Taylorella asinigenitalis* MCE3] [24%, *Achromobacter piechaudii* HLE] | 29 | 0.485 |
| 1318093-1320027  [1935] | TEQUI_1261 | **Hypothetical protein** [70%, *Taylorella asinigenitalis* 14/45] [36%, *Advenella kashmirensis* WT001] | 32 | 0.508 |
| 1328735-1330594  [1860] | TEQUI_1272 | **DNA helicase IV** [65%, *Taylorella asinigenitalis* MCE3] [32%, *Pseudomonas fluorescens* Q8r1-96] | 32 | 0.412 |
| 1342165-1343533  [1369] | TEQUI_1281  to  TEQUI_1283 | **Hypothetical protein** [57%, *Mycoplasma suis* KI3806]  +  **Hypothetical protein** [38%, *Halobiforma lacisalsi* AJ5]  +  **Hypothetical protein** [81%, *Taylorella asinigenitalis* 14/45] [46%, *Lactobacillus crispatus* JV-V01] | 29  +  33  +  33 | 0.394  +  0.523  +  0.398 |
| 1345754-1347999  [2246] | TEQUI_1286  +  TEQUI_1287 | **Pyrophosphatase** [88%, *Taylorella asinigenitalis* 14/45] [58%, *Butyrivibrio fibrisolvens* 16/4]  +  **Putative ATP/GTP-binding protein** [85%, *Taylorella asinigenitalis* MCE3] [50%, *Veillonella parvula* DSM 2008] | 29  +  32 | 0.581  +  0.481 |
| 1356787-1358677  [1891] | TEQUI_1294  +  TEQUI_1295 | **Putative integral membrane protein** [27%, *Campylobacter curvus* 525.92]  +  **Nitrate transport ATP-binding protein NrtC** [43%, *Campylobacter concisus* UNSWCD] | 33  +  33 | 0.443  +  0.478 |
| 1360707-1364336  [3630] | TEQUI_1298 | **Hypothetical protein** [38%, *Eikenella corrodens* ATCC 23834] | 33 | 0.470 |
| 1365366-1370773  [5408] | TEQUI_1301  to  TEQUI_1304 | **Outer membrane protein** [37%, *Neisseria bacilliformis* ATCC BAA-1200]  +  **ABC transporter, ATP-binding/permease protein** [59%, *Filifactor alocis* ATCC 35896]  +  **ABC transporter, ATP-binding/permease protein** [62%, *Staphylococcus lugdunensis* VCU139]  +  **Hypothetical protein** [60%, *Taylorella asinigenitalis* MCE3] [38%, *Arabidopsis thaliana*] [42%, *Chlorobium luteolum* DSM 273] | 30  +  31  +  32  +  24 | 0.429  +  0.442  +  0.492  +  0.433 |
| 1423768-1424238  [471] | TEQUI_1345 | **Nuclease-like protein** [52%, *Taylorella asinigenitalis* 14/45] [44%, *Neisseria shayeganii* 871] | 31 | 0.501 |
| 1430760-1431146  [387] | TEQUI_1354 | **Invasion gene expression up-regulator, SirB** [77%, *Taylorella asinigenitalis* 14/45] [31%, *Salinisphaera shabanensis* E1L3A] | 30 | 0.543 |
| 1449874-1452593  [2720] | TEQUI_1373  +  TEQUI_1374 | **Tetraacyldisaccharide 4'-kinase** [59%, *Taylorella asinigenitalis* MCE3] [37%, *Achromobacter xylosoxidans* A8]  +  **Exodeoxyribonuclease VII large subunit** [49%, *Taylorella asinigenitalis* MCE3] [38%, *Variovorax paradoxus* EPS] | 33  +  33 | 0.404  +  0.480 |
| 1485813-1487922  [2110] | TEQUI_1405  +  TEQUI_1406 | **Phosphatidate cytidylyltransferase** [75%, *Taylorella asinigenitalis* MCE3] [33%, *Advenella kashmirensis* WT001]  +  **1-deoxy-D-xylulose 5-phosphate reductoisomerase** [76%, *Taylorella asinigenitalis* MCE3] [50%,*Advenella kashmirensis* WT001] | 32  +  33 | 0.424  +  0.450 |
| 1515741-1516514  [774] | TEQUI_1436 | **Hypothetical protein** [76%, *Taylorella asinigenitalis* MCE3] [44%, *Achromobacter piechaudi*i HLE] | 33 | 0.439 |
| 1525365-1526693  [1329] | TEQUI_1443 | **RND efflux system, outer membrane lipoprotein, NodT family** [68%, *Taylorella asinigenitalis* 14/45] [41%, *Advenella kashmirensis* WT001] | 33 | 0.452 |
| 1532863-1535674  [2812] | TEQUI_1446  to  TEQUI_1448 | **Putative RND transporter, membrane fusion protein** [68%, *Taylorella asinigenitalis* MCE3] [43%, *Advenella kashmirensis* WT001]  +  **Hypothetical protein** [81%, *Taylorella asinigenitalis* 14/45] [52%, *Haemophilus paraphrohaemolyticus* HK411]  +  **Hypothetical protein** [60%, *Taylorella asinigenitalis* MCE3] [40%, *Advenella kashmirensis* WT001] | 33  +  33  +  31 | 0.441  +  0.431  +  0.442 |
| 1543632-1545109  [1478] | TEQUI_1457  +  TEQUI_1458 | **ADP-heptose--lipooligosaccharide heptosyltransferase II** [64%, *Taylorella asinigenitalis* 14/45] [43%, *Aggregatibacter actinomycetemcomitans* RhAA1]  +  **Regulatory protein RecX** [59%, *Taylorella asinigenitalis* 14/45] [43%, *Advenella kashmirensis* WT001] | 32  +  32 | 0.439  +  0.392 |
| 1546298-1547682  [1385] | TEQUI_1460  +  TEQUI_1461 | **DNA recombination-dependent growth factor C** [75%, *Taylorella asinigenitalis* MCE3] [39%, *Achromobacter xylosoxidans* C54]  +  **Hypothetical protein** [59%, *Taylorella asinigenitalis* 14/45] [30%, *Advenella kashmirensis* WT001] | 33  +  33 | 0.482  +  0.369 |
| 1549760-1550956  [1197] | TEQUI_1463 | **Aromatic-amino-acid aminotransferase** [76%, *Taylorella asinigenitalis* MCE3] [49%, *Comamonas testosteroni* KF-1] | 33 | 0.559 |
| 1558376-1559847  [1472] | TEQUI_1471  +  TEQUI_1472 | **Hypothetical protein** [49%, *Taylorella asinigenitalis* 14/45] [37%, *Fusobacterium varium* ATCC 27725]  +  **Hypothetical protein** [45%, *Taylorella asinigenitalis* 14/45] [30%, *Weeksella virosa* DSM 16922] | 27  +  33 | 0.517  +  0.491 |
| 1572508-1572675  [168] | TEQUI_1485 | **Hypothetical protein** [67%, *Taylorella asinigenitalis* MCE3] [43*%, Herbaspirillum seropedicae* SmR1] | 29 | 0.534 |
| 1582115-1582813  [699] | TEQUI_1492 | **Hypothetical protein** [43%, *Taylorella asinigenitalis* MCE3] [34*%, Dictyoglomus turgidum* DSM 6724] | 28 | 0.466 |
| 1591168- 1592813  [1646] | TEQUI_1503  +  TEQUI_1504 | **Type II/IV secretion system protein TadC** [78%, *Taylorella asinigenitalis* 14/45] [34%, *Achromobacter piechaudii* ATCC 43553]  +  **Flp pilus assembly protein TadB** [76%, *Taylorella asinigenitalis* MCE3] [32%, *Pseudoalteromonas spongiae* UST010723-006] | 31  +  32 | 0.463  +  0.446 |
| 1596906- 1598209  [1304] | TEQUI_1508  to  TEQUI_1510 | **Hypothetical protein** [44%, *Taylorella asinigenitalis* 14/45] [30%, *Ustilago maydis* 521]  +  **Hypothetical protein** [63%, *Taylorella asinigenitalis* 14/45] [26%, *Advenella kashmirensis* WT001]  +  **Hypothetical protein** [74%, *Taylorella asinigenitalis* MCE3] [34%, *Advenella kashmirensis* WT001] | 28  +  33  +  33 | 0.501  +  0.434  +  0.442 |
| 1601047-1605605  [4559] | TEQUI_1514  to  TEQUI_1518 | **Hypothetical protein** [32%, *Terriglobus roseus* DSM 18391]  +  **Protein C** [39%, *Acinetobacter haemolyticus* ATCC 19194]  +  **Hypothetical protein** [44%, *Caenorhabditis briggsae*]  +  **Hypothetical protein** [40%, *Salmonella enterica subsp. enterica serovar Newport* str. CVM 19536]  +  **Hypothetical protein** [45%, *Alicycliphilus denitrificans* BC] | 29  +  32  +  28  +  29  +  31 | 0.507  +  0.479  +  0.566  +  0.527  +  0.480 |
| 1620228-1620872  [645] | TEQUI_1531 | **Hypothetical conserved protein** [67%, *Taylorella asinigenitalis* MCE3] [32%, *Bordetella petrii* DSM 12804] | 32 | 0.501 |
| 1633225-1633995  [771] | TEQUI_1542 | **Hypothetical protein** [43%, *Taylorella asinigenitalis* 14/45] [27%, *Achromobacter arsenitoxydan*s SY8] | 32 | 0.420 |
| 1654025-1654837  [813] | TEQUI_1563 | **hypothetical protein** [39%, *Haemophilus parahaemolyticus* HK385] | 30 | 0.522 |
| 1672142- 1674746  [2605] | TEQUI_1581  to  TEQUI_1583 | **Hypothetical protein** [29%, *Mahella australiensis* 50-1 BON]  +  **Hypothetical protein** [30%, *Nostoc punctiforme* PCC 73102]  +  **Hypothetical protein** [25%, *Millerozyma farinosa* CBS 7064] | 29  +  30  +  28 | 0.508  +  0.542  +  0.516 |
| 1683726-1684824  [1099] | TEQUI_1594  +  TEQUI_1595 | **Putative cytoplasmic protein** [97%, *Taylorella asinigenitalis* MCE3] [35%, *Yersinia pseudotuberculosis* YPIII]  +  **Hypothetical protein** [46%, *Fluviicola taffensis* DSM 16823] | 33  +  31 | 0.458  +  0.594 |
| 1687701-1688120  [420] | TEQUI_1598 | **Hypothetical protein** [99%, *Taylorella asinigenitalis* MCE3] [76%, *Weeksella virosa* DSM 16922] | 33 | 0.452 |
| 1689892-1690362  [471] | TEQUI_1600 | **Ankyrin** [44%, *Listeria monocytogenes* HCC23] | 30 | 0.538 |
| **Total HGT =** 122,362 bp |  |  |  |  |

**Table E.** *Te* genes that are absent in at least one of the other three genomes.

| **Category** | **Subcategory** | **Subsystem** | | **Role** | **A8** | ***Bb*** | ***Tk*** | ***Te*** |
| --- | --- | --- | --- | --- | --- | --- | --- | --- |
| **Missing in A8 and *Tk*, but present in *Bb* and *Te*** | | | | | | | | |
| Cell Wall and Capsule | Gram–Negative cell wall components | Lipopolysaccharide assembly | Outer membrane protein NlpB, lipoprotein component of the protein assembly complex (forms a complex with YaeT, YfiO, and YfgL) | | - | + | - | + |
| Clustering–based subsystems | no subcategory | A Hypothetical that Clusters with PEP Synthase | FIG137360– hypothetical protein | | - | + | - | + |
| Membrane Transport | Protein and nucleoprotein secretion system, Type IV | Conjugative transfer | IncQ plasmid conjugative transfer DNA nicking endonuclease TraR (pTi VirD2 homolog) | | - | + | - | + |
| Clustering-based subsystems | no subcategory | NusA-TFII Cluster | FIG000325: clustered with transcription termination protein NusA | | - | + | - | + |
| RNA Metabolism | RNA processing and modification | RNA pseudouridine syntheses | Similar to ribosomal large subunit pseudouridine synthase D, type RluD4 | | - | + | - | + |
| **Missing in *Bb* and *Tk*, but present in A8 and *Te*** | | | | | | | | |
| Amino Acids and Derivatives | Glutamine, glutamate, aspartate, asparagine; ammonia assimilation | Glutamine, Glutamate, Aspartate and Asparagine Biosynthesis | Glutamate racemase | | + | - | - | + |
| Amino Acids and Derivatives | Histidine Metabolism | Histidine Degradation | Histidine utilization repressor | | + | - | - | + |
| Amino Acids and Derivatives | Lysine, threonine, methionine, and cysteine | Threonine degradation | Threonine dehydrogenase and related Zn-dependent dehydrogenases | | + | - | - | + |
| Amino Acids and Derivatives | Arginine; urea cycle, polyamines | Arginine and Ornithine Degradation | Lysine–arginine–ornithine–binding periplasmic protein precursor | | + | - | - | + |
| Amino Acids and Derivatives | Glutamine, glutamate, aspartate, asparagine; ammonia assimilation | Glutamine, Glutamate, Aspartate and Asparagine Biosynthesis | Glutamate racemase | | + | - | - | + |
| Amino Acids and Derivatives | Histidine Metabolism | Histidine Degradation | Histidine utilization repressor | | + | - | - | + |
| Amino Acids and Derivatives | Lysine, threonine, methionine, and cysteine | Threonine degradation | Threonine dehydrogenase and related Zn–dependent dehydrogenases | | + | - | - | + |
| DNA Metabolism | DNA repair | DNA repair, bacterial | DNA–cytosine methyltransferase | | + | - | - | + |
| DNA Metabolism | no subcategory | Restriction-Modification System | Type I restriction-modification system, restriction subunit R | | + | - | - | + |
| Miscellaneous | Plant–Prokaryote DOE project | COG3533 | Alpha–xylosidase | | + | - | - | + |
| Miscellaneous | Plant–Prokaryote DOE project | Experimental – Histidine Degradation | Histidine ammonia–lyase | | + | - | - | + |
| Miscellaneous | Plant–Prokaryote DOE project | Experimental – Histidine Degradation | Imidazolonepropionase | | + | - | - | + |
| Metabolism of Aromatic Compounds | no subcategory | Gentisare degradation | 4–hydroxybenzoate transporter | | + | - | - | + |
| Metabolism of Aromatic Compounds | no subcategory | Gentisare degradation | 4-hydroxybenzoate transporter | | + | - | - | + |
| Miscellaneous | Plant-Prokaryote DOE project | COG0523 | Carbon starvation protein A | | + | - | - | + |
| Miscellaneous | Plant-Prokaryote DOE project | Experimental - Histidine Degradation | Histidine ammonia-lyase | | + | - | - | + |
| Miscellaneous | Plant-Prokaryote DOE project | Experimental - Histidine Degradation | Imidazolonepropionase | | + | - | - | + |
| Miscellaneous | Plant-Prokaryote DOE project | Experimental - Histidine Degradation | Urocanate hydratase | | + | - | - | + |
| Respiration | Electron accepting reactions | Terminal cytochrome C oxidases | Copper–containing nitrite reductase | | + | - | - | + |
| **Missing in A8 and *Bb*, but present in *Tk* and *Te*** | | | | | | | | |
| DNA Metabolism | DNA repair | DNA repair, bacterial UmuCD system | Error-prone repair protein UmuD | | **-** | **-** | + | + |
| DNA Metabolism | DNA repair | DNA repair, bacterial UmuCD system | Error-prone, lesion bypass DNA polymerase V (UmuC) | | **-** | **-** | + | + |
| DNA Metabolism | DNA repair | DNA repair, bacterial UvrD and related helicases | DNA helicase IV | | - | - | + | + |
| RNA Metabolism | RNA processing and modification | RNA pseudouridine syntheses | Similar to ribosomal large subunit pseudouridine synthase A | | - | - | + | + |
| Amino Acids and Derivatives | Arginine; urea cycle, polyamines | Arginine and Ornithine Degradation | Histidine ABC transporter, ATP–binding protein HisP | | **-** | **-** | + | + |
| Amino Acids and Derivatives | Arginine; urea cycle, polyamines | Arginine and Ornithine Degradation | Histidine ABC transporter, permease protein HisM | | **-** | **-** | + | + |
| Amino Acids and Derivatives | Arginine; urea cycle, polyamines | Arginine and Ornithine Degradation | Histidine ABC transporter, permease protein HisQ | | **-** | **-** | + | + |
| Amino Acids and Derivatives | Proline and 4–hydroxyproline | Proline, 4–hydroxyproline uptake and utilization | Proline/sodium symporter PutP | | - | - | + | + |
| Amino Acids and Derivatives | Alanine, serine, and glycine | Glycine cleavage system | Sodium/glycine symporter GlyP | | **-** | **-** | + | + |
| Miscellaneous | no subcategory | ZZ gjo need homes | Putative membrane protein YfcA | | - | - | + | + |
| **Missing in all the other three (i.e., A8, *Bb* and *Tk*)** | | | | | | | | |
| Amino Acids and Derivatives | Lysine, threonine, methionine, and cysteine | Lysine degradation | L-pipecolate oxidase | | **-** | **-** | **-** | + |
| Cell Wall and Capsule | Gram-Negative cell wall components | KDO2-Lipid A biosynthesis | DNA internalization-related competence protein ComEC/Rec2 | | **-** | **-** | **-** | + |
| Cell Wall and Capsule | Gram-Negative cell wall components | KDO2-Lipid A biosynthesis | O-antigen flippase Wzx | | **-** | **-** | **-** | + |
| Clustering-based subsystems | no subcategory | A Hypothetical that Clusters with PEP Synthase | FIG137360: hypothetical protein | | **-** | **-** | **-** | + |
| Cofactors, Vitamins, Prosthetic Groups, Pigments | Biotin | Biotin biosynthesis | Biotin synthesis protein BioG | | **-** | **-** | **-** | + |
| Cofactors, Vitamins, Prosthetic Groups, Pigments | Coenzyme A | Coenzyme A Biosynthesis | Pantothenate:Na+ symporter | | **-** | **-** | **-** | + |
| Protein Metabolism | Protein degradation | Dipeptidases | Aminoacyl-histidine dipeptidase (Peptidase D) | | **-** | **-** | **-** | + |
| Virulence, Disease and Defense | Resistance to antibiotics and toxic compounds | Lysozyme inhibitors | Periplasmic lysozyme inhibitor of c-type lysozyme | | **-** | **-** | **-** | + |
| Virulence, Disease and Defense | Resistance to antibiotics and toxic compounds | Multidrug Resistance Efflux Pumps | Multi antimicrobial extrusion protein (Na(+)/drug antiporter), MATE family of MDR efflux pumps | | **-** | **-** | **-** | + |
| Membrane Transport | TRAP transporters | A TRAP transporter and a hypothetical | DUF1850 domain–containing protein | | **-** | **-** | **-** | + |
| Membrane Transport | TRAP transporters | A TRAP transporter and a hypothetical | TRAP transporter solute receptor, unknown substrate 1 | | **-** | **-** | **-** | + |
| Membrane Transport | TRAP transporters | A TRAP transporter and a hypothetical | TRAP transporter, 4TM/12TM fusion protein, unknown substrate 1 | | **-** | **-** | **-** | + |
| Nucleosides and Nucleotides | Pyrimidines | pyrimidine conversions | Bis(5'–nucleosyl)–tetraphosphatase (asymmetrical) | | **-** | **-** | **-** | + |
| Protein Metabolism | Protein processing and modification | Methionine sulfoxide reductases | Thiol–disulfide oxidoreductase associated with MetSO reductase | | **-** | **-** | **-** | + |
| **Missing only in *Tk*** | | | | | | | | |
| Amino Acids and Derivatives | Arginine; urea cycle, polyamines | Putrescine utilization pathways | Gamma-aminobutyrate:alpha-ketoglutarate aminotransferase | | + | + | - | + |
| Amino Acids and Derivatives | Lysine, threonine, methionine, and cysteine | Lysine Biosynthesis DAP Pathway | 2,3,4,5-tetrahydropyridine-2,6-dicarboxylate N-acetyltransferase | | + | + | - | + |
| Amino Acids and Derivatives | Lysine, threonine, methionine, and cysteine | Lysine Biosynthesis DAP Pathway | 2,3,4,5–tetrahydropyridine–2,6–dicarboxylate N–acetyltransferase | | + | + | - | + |
| Cofactors, Vitamins, Prosthetic Groups, Pigments | Riboflavin, FMN, FAD | Riboflavin, FMN and FAD metabolism | Riboflavin synthase | | + | + | - | + |
| Protein Metabolism | Protein degradation | Putative TldE–TldD proteolytic complex | TldD protein, part of proposed TldE/TldD proteolytic complex | | + | + | - | + |
| Protein Metabolism | Protein degradation | Putative TldE–TldD proteolytic complex | TldE/PmbA protein, part of proposed TldE/TldD proteolytic complex | | + | + | - | + |
| Protein Metabolism | Protein folding | Peptidyl–prolyl cis–trans isomerase | FKBP–type peptidyl–prolyl cis–trans isomerase slpA | | + | + | - | + |
| Miscellaneous | Plant–Prokaryote DOE project | COG0523 | GTP cyclohydrolase I type 2 | | + | + | - | + |
| Miscellaneous | no subcategory | Broadly distributed proteins not in subsystems | UPF0434 protein YcaR | | + | + | - | + |
| Miscellaneous | no subcategory | Phosphoglycerate mutase protein family | Carboxyl–terminal protease | | + | + | - | + |
| Miscellaneous | Plant–Prokaryote DOE project | At5g04520 AT1G06240 | Colicin V production protein | | + | + | - | + |
| Cell Division and Cell Cycle | no subcategory | Bacterial Cytoskeleton | Sporulation initiation inhibitor protein Soj | | + | + | - | + |
| Cell Wall and Capsule | no subcategory | Peptidoglycan Biosynthesis | UDP-N-acetylmuramoylalanyl-D-glutamate--2,6-diaminopimelate ligase | | + | + | - | + |
| Clustering-based subsystems | Tricarboxylate transporter | CBSS-49338.1.peg.459 | TRAP-type uncharacterized transport system, fused permease component | | + | + | - | + |
| Amino Acids and Derivatives | Glutamine, glutamate, aspartate, asparagine; ammonia assimilation | Glutamate and Aspartate uptake in Bacteria | Sodium/Proton/glutamate symport protein | | + | + | - | + |
| **Missing only in *Bb*** | | | | | | | | |
| Cell Wall and Capsule | Gram–Negative cell wall components | Lipopolysaccharide assembly | HtrA protease/chaperone protein | | + | - | + | + |
| DNA Metabolism | no subcategory | Restriction–Modification System | Type I restriction–modification system, DNA–methyltransferase subunit M | | + | - | + | + |
| DNA Metabolism | no subcategory | Restriction–Modification System | Type I restriction–modification system, specificity subunit S | | + | - | + | + |
| Iron acquisition and metabolism | no subcategory | Hemin transport system | Outer membrane receptor proteins, mostly Fe transport | | + | - | + | + |
| Membrane Transport | Protein secretion system, Type VI | Type VI secretion systems | Uncharacterized protein ImpD | | + | - | + | + |
| Membrane Transport | no subcategory | Tricarboxylate transport system | Ammonia monooxygenase | | + | - | + | + |
| Miscellaneous | Plant–Prokaryote DOE project | COG0523 | GTP cyclohydrolase I type 1 | | + | - | + | + |
| Phosphorus Metabolism | no subcategory | Phosphate metabolism | Alkaline phosphatase | | + | - | + | + |
| Respiration | no subcategory | Biogenesis of cbb3–type cytochrome c oxidases | Putative analog of CcoH, COG3198 | | + | - | + | + |
| Stress Response | Oxidative stress | Protection from Reactive Oxygen Species | Cytochrome c551 peroxidase | | + | - | + | + |
| Virulence, Disease and Defense | Resistance to antibiotics and toxic compounds | Cobalt–zinc–cadmium resistance | Cation efflux system protein CusA | | + | - | + | + |
| Virulence, Disease and Defense | Resistance to antibiotics and toxic compounds | Cobalt–zinc–cadmium resistance | Cobalt–zinc–cadmium resistance protein CzcA | | + | - | + | + |
| Membrane Transport | Protein and nucleoprotein secretion system, Type IV | Conjugative transfer | IncQ plasmid conjugative transfer protein TraQ (RP4 TrbM homolog) | | + | - | + | + |
| **Missing only in A8** | | | | | | | | |
| Cofactors, Vitamins, Prosthetic Groups, Pigments | Folate and pterines | Folate Biosynthesis | COG0488– ATPase components of ABC transporters with duplicated ATPase domains | | - | + | + | + |
| Cell Division and Cell Cycle | no subcategory | Bacterial Cytoskeleton | Cell division protein FtsB | | - | + | + | + |
| Clustering–based subsystems | no subcategory | CBSS–56780.10.peg.1536 | Metal–dependent hydrolase YbeY, involved in rRNA and/or ribosome maturation and assembly | | - | + | + | + |
| Cofactors, Vitamins, Prosthetic Groups, Pigments | Folate and pterines | Folate Biosynthesis | COG0488– ATPase components of ABC transporters with duplicated ATPase domains | | - | + | + | + |
| Cofactors, Vitamins, Prosthetic Groups, Pigments | Tetrapyrroles | Heme and Siroheme Biosynthesis | Uncharacterized protein EC–HemY, likely associated with heme metabolism based on gene clustering with hemC, hemD in Proteobacteria (unrelated to HemY–type PPO in GramPositives) | | - | + | + | + |
| Amino Acids and Derivatives | Aromatic amino acids and derivatives | Phenylalanine and Tyrosine Branches from Chorismate | Cyclohexadienyl dehydrogenase | | - | + | + | + |
| Protein Metabolism | Protein biosynthesis | Ribosome LSU bacterial | LSU ribosomal protein L17p | | - | + | + | + |
| Clustering–based subsystems | Cell Division | Cell Division Subsystem including YidCD | Protein YidD | | - | + | + | + |

**Table F.** Distribution of major protein degradation genes in *Tetrathiobacter kashmirensis* WT001^T^, *Achromobacter xylosoxidans* A8, *Bordetella bronchiseptica* RB50 and *Taylorella equigenitalis* MCE9**.** The table includes only protein degradation genes of the studied *Alcaligenaceae* genomes which putatively constitute complete pathway variants. +, present; -, absent; P, pseudogene.

| **Metabolic subsystem** | **PEG identified** | **A8** | **RB50** | **WT001^T^** | **MCE9** |
| --- | --- | --- | --- | --- | --- |
| Aminopeptidases | Xaa-Pro aminopeptidase | **+** | **+** | **+** | **+** |
|  | Membrane alanine aminopeptidase N | **+** | **+** | **+** | **+** |
|  | Cytosol aminopeptidase PepA | **+** | **+** | **+** | **+** |
| Putative TldE-TldD proteolytic complex | TldD protein, part of proposed TldE/TldD proteolytic complex | **+** | **+** | **-** | **+** |
|  | TldE/PmbA protein, part of proposed TldE/TldD proteolytic complex | **+** | **+** | **-** | **+** |
|  | FIG138315: Putative alpha helix protein | **+** | **+** | **-** | **+** |
| Protein degradation | Oligopeptidase A | **+** | **+** | **+** | **+** |
|  | Leucyl/phenylalanyl-tRNA--protein transferase | **+** | **+** | **+** | **-** |
|  | Arginine-tRNA-protein transferase | **+** | **+** | **+** | **-** |
|  | Asp-X dipeptidase | **+** | **+** | **+** | **-** |
|  | Aminopeptidase YpdF (MP-, MA-, MS-, AP-, NP- specific) | **+** | **-** | **-** | **+** |
| Dipeptidases | Alpha-aspartyl dipeptidase Peptidase E | **+** | **-** | **-** | **-** |
|  | Aminoacyl-histidine dipeptidase (Peptidase D) | **-** | **-** | **-** | **+** |
| Proteasome bacterial | Uncharacterized protein, similar to the N-terminal domain of Lon protease | **+** | **+** | **+** | **-** |
|  | ATP-dependent Clp protease ATP-binding subunit ClpX | **+** | **+** | **+** | **+** |
|  | ATP-dependent Clp protease proteolytic subunit | **+** | **+** | **+** | **+** |
|  | ATP-dependent hsl protease ATP-binding subunit HslU | **+** | **+** | **+** | **+** |
|  | ATP-dependent protease HslV | **+** | **+** | **+** | **+** |
|  | ATP-dependent protease La Type I | **+** | **+** | **P** | **+** |
| Proteolysis in bacteria, ATP-dependent | DNA repair protein RadA | **+** | **+** | **+** | **+** |
|  | ATP-dependent Clp protease ATP-binding subunit ClpX | **+** | **+** | **+** | **+** |
|  | ClpXP protease specificity-enhancing factor | **+** | **+** | **+** | **-** |
|  | ATP-dependent Clp protease ATP-binding subunit ClpA | **+** | **+** | **+** | **+** |
|  | Outer membrane stress sensor protease DegS | **+** | **+** | **+** | **+** |
|  | ClpB protein | **+** | **+** | **P** | **+** |
|  | ATP-dependent protease HslV | **+** | **+** | **+** | **+** |
|  | ATP-dependent protease La Type I | **+** | **+** | **+** | **+** |
|  | ATP-dependent Clp protease adaptor protein ClpS | **+** | **+** | **+** | **-** |
|  | ATP-dependent Clp protease proteolytic subunit | **+** | **+** | **P** | **+** |
|  | ATP-dependent hsl protease ATP-binding subunit HslU | **+** | **+** | **+** | **+** |
|  | ATPase, AFG1 family | **-** | **-** | **+** | **-** |
| Omega peptidases | Isoaspartyl aminopeptidase | **-** | **+** | **+** | **+** |
| Metallocarboxypeptidases | D-alanyl-D-alanine carboxypeptidase | **-** | **+** | **+** | **+** |
|  | Muramoyltetrapeptide carboxypeptidase | **-** | **+** | **+** | **+** |
|  | Glutamate carboxypeptidase | **-** | **-** | **+** | **-** |

A8, *Bb*, *Tk* and *Te* respectively encompass 29, 30, 29 and 26 PEGs in different protein-degrading subsystems. *Te* despite its small genome size, contains almost all the protein degradation genes that are encountered in the relatively larger genomes. Conversely, all the 26 relevant genes of *Te*, except one Aminoacyl-histidine dipeptidase (Peptidase D), are present in at least one of the other three genomes. As such, A8 and *Te* have two unique dipeptidases viz., alpha-aspartyl dipeptidase (Peptidase E) and aminoacyl-histidine dipeptidase (Peptidase D) respectively, whereas *Tk* and *Bb* have none. All the four genomes encompass three identical aminopeptidases each. *Bb* and *Te* have two metallocarboxypeptidases each while *Tk* has three (the unique being a glutamate carboxypeptidase) and A8 has none. All the genomes, except A8, have a single Omega peptidase viz., isoaspartyl aminopeptidase. Again, all the genomes have five common genes for bacterial proteasomes, while only one uncharacterized protein, similar to the N-terminal domain of Lon protease is found in A8, *Bb* and *Tk*, but not *Te*. Twelve more genes for ATP-dependent proteolysis are also more or less conserved in the family, except for the fact that the ClpXP protease specificity-enhancing factor and Clp protease adaptor protein ClpS are missing in *Te*, and one AFG1 family ATPase is unique to *Tk*.

**Figure A**

**
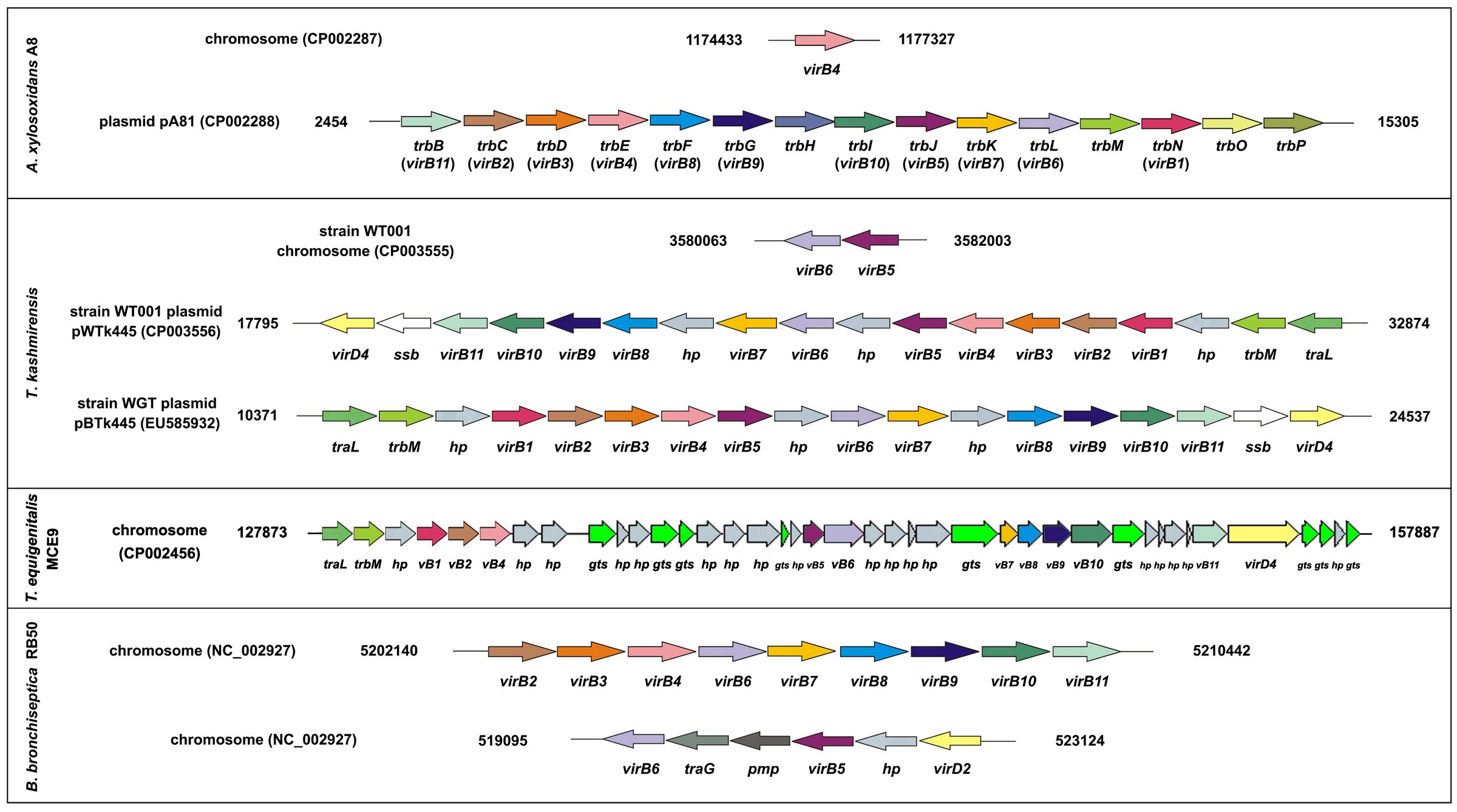
**

**Figure B**

**
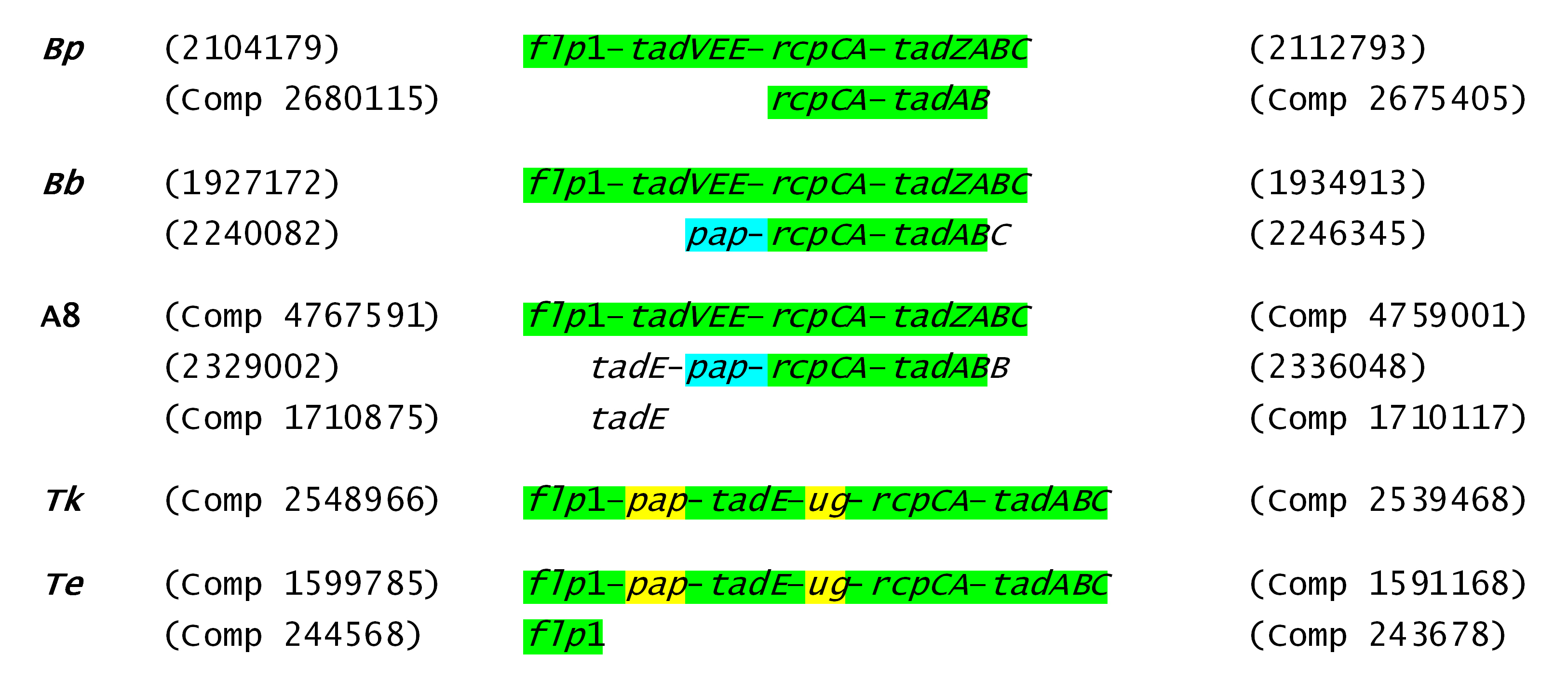
**

**Figure C**

**
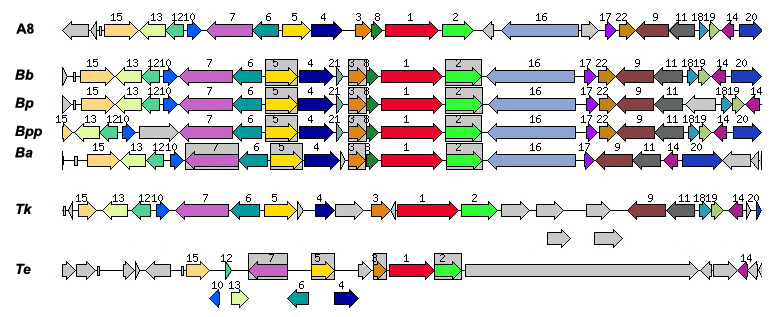
**

**Legends of figures included in this file**

**Figure A –** Genomic context of the Type 4 secretion systems (T4SSs) of the four studied genomes. Numbers in parentheses refer to GenBank accession numbers of the chromosome or plasmid bearing the T4SS. Numbers at the two ends of the gene clusters denote the nucleotide positions from where the clusters start and end. Arrows representing the genes are not proportionate to gene lengths. Arrowheads indicate the transcriptional orientation of the genes. Functionally identical homologs are marked up with same colors. v1 through v10 in T. equigenitalis MCE9 denotes *virB1* through *virB10*, *hp* denotes hypothetical protein-encoding gene, and *gts* stands for Glycyl-tRNA synthetase.

**Figure B –** Genomic context of the tight adherence (Tad) system in *Bb*, *Bp*, A8, *Tk* and *Te*. Numbers in parentheses at the two ends of the gene clusters denote the nucleotide positions from where the clusters start and end. Genome alignments were done manually. All the putative coding sequences in question were characterized individually by BLASTP analysis. ***Bb***, *B. bronchiseptica* RB50; ***Bp*,** *Bordetella pertussis* Tohama I; **A8**, *A. xylosoxidans* A8; ***Tk***, *T*. *kashmirensis* WT001^T^; ***Te***, T. equigenitalis MCE9. The *flp* (fimbrial lowmolecular-weight protein)–*rcp* (rough colony protein)–*tad* genes are collectively known as *tad* genes (Tomich, Planet, and Figurski 2007); *pap* stands for a pilus assembly protein-encoding gene, *ug* denotes an unrelated gene.

**Figure C –** *dnaK* gene cluster and adjacent loci of A8, *Bb*, *Bp*, *Bpp*, *Tk* and *Te*. The *dnaKJ* neighborhood shows significant co-linearity across *Alcaligenaceae*; however the relatively closer relationships between A8 and *Bordetella* spp., and *Tk* and *Te* are also clearly discernible. Functionally identical homologs are marked with same colors and numbers. Genes with conserved relative positions are functionally coupled and share gray background boxes. Genome alignments were done using RAST. **1,** gene for chaperone protein DnaK; **2,** gene for chaperone protein DnaJ; **3,** gene for heat shock protein GrpE. **A8**, *A. xylosoxidans* A8; ***Bb***, *B. bronchiseptica* RB50; ***Bp*,** *Bordetella pertussis* Tohama I; ***Bpp*,** *Bordetella parapertussis* 12822; ***Ba***, *Bordetella avium* 197N; ***Tk***, *T*. *kashmirensis* WT001^T^; ***Te***, T. equigenitalis MCE9.
