# Supplementary material for: Genome Implosion Elicits Host-Confinement in Alcaligenaceae: Evidence from the Comparative Genomics of Tetrathiobacter kashmirensis, a Pathogen in the Making
Source: PLoS One. 2013 May 31;8(5):e64856. doi: 10.1371/journal.pone.0064856 (PMC3669393; doi:10.1371/journal.pone.0064856)
Supplement: File S2 — A tabular comparison of the DNA metabolizing machineries of A8, Bb , Tk and Te has been included in this file. (DOC) [file pone.0064856.s002.doc]

**Comparison of the DNA metabolizing machineries of A8, *Bb*, *Tk* and *Te***

**DNA replication.** All the four genomes encode for α, β, γ, τ**,** δ and δ', and ε subunits of DNA polymerase III, the primary enzyme complex involved in prokaryotic DNA replication. Genes encoding the θ subunit (a core constituent of DNA Pol III which augments the proofreading activity of the ε subunit) [1,2] are, however, missing in all of them, thereby suggesting that these *Alcaligenaceae* could be prone to the incorporation of genome-wide mutations during cell division at intrinsically high rates. Again, out of the χ and ψ subunits (which form a 1:1 complex and bind to γ or τ to increase their affinity for δ.δ' [3]) only the former is present in A8, *Tk* and *Bb*, but not *Te*.

Out of the other four DNA polymerases (viz., Pol I, Pol II, Pol IV, and Pol V, all essentially associated with DNA repair) Pol I is present in all four, whereas Pol IV (thought to be involved in SOS repair and translesion repair [4,5]) is there in A8, *Bb* and *Tk*, but not in *Te*. *Bb* additionally has a RecA/RadA recombinase, a Pol IV-like protein ImuB and an error-prone repair homolog of DNA polymerase III alpha subunit clustered in a single locus.

All the four genomes encompass a copy each of the four genes necessary to synthesize the two subunits each of DNA gyrase and Topoisomerase IV (both ATP-dependent Type IIA DNA topoisomerases), even as the genes for DNA gyrase subunit B and the DNA topoisomerase IV subunit A are pseudogenized in *Tk*. In addition to these all four have one ATP-independent, Type I DNA topoisomerases (viz., DNA topoisomerase III), plus a SWIB-domain-containing protein that is highly conserved among all *Alcaligenaceae*. Notably, the SWIB/MDM2 domain family has been implicated in chromatin remodelling in eukaryotes [6,7,8]. Pseudogenization of Topoisomerase IV in *Tk* is most likely to have adverse effects on its DNA replication and transcription functions since relevant protein factors may not properly bind with its DNA amidst anomalous opening/relaxation of the supercoiled genome. How *Tk* circumvents this constraint is worth investigating in the coming days.

**DNA recombination and repair.** There are two major homologous recombination mechanisms in bacteria, viz; the RecBCD and RecF/RecFOR pathways. RecBCD, also known as Exonuclease V, initiates recombinational repair from potentially lethal double strand breaks in DNA which may result from ionizing radiation, replication errors, endonuclease actions, oxidative damage or several other factors [9]. The RecBCD complex acts both as a helicase that unwinds or separates the DNA strands and also a nuclease that makes single stranded nicks in DNA [10]. Interestingly, genes for all the subunits of RecBCD are absent in all the compared genomes. However, an AddAB complex, which can potentially compensate this loss by virtue of its ability to repair double strand breaks in the DNA [11], present in A8, *Tk* and *Bb* but not *Te*.

The RecF/RecFOR pathway, (governed by RecA, RecF, RecJ, RecN, RecO, RecQ, RecR, RecG and one single strand binding proteins) [12] on the other hand, generally repairs single strand gaps via homologous recombination and additionally takes care of double strand breaks when the RecBCD pathway is inactivated by mutations [13]. The A8 and *Bb* genomes encode for all the aforesaid components of the RecF/RecFOR pathway. *Te*, on the other hand, lacks the *recQ* gene, while *Tk* has its *recO* and *recN* pseudogenized (see Table below). RecQ helicase participates in plasmid recombination as well as repair of damages inflicted by UV radiation, free radicals, alkylating agents and replication errors [14,15]. In view of the fact that *Te* has neither RecBCD nor AddAB its want of RecQ may just translate into complete abolition of both single and double strand break repair via homologous recombination. RecO facilitates the loading of RecA upon single strand breaks in bacteria having intact RecFOR systems as well as those having a truncated version wanting *recF* (the RecOR pathway, notably all the studied genomes fall under the second category) [16]. As such, pseudogenization of *recO*, or for that matter *recN* also, in *Tk* may have rendered its RecOR pathway virtually defunct. Thus *Tk* only retains the option for double stranded DNA repair by virtue of its AddAB system, but other recombinatorial DNA repair mechanisms including those involving single stranded DNA might be seriously jeopardized because RecO-mediated RecA loading is central to all such processes (Since recBCD is also absent). As a corollary to this it seems quite likely that *Tk* has critically compromised its ability to acquire any incoming ssDNA via homologous recombination.

A8, *Bb* and *Tk* but not *Te* have all the components of the low-fidelity non-homologous end-joining repair pathway (see Table below) that works via double strand break repair proteins when homologous template is missing for recombinatorial repair [17]. Similarly, the Ada-AlkB pathway, a semiconserved response against alkylating agents [18,19], is wanting only in the *Te* genome. Notably however mechanisms for the regulation of SOS repair and the RuvABC holiday junction complex formation are intact in all the four *Alcaligenaceae*.

The DNA mismatch repair system is governed by MutH, MutS, MutL and the ATP-dependent helicase UvrD/PcrA. MutS recognizes the site of mismatch at GATC (where the adenine is methylated) with the help of DAM methylase and then recruits MutL, which in its turn loads the endonucleaase MutH [20,21]. Like several other bacteria, *mutH* is absent in all the four organisms, but this disability, as suggested elsewhere [22,23], may be bypassed using methylation-independent mechanisms. Anyhow, the *Tk* Mut system is unlikely to work properly since *mutS*, *mutL*, and its adjacent *uvrD*/*pcrA*, are all pseudogenized in this organism (see Table below). These features in tandem with the absence of the θ subunit of DNA polymerase III are strong indicators of especially high mutability of the *Tk* genome.

All the compared genomes except *Bb* have one or the other component of the DNA base excision repair system omitted or pseudogenized (see Table below). In contrast, complete nucleotide excision repair systems (excinuclease UvrABC) are present in A8, *Bb* and *Te*, whereas in *Tk* *uvrA* and *uvrC* appears to have been pseudogenized. Potential disruption of this high fidelity repair system only adds to *Tk*’s existing vulnerability against drug- and/or UV-induced mutagenesis. On top of this, RadA/Sms (which renders recombination-based repair by disallowing possible branching of the DNA molecules by blocking the replication fork) [24] are further pseudogenized in *Tk* even as they are present in A8, *Bb* and *Te*. It is however noteworthy that *Tk* exclusively possess the SbcCD complex-encoding genes (see Table below) which have been shown to play crucial roles in genome repair and maintenance in *Deinococcus* [25,26]. How this system in *Tk* compensates for other shortcomings would be interesting to check in the future.

Anyway, contrary to all the negative trends mentioned above, *Tk* and *Te* exclusively possess a copy each of co-transcribed *umuD* (encoding the error-prone DNA repair protein UmuD) and *umuC* (encoding error-prone lesion bypass DNA polymerase V) homologs. In case of *Te*, *umuDC* is located in the chromosome alongside the only DNA helicase IV (ATP-dependent DNA helicase UvrD/PcrA) present in its genome. But in *Tk*, the pair is plasmid borne and not associated with any *uvrD*. There are, nevertheless, two *uvrD* homologs in the *Tk* chromosome, out of which one is a pseudogene and the other is clustered with a Pol IV-encoding gene. Even though A8 and *Bb* have no *umu* homolog, they respectively have two and four discrete *uvrD*-like genes in their chromosomes (see Table below).

**DNA restriction systems.** The A8 and *Te* genomes encode all the three polypeptide components of the Type I restriction-modification system, viz., the DNA-methyltransferase subunit M, restriction subunit R and the specificity subunit S, from a single operon. In contrast, *Tk* encodes only the M and S subunits, while *Bb* encodes none (see Table below). A8 additionally has two stray copies each of N-6 adenine-specific DNA methylase and methylation-requiring restriction (Mrr) system endonuclease. *Tk*, in its turn, has a stray gene for N-6 adenine-specific DNA-methyltransferase, plus an Mrr system endonuclease and a putative restriction endonuclease belonging to the HNH family of small DNA binding and digestion proteins (that include pyocins, colicins and anaredoxins of bacteria [27] as well as viruses [28]) characterized by two His and one Asn residues. The lack of a Type I system notwithstanding, *Bb* does possess a Type II and a Type III restriction modification system, located in two discrete loci of the genome. Both the systems lack specificity proteins and are comprised of the R and M proteins only [29].

**Table.** Distribution of genes putatively involved in DNA recombination and repair in *Tetrathiobacter kashmirensis* WT001^T^, *Achromobacter xylosoxidans* A8, *Bordetella bronchiseptica* RB50 and *Taylorella equigenitalis* MCE9**.** +, present; -, absent; P, pseudogene.

| **Recombination and / or repair systems** | **PEGs** | **A8** | **WT001^T^** | **RB50** | **MCE9** |
| --- | --- | --- | --- | --- | --- |
| RecFOR recombination pathway | *recJ* | + | + | + | + |
|  | *recN* | + | P | + | + |
|  | *recO* | + | P | + | + |
|  | *recQ* | + | + | + | - |
|  | *recR* | + | + | + | + |
|  | Single-stranded  DNA-binding protein | + | + | + | + |
| RuvABC Holliday junction complex | *ruvA* | + | + | + | + |
|  | *ruvB* | + | + | + | + |
|  | *ruvC* | + | + | + | + |
|  | *yebC* | - | - | + | - |
| Nucleotide excision repair | *uvrA* | + | P | + | + |
|  | *uvrB* | + | + | + | + |
|  | *uvrC* | + | P | + | + |
|  | *uvrD/pcrA* | + | P | + | + |
| SOS response | *lexA* | + | + | + | + |
|  | *recA* | + | + | + | + |
|  | *recX* | + | + | + | + |
| RecBCD pathway | *recB* | - | - | - | - |
|  | *recC* | - | - | - | - |
|  | *recD* | - | - | - | - |
| Mismatch repair | *mutH* | - | - | - | - |
|  | *mutL* | + | P | + | + |
|  | *mutS1* | + | P | + | + |
|  | A/G-specific adenine glycosylase | + | + | + | - |
|  | *uvrD/pcrA* | + | P | + | + |
| Non-homologous end-joining, bacterial type | Ku | + | + | + | - |
|  | PE (DNA ligaseD) | + | + | + | - |
|  | *ligD*, ligase domain | + | + | + | - |
|  | *ligD*, polymerase domain | + | + | + | - |
|  | ATP-dependent DNA ligase clustered with Ku protein, LigD | + | + | + | - |
| AddAB pathway | *addA* | + | + | + | - |
|  | *addB* | + | + | + | - |
| Other recombination repair protein | *radA/sms* | + | P | + | + |
|  | *radC* RNA motif | + | + | + | + |
| RmuC recombination pathway | *rmuC* | + | P | + | + |
| SbcDC pathway | *sbcC* (*rmuA*) | - | + | - | - |
|  | *sbcD* | - | + | - | - |
| UmuDC pathway | *umuD* | - | + | - | + |
|  | *umuC* | - | + | - | + |
| DNA Repair Base Excision | Exonuclease III/AP Endonuclease | + | + | + | - |
|  | DNA ligase | + | + | + | + |
|  | DNA polymerase I | + | + | + | + |
|  | Endonuclease III | + | + | + | + |
|  | Uracil-DNA glycosylase, family 1 | + | + | + | + |
|  | DNA-3-methyladenine glycosylase | + | + | + | - |
|  | Formamidopyrimidine-DNA glycosylase | + | P | + | - |
|  | DNA-3-methyladenine glycosylase II | - | + | + | - |
|  | Uracil-DNA glycosylase, family 4 | + | + | + | - |
| Ada-alkB  pathway | Alkylated DNA repair protein AlkB | + | + | + | - |
|  | ADA regulatory protein | + | + | + | - |
| UvrD-like Helicase, ATP-binding domain and other related helicases | ATP-dependent DNA helicase Rep | + | + | + | - |
|  | DinG family ATP-dependent helicase YoaA | + | + | + | - |
|  | DinG family ATP-dependent helicase CPE1197 | + | - | - | - |
|  | DNA helicase IV (UvrD/REP helicase) | - | P | - | + |
| Type I restriction-modification system, specificity subunit S | DNA-methyltransferase subunit M | + | + | - | + |
|  | Specificity subunit S | + | + | - | + |
|  | Restriction subunit R | + | - | - | + |
| Type II restriction-modification system | Type II restriction endonuclease | - | - | + | - |
|  | DNA methylase/  methyltransferase | - | - | + | - |
| Type III restriction-modification system | type III restriction enzyme | - | - | + | - |
|  | Type III restriction-modification system methylation subunit | - | - | + | - |
| Other deoxyribonucleases | Exodeoxyribonuclease VII small subunit/xseB/yajE | + | + | + | - |
|  | DNA polymerase IV / DinB / DinP | + | + | + | - |
|  | DNA polymerase-like protein PA0670 | + | - | + | - |

**References**

1. Kelman Z, O'Donnell M (1995) DNA polymerase III holoenzyme: structure and function of a chromosomal replicating machine. Annu Rev Biochem 64: 171-200.

2. Taft-Benz SA, Schaaper RM (2004) The theta subunit of Escherichia coli DNA polymerase III: a role in stabilizing the epsilon proofreading subunit. J Bacteriol 186: 2774-2780.

3. Olson MW, Dallmann HG, McHenry CS (1995) DnaX complex of Escherichia coli DNA polymerase III holoenzyme. The chi psi complex functions by increasing the affinity of tau and gamma for delta.delta' to a physiologically relevant range. J Biol Chem 270: 29570-29577.

4. Cirz RT, Chin JK, Andes DR, de Crecy-Lagard V, Craig WA, et al. (2005) Inhibition of mutation and combating the evolution of antibiotic resistance. PLoS Biol 3: e176.

5. Napolitano R, Janel-Bintz R, Wagner J, Fuchs RP (2000) All three SOS-inducible DNA polymerases (Pol II, Pol IV and Pol V) are involved in induced mutagenesis. Embo J 19: 6259-6265.

6. Whitehouse I, Flaus A, Cairns BR, White MF, Workman JL, et al. (1999) Nucleosome mobilization catalysed by the yeast SWI/SNF complex. Nature 400: 784-787.

7. Zofall M, Persinger J, Kassabov SR, Bartholomew B (2006) Chromatin remodeling by ISW2 and SWI/SNF requires DNA translocation inside the nucleosome. Nat Struct Mol Biol 13: 339-346.

8. Bennett-Lovsey R, Hart SE, Shirai H, Mizuguchi K (2002) The SWIB and the MDM2 domains are homologous and share a common fold. Bioinformatics 18: 626-630.

9. Smith GR (2012) How RecBCD enzyme and Chi promote DNA break repair and recombination: a molecular biologist's view. Microbiol Mol Biol Rev 76: 217-228.

10. Singleton MR, Dillingham MS, Gaudier M, Kowalczykowski SC, Wigley DB (2004) Crystal structure of RecBCD enzyme reveals a machine for processing DNA breaks. Nature 432: 187-193.

11. Yeeles JT, Dillingham MS (2010) The processing of double-stranded DNA breaks for recombinational repair by helicase-nuclease complexes. DNA Repair (Amst) 9: 276-285.

12. Kowalczykowski SC, Dixon DA, Eggleston AK, Lauder SD, Rehrauer WM (1994) Biochemistry of homologous recombination in Escherichia coli. Microbiol Rev 58: 401-465.

13. Morimatsu K, Kowalczykowski SC (2003) RecFOR proteins load RecA protein onto gapped DNA to accelerate DNA strand exchange: a universal step of recombinational repair. Mol Cell 11: 1337-1347.

14. Cobb JA, Bjergbaek L, Gasser SM (2002) RecQ helicases: at the heart of genetic stability. FEBS Lett 529: 43-48.

15. Kaneko H, Fukao T, Kondo N (2004) The function of RecQ helicase gene family (especially BLM) in DNA recombination and joining. Adv Biophys 38: 45-64.

16. Manfredi C, Carrasco B, Ayora S, Alonso JC (2008) Bacillus subtilis RecO nucleates RecA onto SsbA-coated single-stranded DNA. J Biol Chem 283: 24837-24847.

17. Bowater R, Doherty AJ (2006) Making ends meet: repairing breaks in bacterial DNA by non-homologous end-joining. PLoS Genet 2: e8.

18. Shevell DE, Friedman BM, Walker GC (1990) Resistance to alkylation damage in Escherichia coli: role of the Ada protein in induction of the adaptive response. Mutat Res 233: 53-72.

19. Volkert MR (1988) Adaptive response of Escherichia coli to alkylation damage. Environ Mol Mutagen 11: 241-255.

20. Kunkel TA, Erie DA (2005) DNA mismatch repair. Annu Rev Biochem 74: 681-710.

21. Schofield MJ, Hsieh P (2003) DNA mismatch repair: molecular mechanisms and biological function. Annu Rev Microbiol 57: 579-608.

22. Dreiseikelmann B, Wackernagel W (1981) Absence in Bacillus subtilis and Staphylococcus aureus of the sequence-specific deoxyribonucleic acid methylation that is conferred in Escherichia coli K-12 by the dam and dcm enzymes. J Bacteriol 147: 259-261.

23. Pillon MC, Lorenowicz JJ, Uckelmann M, Klocko AD, Mitchell RR, et al. Structure of the endonuclease domain of MutL: unlicensed to cut. Mol Cell 39: 145-151.

24. Beam CE, Saveson CJ, Lovett ST (2002) Role for radA/sms in recombination intermediate processing in Escherichia coli. J Bacteriol 184: 6836-6844.

25. Kamble VA, Misra HS The SbcCD complex of Deinococcus radiodurans contributes to radioresistance and DNA strand break repair in vivo and exhibits Mre11-Rad50 type activity in vitro. DNA Repair (Amst) 9: 488-494.

26. Hu Y, Tian B, Xu G, Yin L, Hua X, et al. Characteristics of nuclease activity of the SbcCD complex from Deinococcus radiodurans. J Biochem 147: 307-315.

27. Pommer AJ, Cal S, Keeble AH, Walker D, Evans SJ, et al. (2001) Mechanism and cleavage specificity of the H-N-H endonuclease colicin E9. J Mol Biol 314: 735-749.

28. Moodley S, Maxwell KL, Kanelis V (2012) The protein gp74 from the bacteriophage HK97 functions as a HNH endonuclease. Protein Sci 21: 809-818.

29. Wilson GG, Murray NE (1991) Restriction and modification systems. Annu Rev Genet 25: 585-627.
