# Supplementary material for: Genome Implosion Elicits Host-Confinement in Alcaligenaceae: Evidence from the Comparative Genomics of Tetrathiobacter kashmirensis, a Pathogen in the Making
Source: PLoS One. 2013 May 31;8(5):e64856. doi: 10.1371/journal.pone.0064856 (PMC3669393; doi:10.1371/journal.pone.0064856)
Supplement: File S3 — This file includes a brief comparative genomic study of the secretion systems of the four Alcaligenaceae members in question. (DOC) [file pone.0064856.s003.doc]

**Comparative genomics of the secretion systems of the four *Alcaligenaceae***

Several secretion systems established as virulence factors in pathogenic bacteria were found to be ubiquitous in *Alcaligenaceae*. These include the Tad macromolecular transport system [a subtype of the Type II secretion (T2SS)], T4SS, several complements of T5SS autotransporters, Type VI secretion system (T6SS), and Type VII secretion system (T7SS) or the Chaperone-Usher pathway.

The T2SS, in general, has been accredited with the export of proteins into target host cells as well as to the extracellular milieu, thereby promoting virulence and/or environmental fitness of the concerned organism [1]. The Tad system, in its turn, is a subtype of T2SS encountered in many bacterial and archaeal species. The *tad* genes, essential for biofilm formation, colonization and pathogenesis in *Aggregatibacter*, *Haemophilus*, *Pasteurella*, *Pseudomonas*, *Yersinia*, *Caulobacter*, *Vibrio*, *Mycobacterium* etc., are organized on the so-called widespread colonization island (WCI) and govern the assembly of adhesive fimbrial low-molecular-weight protein (Flp) pili [2]. Essentially syntenic and orthologous *tad* loci could be identified in all the compared genomes. A8 and *Bb* has two major *tad* clusters, whereas *Tk* and *Te* have one apiece. Gene organization in the two discrete clusters of A8 is more comparable with the corresponding loci of *Bb*. On the other hand, co-linearity of genes in the solitary *tad* loci of *Tk* and *Te* resemble each other more. Besides the *tad* loci, genes (*gspCGHIJKLMN*–two unrelated genes–*gspEDF*) for another T2SS homolog, the general secretion pathway, were clustered in the A8 genome alone between nucleotide positions complement 2854744 to complement 2843060.

The T4SS, which is homologous to the bacterial conjugation machinery [3], is also recognized in *Bordetella* [4,5], *Helicobacter* [6], *Legionella* [7] etc. exporting various toxic proteins to their respective eukaryotic hosts. The prototypical VirB system, again, is renowned for introducing T-DNA of the Ti plasmid of *Agrobacterium tumefaciens* into plant hosts [8]. T4SS homologs are ubiquitous in *Alcaligenaceae*, with the relevant operons being chromosomal in *Te* and *Bb* but borne on the plasmids pWTk445 and pA81 in *Tk* and A8 respectively (Figure 4). This together with the characteristics like presence of paralogous copies of T4SS constituents and partitioning of the system in more than one locus in many *Alcaligenaceae*, occurrence of the *Te* T4SS within the ~72 Kb translocated segment of the genome etc., collectively reiterate the universal mosaic nature of T4SSs and also point towards the key role of genome reorganization in *Alcaligenaceae* evolution. While bordetellae are known to use T4SS (the *ptl* system) to secrete pertussis toxin [4,5], the functional roles of the A8 and *Te* homologs are not yet clear. As for the *Tk* T4SS, the copy borne on pBTk445 has already been appreciated for its involvement in conjugative DNA transfer [9]. But the potential of *Tk* homologs to transfer additional substances can not be ruled out completely. The presence of a *virD4* gene (reportedly essential for the transfer of nucleoprotein particles by pathogenic T4SSs [6]) next to the T4SS in both pWTk445 and pBTk445 fuels such speculations. It is again interesting to note that the organization of T4SS genes in pA81 is totally distinct from the arrangement in *Tk*, *Te* or the main locus of *Bb* where a conserved synteny of these genes is perceptible after discounting cases of insertions / deletions in-between. The last three systems thus seem to share a common evolutionary history, which is distinct from that of the system on pA81. Corroboratively, most of the constituents of these three interrelated systems formed consistent phylogenetic clusters with homologs from *Vibrio*, *Xanthomonas*, *Collimonas*, *Brucella*, *Neisseria*, *Kingella* etc. (data not shown). Moreover, seven putative T4SS proteins of *Te* (VirB2, 4, 5, 6, 7, 8, 9, plus VirD4) showed highest identity with *Tk* homologs, at the same time as four *Tk* homologs (VirB2, 3, 5, 9, plus VirD4) were found closest to *Te* counterparts. In contrast, gene products of the second *Bb* cluster showed 96-98% identity with homologs from *Ax* strain C54, and constituents of the A8 system exhibited 70-100% identity with homologs from *Comamonas*, *Enterobacter*, *Delftia*, *Aeromonas* etc.

Several complements of T5SS autotransporters could be identified in the genomes of A8, *Bb* and *Tk*, but none in *Te*. *Bb* was already known to have the capacity to express 20 putative or functional autotransporters [10]. In contrast, 10 and 2 autotransporter PEGs were detectable in A8 and *Tk* respectively, besides which *Tk* contained two subtilisin-like serine protease genes while A8 had none.

The T6SS, first identified in *Vibrio cholerae* [11] and *Pseudomonas aeruginosa* [12], is now known to be ubiquitous in ecologically diverse *Proteobacteria* rendering roles as diverse as pathogenesis, defense against simple eukaryotic predators and inter-bacterial interaction [13]. At least 13 T6SS genes, including those encoding the universally secreted substrates Hcp1 and VgrG, are conserved in A8, *Bb*, *Tk* and *Te*.

The T7SS renders the secretion and assembly of prepilin proteins for the biogenesis of diverse types of bacterial pili or fimbriae (including the prototypical Type 1 pili), which play central roles in conjugation, adherence, twitching motility, biofilm formation or immunomodulation [14]. T7SSs involve the PapD chaperone that caps the pilus subunits in the periplasm, and the outer membrane PapC usher that receives the subunits and serves as an assembly platform [14]. As such, *Tk* has a T7SS locus between nucleotide positions complement 1370158 and complement 1365171 encompassing genes for the P pilus assembly protein FimA, PapD, PapC and a Fimbria adhesin protein. In addition to these, *Tk* has an orphaned PapC-encoding gene (TKWG_07470) plus another P pilus assembly locus between nucleotide positions 1262517 and 1267343 where the usher PEG has been pseudogenized. On the other hand, A8 and Bb respectively have two and one T7SS loci plus quite a few orphaned components, while *Te* is totally devoid of this system.

**References**

1. Cianciotto NP (2005) Type II secretion: a protein secretion system for all seasons. Trends Microbiol 13: 581-588.

2. Tomich M, Planet PJ, Figurski DH (2007) The tad locus: postcards from the widespread colonization island. Nat Rev Microbiol 5: 363-375.

3. Christie PJ (2001) Type IV secretion: intercellular transfer of macromolecules by systems ancestrally related to conjugation machines. Mol Microbiol 40: 294-305.

4. Weiss AA, Johnson FD, Burns DL (1993) Molecular characterization of an operon required for pertussis toxin secretion. Proc Natl Acad Sci U S A 90: 2970-2974.

5. Covacci A, Rappuoli R (1993) Pertussis toxin export requires accessory genes located downstream from the pertussis toxin operon. Mol Microbiol 8: 429-434.

6. Covacci A, Telford JL, Del Giudice G, Parsonnet J, Rappuoli R (1999) Helicobacter pylori virulence and genetic geography. Science 284: 1328-1333.

7. Cascales E, Christie PJ (2003) The versatile bacterial type IV secretion systems. Nat Rev Microbiol 1: 137-149.

8. Christie PJ (1997) Agrobacterium tumefaciens T-complex transport apparatus: a paradigm for a new family of multifunctional transporters in eubacteria. J Bacteriol 179: 3085-3094.

9. Dam B, Ghosh W, Das Gupta SK (2009) Conjugative Type 4 secretion system of a novel large plasmid from the chemoautotroph Tetrathiobacter kashmirensis and construction of shuttle vectors for Alcaligenaceae. Appl Environ Microbiol 75: 4362-4373.

10. Henderson IR, Navarro-Garcia F, Desvaux M, Fernandez RC, Ala'Aldeen D (2004) Type V protein secretion pathway: the autotransporter story. Microbiol Mol Biol Rev 68: 692-744.

11. Pukatzki S, Ma AT, Sturtevant D, Krastins B, Sarracino D, et al. (2006) Identification of a conserved bacterial protein secretion system in Vibrio cholerae using the Dictyostelium host model system. Proc Natl Acad Sci U S A 103: 1528-1533.

12. Mougous JD, Cuff ME, Raunser S, Shen A, Zhou M, et al. (2006) A virulence locus of Pseudomonas aeruginosa encodes a protein secretion apparatus. Science 312: 1526-1530.

13. Schwarz S, Hood RD, Mougous JD (2010) What is type VI secretion doing in all those bugs? Trends Microbiol 18: 531-537.

14. Jacob-Dubuisson F, Kuehn M, Hultgren SJ (1993) A novel secretion apparatus for the assembly of adhesive bacterial pili. Trends Microbiol 1: 50-55.
