# Supplementary material for: Genome Implosion Elicits Host-Confinement in Alcaligenaceae: Evidence from the Comparative Genomics of Tetrathiobacter kashmirensis, a Pathogen in the Making
Source: PLoS One. 2013 May 31;8(5):e64856. doi: 10.1371/journal.pone.0064856 (PMC3669393; doi:10.1371/journal.pone.0064856)
Supplement: File S4 — Comparison of genes governing the development of lipopolysaccharide envelops and surface antigens in the studied Alcaligenaceae . (DOC) [file pone.0064856.s004.doc]

**Cellular envelopes of *Alcaligenaceae* are typically well developed**

The lipopolysaccharide (LPS) envelop of most Gram negative bacteria consists of a lipid A domain, a relatively conserved core oligosaccharide, and a highly variable O-antigen polysaccharide. Pulmonary surfactant proteins (of mammalian hosts) like SP-A and SP-D respectively bind to (and destroy) the lipid A portion of the LPS and the core oligosaccharide of invading bacteria [1,2]. *Bordetella* species have typical lipid A regions linked to branched core oligosaccharides by single ketodeoxyoctulosonic acid (Kdo) residues. Many of these species are resistant to SP-A and SP-D binding [2] by virtue of a typical trisaccharide consisting of α-*N*-acetylglucosamine (GlcNAc), β-2-acetamido-3-acetamido-2,3-dideoxy-mannuronic acid (diNAcManA), and β-l-2-acetamido-4-methylamino-fucose (FucNAcMe) [2] whose biosynthesis and addition to lipid A is controlled by the 12-gene *wlb* operon [3,4]. One WaaF protein, again, adds a heptose residue to the Kdo, and its mutation not only produces a truncated core but also leads to non-expression of the *Te*rminal trisaccharide [5]. The LPS of *Bb* is similar to that of *Bp*, except for the fact that it contains an additional repeating O-antigen structure [comprised of a homopolymer of 2,3-dideoxy-2,3-di-*N*-acetylgalactosaminuronic acid (2,3-di-NAcGalA)] synthesized by the *wbm* operon [6]. The *wlb* operon in *Bb* not only directs the synthesis of the *Te*rminal trisaccharide, but also plays a vital role in the expression of the O antigen [4]. *Bb* further possesses a *pagP* gene, whose protein product palmitoyl transferase adds a palmitate to lipid A [7]. The *pagP* homolog of *Bp* has an insertion sequence element in its promoter region, and is hence inactive [7]. Notably however, neither the O antigen nor the palmitoylated lipid A of *Bb* has any direct role in SP-A/SP-D resistance [2]. Nevertheless, O-antigen structures are extremely diversified in Gram-negative bacteria and apparently have crucial involvement in wide ranging host interactions including enhancement of virulence [8]. Their occurrence in *Bb*, or for that matter in *Bpp* (Table 1 below), could be one of the reasons why these two bordetellae are able to colonize wider range of hosts than *Bp*, which plausibly lost the *wbm* locus by degenerative evolution.

In addition to their typical O-antigen biosynthetic *wbm* loci, *Bb* and Bpp both have one more locus that putatively encodes a lipopolysaccharide O-antigen (Table 2 below). The two loci have several common genes which encode proteins belonging to identical protein families. Almost all sequenced *Alcaligenaceae* genomes possess multiple homologs of many a component of these two loci. But intact orthologs of the *wbm* locus does not occur outside *Bb* and *Bpp*. Remarkably, however, a few distantly related homologs are found clustered together in the genomes of the soil isolate *Achromobacter piechaudii* strain HLE as well as the two *A*. *xylosoxidans* respiratory tract isolates AXX-A and C54, but not the soil isolate A8. It is further noteworthy that percentage G+C contents of most of the *wbm* genes of *Bb* and *Bpp* have ~15% deviation from the respective genomic averages. This observation, together with the rare codon usage patterns encountered in these genes, indicates that the *wbm* locus as such could be a specific foreign acquisition of the mother bordetellar genome. In contrast, complete orthologs of the second O-antigen locus occur in all *Bordetella*, *Achromobacter* and *Tetrathiobacter*, but not *Taylorella*. These examples are tabulated below, wherefrom it is further evident that the *Tk* counterpart has cut back the number of copies of glycosyltransferase, while *Te* has apparently lost the entire locus save one rudimentary ortholog of UDP-glucose dehydrogenase. It is not clear whether the second O-antigen biosynthetic locus can supplement the want of the first (*wbm*) in A8, *Tk* or *Bp*. But the conserved nature of the second locus, together with the ubiquity of homologs of the first, hint at an ancestral origin of O-antigen biosynthesis in *Alcaligenaceae* as a whole. Signatures of overall conservation notwithstanding, trends of pseudogenization and/or wider divergence of these genes are clearly discernible in such members of the family which are either precisely selected to interact with specific hosts (e.g., *Te* and *Bp*) or virtually do not need to interact with any host in their natural habitat (e.g., *Tk* and A8).

Again, homologs of the components of the bordetellar *wlb*-*waa* gene clusters (see Table 3 below) are abundant (and clustered in several discrete loci) in the genomes of all *Alcaligenaceae*. Notably, related genes from the soil isolate A8 have relatively lower levels of identity with their bordetellar counterparts and those from the pathogenic *Ax* strain C54 have much higher identities. *Tk* also has many of these genes but in terms of sequence Identity its homologs are widely diverged from their bordetellar counterparts. Another conspicuous capsular polysaccharide biosynthesis and export locus encoding the Wcb, Wbp and Kps proteins is present in the *Bordetella* genomes (see Table 4 below). Homologs of the components of this cluster are also ubiquitous in *Alcaligenaceae*, even though they are not always co-localized in the concerned genomes. This again reiterates the general propensity of this family for elaborate cellular envelops and capsular structures.

**Table 1.** Genes in the *wbm* O-antigen biosynthetic locus of Bb (between nucleotide positions 123284 and 155070) and their nearest homologs in closely related genomes. Potential orthologs are in blue font. Similar (having only low levels of identity) but phylogenetically diverged homologs (of apparently distinct origin and evolutionary history) are in magenta.

| ***Bb* RB50** | | ***Bpp* 12822** | | ***Bp* Tohama I** | | ***Achromobacter* spp.** | | ***Tk* WT001** | | ***Te* MCE9** | |
| --- | --- | --- | --- | --- | --- | --- | --- | --- | --- | --- | --- |
| **Protein_id** | **Name of the PEG** | **Protein_id** | **Name of the PEG**  **(% identity)** | **Protein_id** | **Name of the PEG**  **(% identity)** | **Species & strain**  **(Protein_id)** | **Name of the PEG**  **(% identity)** | **Protein_id** | **Name of the PEG**  **(% identity)** | **Protein_id** | **Name of the PEG**  **(% identity)** |
| NP_886673 | NAD dependent epimerase/dehydratase | NP_882483 | NAD dependent epimerase/dehydratase (100%) | NP_881714 | *wbpP* gene product / UDP-N-acetylglucosamine C4 epimerase (37%) | Ax C54  (EFV82082)  Ax AXX-A  (EGP47101)  Ax A8  (ADP18914)  *A*. *piechaudii* HLE  (EJO29515) | NAD dependent epimerase/dehydratase (90%)  NAD dependent epimerase/dehydratase (37%)  VI polysaccharide biosynthesis gene VipB/TviC (34%)  VI polysaccharide biosynthesis gene VipB/TviC (34%) | AFK63837 | UDP-N-acetylglucosamine C4 epimerase (34%) | ADU91629 | dTDP-glucose 4,6-dehydratase (25%) |
| NP_886674 | DegT/DnrJ/EryC1/StrS aminotransferase | NP_882484 | DegT/DnrJ/EryC1/StrS aminotransferase  (99%) | NP_878990 | *bplF* gene product  [Ortholog of the *bplF* genes of *Bb* (NP_886702) and *Bpp* (NP_882512)] (38%) | Ax C54  (EFV82097)  Ax AXX-A  (EGP46654)  *A*. *piechaudii* HLE  (EJO31103)  Ax A8  (ADP19581) | Hypothetical gene  (47%)  Lipopolysaccharide biosynthesis gene (35%)  Lipopolysaccharide biosynthesis gene (33%)  Pleiotropic regulator  (32%) | NIL | NIL | ADU91943 | Bacillosamine / Legionaminic acid biosynthesis aminotransferase  PglE; 4-keto-6-deoxy-N-Acetyl-D-hexosaminyl-(Lipid carrier)  Aminotransferase (34%) |
| NP_886675 | Asparagine synthetase | NP_882485 | Asparagine synthetase  (79%) | NP_881711 | Asparagine synthase (32%) | Ax C54  (EFV82083)  Ax A8  (ADP18911)  Ax AXX-A  (EGP47420)  *A*. *piechaudii* HLE  (EJO29518) | Asparagine synthetase  (69%)  Asparagine synthase (glutamine-hydrolyzing) (31%)  Asparagine synthase (32%)  Asparagine synthase (32%) | AFK63834 | Asparagine synthase (31%) | ADU91940 | Asparagine synthase (34%) |
| NP_886676 | UDP-glucose 4-epimerase | NP_882501 | *wbmF* gene product (25%) | NP_879756 | ADP-L-glycero-D-manno-heptose-6-epimerase (24%) | *A*. *piechaudii* HLE  (EJO30028)  Ax AXX-A  (EGP48369)  Ax C54  (EFV84034)  Ax A8  (ADP17803) | GDP-L-fucose synthase (26%)  GDP-L-fucose synthase (26%)  NAD-dependent epimerase / dehydratase (26%)  GDP-L-fucose synthase (26%) | AFK62378 | ADP-L-glycero-D-manno-heptose-6-epimerase (23%) | ADU91629 | dTDP-glucose 4,6-dehydratase (24%) |
| NP_886677 | Phosphoesterase | NIL | NIL | NIL | NIL | Ax C54  (EFV84259)  Ax A8  *A*. *piechaudii* HLE  Ax AXX-A | Hypothetical gene (32%)  NIL  NIL  NIL | NIL | NIL | NIL | NIL |
| NP_886678 | Carbamoyl phosphate synthase-like gene | NP_883545 | Carbamoyl-phosphate synthase large chain (25%) | NP_880195 | Carbamoyl-phosphate synthase large chain (25%) | Ax C54 (EFV84260)  Ax A8  (ADP18299)  *A*. *piechaudii* HLE (EJO33145)  Ax AXX-A  ( EGP47955) | Hypothetical gene (28%)  Carbamoyl-phosphate synthase large chain (22%)  Carbamoyl-phosphate synthase large chain (22%)  Carbamoyl-phosphate synthase large chain (22%) | AFK62060 | Carbamoyl-phosphate synthase large chain (25%) | ADU91191 | Carbamoyl-phosphate synthase large chain (25%) |
| NP_886679 | C-methyltransferase | NIL | NIL | NIL | NIL | Ax AXX-A  ( EGP44194)  *A*. *piechaudii* HLE *  Ax C54  Ax A8 | C-methyltransferase (26% with 25% less coverage)  NIL  NIL  NIL | NIL | NIL | NIL | NIL |
| NP_886680 | WbmS | NP_882488 | *wbmS* gene product (62%) | NIL | NIL | NIL | NIL | NIL | NIL | NIL | NIL |
| NP_886681 | WbmR | NP_882489 | *wbmR* gene product (64%) | NIL | NIL | NIL** | NIL | NIL | NIL | ADU91636 | Methionyl-tRNA formyltransferase (23%) |
| NP_886682 | WbmO | NP_882492 | *wbmO* gene product (82%) | NIL | NIL | NIL | NIL | NIL | NIL | NIL | NIL |
| NP_886683 | WbmN | NP_882493 | *wbmN* gene product (99%) | NIL | NIL | Ax C54 (EFV82085)  Ax AXX-A  *A*. *piechaudii* HLE  Ax A8 | ABC transporter ATP-binding protein (36% with 30% less coverage)  NIL  NIL  NIL | AFK64278 | Polysaccharide ABC transporter ATP-binding protein (33% with 35% less coverage) | ADU91936 | Teichoic acid export ATP-binding protein TagH (37% with 35% less coverage) |
| NP_886684 | WbmM | NP_882494 | *wbmM* gene product (100%) | NIL | NIL | Ax C54 (EFV82085)  Ax AXX-A  *A*. *piechaudii* HLE  Ax A8 | ABC transporter ATP-binding (39% with 25% less coverage)  NIL  NIL  NIL | NIL | NIL | NIL | NIL |
| NP_886685 | WbmL | NP_882495 | *wbmL* gene product (100%) | NIL | NIL | Ax C54 (EFV82084)  Ax AXX-A  *A*. *piechaudii* HLE  Ax A8 | ABC transporter ATP-binding (31%)  NIL  NIL  NI | NIL | NIL | ADU91935 | O-antigen export system permease RfbD (30%) |
| NP_886686 | WbmK | NP_882496 | *wbmK* gene product (99%) | NIL | NIL | NIL | NIL | NIL | NIL | NIL | NIL |
| NP_886687 | WbmJ | NP_882497 | *wbmJ* gene product (99%) | NIL | NIL | NIL | NIL | NIL | NIL | NIL | NIL |
| NP_886688 | WbmI | NP_882498 | *wbmI* gene product (100%) | NP_881711 | Asparagine synthase  (31%) | Ax C54  (EFV82083)  Ax A8  (ADP18911|)  *A*. *piechaudii* HLE  (EJO29518)  Ax AXX-A  (EGP47420) | Asparagine synthetase  (33%)  Asparagine synthase (glutamine-hydrolyzing) (29%)  Asparagine synthase (29%)  Asparagine synthase (29%) | AFK63834 | Asparagine synthase (29%) | ADU91940 | Asparagine synthetase (glutamine-hydrolyzing) (40%) |
| NP_886689 | WbmH | NP_882499 | *wbmH* gene product (100%) | NP_880358 | Capsular polysaccharide biosynthesis *wbpP* gene product (29%) | Ax C54  (EFV82082)  *A*. *piechaudii* HLE  (EJO29515)  Ax A8  (ADP18914)  Ax AXX-A  (EGP47423) | NAD dependent epimerase / dehydratase (31%)  VI polysaccharide biosynthesis gene VipB / TviC (28%)  VI polysaccharide biosynthesis gene VipB / TviC (28%)  VI polysaccharide biosynthesis gene VipB / TviC (28%) | AFK63837 | UDP-N-acetylglucosamine C4 epimerase (25%) | NIL | NIL |
| NP_886690 | WbmG | NP_882500 | *wbmG* gene product (100%) | NP_880358 | Capsular polysaccharide biosynthesis *wbpP* gene product (26%) | Ax C54  (EFV82082)  *A*. *piechaudii* HLE  (EJO30038)  Ax A8  (ADP17793)  Ax AXX-A  ( EGP48359 ) | NAD dependent epimerase / dehydratase (29%)  UDP-glucose 4-epimerase (28%)  UDP-glucose 4-epimerase (27%)  UDP-glucose 4-epimerase (28%) | AFK61421 | UDP-glucose 4-epimerase (30%) | NIL | NIL |
| NP_886691 | WbmF | NP_882501 | *wbmF* gene product (100%) | NP_880358 | Capsular polysaccharide biosynthesis *wbpP* gene product (27%) | Ax C54  (EFV82082)  *A*. *piechaudii* HLE  (EJO29515)  Ax AXX-A  (EGP48359)  Ax A8  ( ADP19595) | NAD dependent epimerase / dehydratase (29%)  VI polysaccharide biosynthesis gene VipB / TviC (27%)  VI polysaccharide biosynthesis gene VipB / TviC (27%)  dTDP-glucose 4,6-dehydratase (25%) | AFK61421 | UDP-glucose 4-epimerase (26%) | ADU91629 | dTDP-glucose 4,6-dehydratase |
| NP_886692 | WbmE | NP_882502 | *wbmE* gene product (99%) | NIL | NIL | NIL | NIL | NIL | NIL | NIL | NIL |
| NP_886693 | WbmD | NP_882503 | *wbmD* gene product (99%) | NIL | NIL | NIL | NIL | NIL | NIL | NIL | NIL |
| NP_886694 | WbmC | NP_882504 | *wbmE* gene product (99%) | NP_881711 | Asparagine synthase (32%) | Ax C54  (EFV82102)  Ax A8  (ADP18911)  *A*. *piechaudii* HLE  (EJO29518)  Ax AXX-A  (EGP47420) | Asparagine synthase (58%)  Asparagine synthase (33%)  Asparagine synthase (33%)  Asparagine synthase (33%) | AFK63834 | Asparagine synthase (34%) | ADU91940 | Asparagine synthase (30%) |
| NP_886695 | WbmB | NP_882505 | *wbmB* gene product (99%) | NIL | NIL | NIL | NIL | NIL | NIL | NIL | NIL |
| NP_886696 | WbmA | NP_882506 | *wbmA* gene product (99%) | NIL | NIL | NIL | NIL | NIL | NIL | NIL | NIL |

* Although no gene from strain HLE showed significant homology with this C-methyltransferase of Bb, a conserved hypothetical gene of *Achromobacter piechaudii* ATCC 43553 (EFF75886) did show 25% identity, albeit with a <80% coverage.

** Only one methionyl-tRNA formyltransferase (EFF78150) from *Achromobacter piechaudii* ATCC 43553 (but not HLE) has 24% identity.

**Table 2.** Genes in the second Bb locus (between nucleotide positions 929139 and 940614) that putatively encodes a lipopolysaccharide O-antigen, and their nearest homologs in closely related genomes. Potential orthologs are in blue font. Similar (having only low levels of identity) but phylogenetically diverged homologs (of apparently distinct origin and evolutionary history) are in magenta.

| ***Bb* RB50** | | ***Bpp* 12822** | | ***Bp* Tohama I** | | ***Achromobacter* spp.** | | ***Tk* WT001** | | ***Te* MCE9** | |
| --- | --- | --- | --- | --- | --- | --- | --- | --- | --- | --- | --- |
| **Protein_id** | **Name of the PEG** | **Protein_id** | **Name of the PEG**  **(% identity)** | **Protein_id** | **Name of the PEG**  **(% identity)** | **Protein_id** | **Name of the PEG** | **Protein_id** | **Name of the PEG**  **(% identity)** | **Protein_id** | **Name of the PEG**  **(% identity)** |
| NP_887424.1 | Glycosyltransferase | NP_883122.1 | Glycosyltransferase (100%) | NP_881716.1 | Glycosyltransferase (100%) | Ax A8  (ADP18916.1)  Ax C54  (EFV85830.1) | Glycosyl transferase family 4 family protein 1 (96%)  Undecaprenyl-phosphate α-N-acetylglucosaminyl 1-phosphate transferase (95%) | AFK63839.1 | Glycosyl transferase family 4 family protein 1 (78%) | ADU91257.1 | Phospho-N-acetylmuramoyl-pentapeptide- transferase (29%) |
| NP_887425.1 | WbpO  (polysaccharide biosynthesis protein) | NP_883123.1 | WbpO  (polysaccharide biosynthesis protein) (99%) | NP_881715.1 | WbpO  (polysaccharide biosynthesis protein) (98%) | Ax A8  (ADP18915.1)  Ax C54  (EFV85831.1) | VI polysaccharide biosynthesis protein VipA/TviB (89%)  Polysaccharide biosynthesis protein (89%) | AFK63838.1 | Polysaccharide biosynthesis protein (83%) | ADU91873.1 | UDP-glucose dehydrogenase (74%) |
| NP_887426.1 | UDP-N-acetylglucosamine C4 epimerase | NP_883124.1 | UDP-N-acetylglucosamine C4 epimerase (100%) | NP_881714.1 | UDP-N-acetylglucosamine C4 epimerase (99%) | Ax A8  (ADP18914.1)  Ax C54  (EFV85832.1) | VI polysaccharide biosynthesis protein VipA/TviB (82%)  UDP-N-acetylglucosamine C4 epimerase (82%) | AFK63837.1 | UDP-N-acetylglucosamine C4 epimerase (77%) | ADU91629.1 | dTDP-glucose 4,6-dehydratase (27%) |
| NP_887427.1 | Putative membrane protein | NP_883125.1 | Putative membrane protein (100%) | NP_881713.1 | Putative membrane protein (99%) | Ax A8  (ADP18913.1)  Ax C54  (EFV85834.1) | MviN-like family protein (85%)  Membrane protein (86%) | AFK63836.1 | Hypothetical protein TKWG_20435 (67%) | ADU92283.1 | Peptidoglycan lipid II flippase MurJ (23%) |
| NP_887428.1 | Glycosyltransferase | NP_883126.1 | Glycosyltransferase (100%) | NP_881712.1 | Glycosyltransferase (99%) | Ax A8  (ADP18912.1)  Ax C54  (EFV85835.1) | Glycosyltransferase (73%)  Glycosyltransferase (74%) | AFK63835.1* | Glycosyltransferase (61%) | NIL | NIL |
| NP_887429.1 | Asparagine synthase | NP_883127.1 | Asparagine synthase (100%) | NP_881711.1 | Asparagine synthase (99%) | Ax A8  (ADP18911.1)  Ax C54  (EFV85836.1) | Asparagine synthase (82%)  Asparagine synthase (82%) | AFK63834.1 | Asparagine synthase (60%) | ADU91940.1 | Asparagine synthase (30%) |
| NP_887430.1 | Glycosyltransferase | NP_883128.1 | Glycosyltransferase (99%) | NP_881710.1 | Glycosyltransferase (99%) | Ax A8  (ADP18910.1)  Ax C54  (EFV85837.1) | Glycosyl transferase group 1 (71%)  Glycosyltransferase (71%) | AFK63835.1* | Glycosyltransferase (41%) | NIL | NIL |
| NP_887431.1 | Glycosyltransferase | NP_883129.1 | Glycosyltransferase (99%) | NP_881709.1 | Glycosyltransferase (99%) | Ax A8  (ADP18909.1)  Ax C54  (EFV85807.1) | Glycosyltransferase group 1 (73%)  Glycosyltransferase (70%) | AFK63091.1 | Group 1 glycosyl transferase (26%) | NIL | NIL |
| NP_887432.1 | Glycosyltransferase | NP_883130.1 | Glycosyltransferase (99%) | NP_881708.1 | Glycosyltransferase (99%) | Ax A8  (ADP18908.1)  Ax C54  (EFV85808.1) | Glycosyltransferase group 1 (73%)  Glycosyltransferase (73%) | AFK61962.1 | Group 1 glycosyl transferase (29%) | NIL | NIL |

**Table 3.** Genes in the *wlb* and *waa* loci of Bb (spanning between nucleotide positions 123284 and 155070) and their nearest homologs in closely related genomes. Potential orthologs are in blue font. Similar (having only low levels of identity) but phylogenetically diverged homologs (of apparently distinct origin and evolutionary history) are in magenta.

| ***Bb* RB50** | | ***Bpp* 12822** | | ***Bp* Tohama I** | | ***Achromobacter* spp.** | | ***Tk* WT001** | | ***Te* MCE9** | |
| --- | --- | --- | --- | --- | --- | --- | --- | --- | --- | --- | --- |
| **Protein_id** | **Name of the PEG** | **Protein_id** | **Name of the PEG**  **(% identity)** | **Protein_id** | **Name of the PEG**  **(% identity)** | **Protein_id** | **Name of the PEG** | **Protein_id** | **Name of the PEG**  **(% identity)** | **Protein_id** | **Name of the PEG**  **(% identity)** |
| NP_886697 | *bplL* / *wlbL*-encoded lipopolysaccharide biosynthesis protein | NP_882507 | BplL / WlbL (99%) | NP_878986 | BplL / WlbL (99%) | Ax C54  (EFV82104)  Ax AXX-A  (EGP46656)  *A*. *piechaudii* HLE  (EJO31105)  Ax A8  (ADP19594) | BplL / WlbL (69%)  BplL / WlbL (69%)  BplL / WlbL (63%)  Polysaccharide biosynthesis protein (45% with 25% less coverage) | AFK63837 | UDP-N-acetyl glucosamine C4 epimerase (26% with 50% less coverage) | ADU91948 | UDP-N-acetylglucosamine 4,6-dehydratase (42% with 10% less coverage) |
| NP_886698 | *bplJK* / *wlbJK*-encoded hypothetical protein | NP_882508 | BplJK / WlbJK (100%) | No Protein_id  [Putative pseudogene] | BplJK / WlbJK (97%) | Ax C54  (EFV82105)  Ax AXX-A  (EGP46648)  Ax A8  *A*. *piechaudii* HLE | BplJK / WlbJK (44%)  BplJK / WlbJK (42%)  NIL  NIL | NIL | NIL | NIL | NIL |
| NP_886699 | *bplI* / *wlbI*-encoded  lipopolysaccharide biosynthesis protein | NP_882509 | BplI / WlbI (99%) | NP_878987 | BplI / WlbI (100%) | Ax C54  (EFV82106)  Ax AXX-A  (EGP46647)  Ax A8  *A*. *piechaudii* HLE | BplI / WlbI (69%)  BplI / WlbI (65%)  NIL  NIL | NIL | NIL | NIL | NIL |
| NP_886700 | *bplH* / *wlbH*-encoded glycosyl transferase family protein | NP_882510 | BplH / WlbH (99%) | NP_878988 | BplH / WlbH (99%) | Ax C54  (EFV82107)  Ax AXX-A  (EGP46646)  Ax A8  (ADP19498)  *A*. *piechaudii* HLE  (EJO30547) | BplH / WlbH (62%)  BplH / WlbH (54%)  Glycosyltransferase group 1 (30% with 50% less coverage)  Glycosyltransferase group 1 (30% with 50% less coverage) | NIL | NIL | NIL | NIL |
| NP_886701 | *bplG* / *wlbG*-encoded sugar transferase necessary for the synthesis of FucNAcMe, [Mutants fail to express the entire terminal trisaccharide] | NP_882511 | BplG / WlbG (100%) | NP_878989 | BplG / WlbG (98%) | Ax C54  (EFV82108)  Ax AXX-A  Ax A8  *A*. *piechaudii* HLE | BplG / WlbG (67%)  NIL  NIL  NIL | AFK61424 | Sugar transferase (34% with 35% less coverage) | ADU91944 | Glycosyltransferase (53%) |
| NP_886702 | *bplF* / *wlbF*-encoded  lipopolysaccharide biosynthesis protein | NP_882512 | BplF / WlbF (99%) | NP_878990 | BplF / WlbF (99%) | Ax C54  (EFV82109)  Ax AXX-A  (EGP46654)  *A*. *piechaudii* HLE  (EJO31103)  Ax A8  (ADP19581) | BplF / WlbF (80%)  BplF / WlbF (76%)  BplF / WlbF (35%)  Pleiotropic regulatory gene (30%) | NIL | NIL | ADU91943 | 4-keto-6-deoxy-N-acetyl-D-hexosaminyl-(Lipid carrier)  Aminotransferase / PglE (50%) |
| NP_886703 | *bplE* / *wlbE*-encoded glycosyl transferase family protein | NP_882513 | BplE / WlbE (99%) | NP_878991 | BplE / WlbE (99%) | Ax C54  (EFV82110)  *A*. *piechaudii* HLE  (EJO33196)  Ax AXX-A  (EGP46645)  Ax A8  (ADP19590) | BplE / WlbE (53%)  Lipopolysaccharides biosynthesis glycosyltransferase (42%)  BplE / WlbE (38%)  Glycosyltransferase group 1 (25%) | AFK64123 | Putative transferase (28% with 50% less coverage) | ADU91933 | Glycosyltransferase (31%) |
| NP_886704 | *bplD* / *wlbD*-encoded  UDP-N-acetylglucosamine 2-epimerase | NP_882514 | BplD / WlbD (99%) | NP_878992 | BplD / WlbD (99%) | *A*. *piechaudii* HLE  (EJO31104)  Ax AXX-A  (EGP46644)  Ax C54  (EFV83511)  Ax A8  (ADP19589)  Ax  (ADP15028) | UDP-N-acetylglucosamine 2-epimerase (72%)  UDP-N-acetylglucosamine 2-epimerase (71%)  UDP-N-acetylglucosamine 2-epimerase (36%)  UDP-N-acetylglucosamine 2-epimerase (33%)  UDP-N-acetylglucosamine 2-epimerase (36%) | AFK64277 | UDP-N-acetylglucosamine 2-epimerase (37%) | ADU91942 | UDP-N-acetylglucosamine 2-epimerase (49%) |
| NP_886705 | *bplC* / *wlbC*-encoded lipopolysaccharide biosynthesis protein [similar to DegT / DnrJ / EryC1 / StrS aminotransferase family] | NP_882515 | BplC / WlbC (100%) | NP_878993 | BplC / WlbC (99%) | *A*. *piechaudii* HLE  (EJO31103)  Ax C54  (EFV82111)  Ax AXX-A  (EGP46643)  Ax A8  (ADP19581) | BplC / WlbC (89%)  BplC / WlbC (91%)  BplC / WlbC (87%)  BplC / WlbC (59%) | NIL | NIL | ADU91934 | DegT/DnrJ/EryC1/StrS family protein encoding gene (53%) |
| NP_886706 | *bplB* / *wlbB*-encoded acetyltransferase | NP_882516 | BplB / WlbB (100%) | NP_878994 | BplB / WlbB (100%) | *A*. *piechaudii* HLE  (EJO31102)  Ax C54  (EFV82112)  Ax AXX-A  (EGP46642)  Ax A8  (ADP19580) | BplB / WlbB (86%)  BplB / WlbB (84%)  BplB / WlbB (84%)  BplB / WlbB (74%) | NIL | NIL | ADU91932 | Acetyltransferase / WbbJ (74%) |
| NP_886707 | *bplA* / *wlbA*-encoded  oxidoreductase family  NAD-binding Rossmann fold protein | NP_882517 | BplA / WlbA (100%) | NP_878995 | BplA / WlbA (100%) | *A*. *piechaudii* HLE  (EJO31101)  Ax C54  (EFV82113)  Ax AXX-A  (EGP46641)  Ax A8  (ADP19579) | BplA / WlbA (89%)  BplA / WlbA (89%)  Oxidoreductase gene (94%, albeit with 20% less coverage)  BplA / WlbA (22%) | AFK60821 | Oxidoreductase-like gene (24%) | ADU91931 | Oxidoreductase-like gene product (21%) |
| NP_886708 | *rfaC* / *waaC*-encoded heptosyltransferase | NP_882518 | RfaC / WaaC (99%) | NP_878996 | RfaC / WaaC (99%) | Ax A8  (ADP19574)  Ax AXX-A  (EGP46640)  Ax C54  (EFV82114)  *A*. *piechaudii* HLE  (EJO31100) | RfaC / WaaC (82%)  RfaC / WaaC (82%)  RfaC / WaaC (82%)  RfaC / WaaC (80%) | AFK63086 | Heptosyltransferase II (30%, with 50% less coverage) | ADU91616 | Lipopolysaccharide heptosyltransferase I (52%) |
| NP_886709 | *kdtA* / *waaA*-encoded  3-deoxy-D-manno-octulosonic-acid transferase | NP_882519 | KdtA / WaaA (99%) | NP_878997 | KdtA / WaaA (99%) | *A*. *piechaudii* HLE  (EJO31100)  Ax A8  (ADP19573)  Ax AXX-A  (EGP46639)  Ax C54  (EFV82115) | KdtA / WaaA (70%)  KdtA / WaaA (71%)  KdtA / WaaA (70%)  KdtA / WaaA (70%) | AFK61412 | KdtA / WaaA (50%) | ADU91617 | KdtA / WaaA (46%) |
|  | | | | | | | | | | | |
| NP_889925 | *waaF*-encoded heptosyltransferase II | NP_883992 | WaaF (100%) | NP_880960 | WaaF (100%) | Ax C54  (EFV87582)  Ax A8  (ADP15346)  Ax AXX-A  (EGP44754)  *A*. *piechaudii* HLE  (EJO30539) | WaaF (76%)  WaaF (77%)  WaaF (75%)  WaaF (74%) | AFK63086 | WaaF (53%) | ADU92256 | WaaF (44%) |

**Table 4.** Genes in the capsular polysaccharide biosynthesis and export locus of *Bb* (between nucleotide positions 3088087 and 3117841) and their nearest homologs in closely related genomes. Potential orthologs are in blue font. Similar (having only low levels of identity) but phylogenetically diverged homologs (of apparently distinct origin and evolutionary history) are in magenta.

| ***Bb* RB50** | | ***Bpp* 12822** | | ***Bp* Tohama I** | | ***Achromobacter* spp.** | | ***Tk* WT001** | | ***Te* MCE9** | |
| --- | --- | --- | --- | --- | --- | --- | --- | --- | --- | --- | --- |
| **Protein_id** | **Name of the PEG** | **Protein_id** | **Name of the PEG**  **(% identity)** | **Protein_id** | **Name of the PEG**  **(% identity)** | **Protein_id** | **Name of the PEG** | **Protein_id** | **Name of the PEG**  **(% identity)** | **Protein_id** | **Name of the PEG**  **(% identity)** |
| NP_889454 | WcbR / type I polyketide synthase | NP_885141 | *wcbR* gene product (99%) | NIL | NIL | *A*. *piechaudii* HLE (EJO33599)  Ax C54  ( EFV87141)  Ax AXX-A  (EGP48546)  Ax A8 | Polyketide synthase (61%)  Non-ribosomal peptide synthetase (36%)  Polyketide synthase (35%)  NIL | NIL | NIL | NIL | NIL |
| NP_889455 | WcbT / polyketide synthase | NP_884046 | 8-amino-7-oxononanoate synthase (39%, with 20% less coverage) | NP_880814 | 8-amino-7-oxononanoate synthase (39%, with 20% less coverage) | *A*. *piechaudii* HLE (EJO33600)  Ax AXX-A  (EGP48117)  Ax A8  (ADP17686)  Ax C54  (EFV84241) | Hypothetical protein QWC_01335 (76%)  8-amino-7-oxononanoate synthase (37%, with 20% less coverage)  Same as above  8-amino-7-oxononanoate synthase (38%, with 40% less coverage) | NIL | NIL | ADU91823 | 8-amino-7-oxononanoate synthase (31%, with 20% less coverage) |
| NP_889456 | WcbQ / capsular polysaccharide biosynthesis protein | NP_885142 | WcbQ (100%) | NP_880378 | WcbQ (99%) | *A*. *piechaudii* HLE  (EJO33618)  Ax C54  Ax AXX-A  Ax A8 | Sulfatase (56%)  NIL  NIL  NIL | NIL | NIL | NIL | NIL |
| NP_889457 | WcbP / oxidoreductase | NP_885143 | WcbP (100%) | NP_880379 | WcbP (99%) | *A*. *piechaudii* HLE (EJO33619)  Ax A8  (ADP18298)  Ax C54  (EFV86264)  Ax AXX-A  (EGP47953) | Short chain alcohol dehydrogenase of unknown specificity (63%)  Short chain dehydrogenase family protein 50 (35%)  Short chain dehydrogenase (36%)  Short chain dehydrogenase (35%) | AFK62062 | Short chain dehydrogenase (35%) | NIL | NIL |
| NP_889458 | WcbO / KpsS / capsular polysaccharide export protein | NP_885144 | WcbO (99%) | NIL | NIL | *A*. *piechaudii* HLE (EJO33605)  Ax C54  Ax AXX-A  Ax A8 | Capsule polysaccharide biosynthesis (49%)  NIL  NIL  NIL | AFK64282 | Capsule polysaccharide biosynthesis (35%) | NIL | NIL |
| NP_889459 | WcbA / KpsC / LipA / capsular polysaccharide export protein | NP_885145 | WcbA (99%) | NP_880359 | WcbA (99%) | *A*. *piechaudii* HLE (EJO33604)  Ax C54  Ax AXX-A  Ax A8 | Capsular polysaccharide export protein (54%)  NIL  NIL  NIL | AFK64260 | Capsule polysaccharide biosynthesis (43%) | NIL | NIL |
| NP_889460* | WbpP / TviC / VipB / capsular polysaccharide biosynthesis protein | NP_885146  NP_883124 | *wbpP* gene product (97%)  UDP-N-acetylglucosamine C4 epimerase (96%) | NP_880358  NP_881714 | *wbpP* gene product (96%)  UDP-N-acetylglucosamine C4 epimerase (96%) | Ax C54 (EFV85832)  Ax AXX-A  (EGP47423)  Ax A8 (ADP18914)  A. piechaudii HLE (EJO29515) | UDP-N-acetylglucosamine C4 epimerase (81%)  VI polysaccharide biosynthesis protein VipB/TviC (81%)  As above  VI polysaccharide biosynthesis protein VipB/TviC (80%) | AFK63837 | UDP-N-acetylglucosamine C4 epimerase (77%) | ADU91629 | dTDP-glucose 4,6-dehydratase (27%) |
| NP_889461** | WbpO / capsular polysaccharide biosynthesis protein | NP_885147  NP_883123 | *wbpO* gene product (99%)  *wbpO* gene product (99%) | NP_880357  NP_881715 | *wbpO* gene product (99%)  *wbpO* gene product (99%) | Ax C54 (EFV85831)  Ax AXX-A  (EGP47424)  Ax A8 (ADP18915)  *A*. *piechaudii* HLE (EJO29514) | Polysaccharide biosynthesis protein (89%)  VI polysaccharide biosynthesis protein VipA/TviB (90%)  VI polysaccharide biosynthesis protein VipA/TviB (89%)  VI polysaccharide biosynthesis protein VipA/TviB (88%) | AFK63838 | Polysaccharide biosynthesis protein (83%) | ADU91873 | UDP-glucose dehydrogenase (74%) |
| NP_889462 | Wza / WcbC / capsular polysaccharide export protein | NP_885148 | Wza / WcbC (100%) | NP_880356 | Wza / WcbC (99%) | *A*. *piechaudii* HLE (EJO33616)  Ax AXX-A  (EGP48363)  Ax C54 (EFV84040)  Ax A8 (ADP17797) | Outer membrane protein involved in polysaccharide (51%)  EPS I polysaccharide export outer membrane protein EpsA (34%)  Polysaccharide export protein (33%)  EPS I polysaccharide export outer membrane protein EpsA (32%) | AFK64280 | Outer membrane protein involved in polysaccharide (50%) | NIL | NIL |
| NP_889463 | VipC / capsular polysaccharide biosynthesis protein | NP_885149 | VipC (100%) | NP_880355 | VipC (100%) | NIL | NIL | NIL | NIL | NIL | NIL |
| NP_889464 | WbpT / capsular polysaccharide biosynthesis protein | NP_885150 | *wbpT* gene product (99%) | NP_880354 | *wbpT* gene product (99%) | Ax C54 (EFV85301)  Ax AXX-A  (EGP43833)  Ax A8 (ADP13597)  *A*. *piechaudii* HLE (EJO28864) | Glycosyltransferase (36%)  Glycosyltransferase group 1 protein (35%)  Glycosyltransferase group 1 protein (33%)  Glycosyltransferase group 1 (33%) | NIL | NIL | NIL | NIL |
| NP_889465 | WcbD / KpsE / capsule polysaccharide export inner-membrane protein | NP_885151 | *WcbD* / *kpsE* gene product (100%) | NP_880353 | *wcbD* / *kpsE* gene product (99%) | ? | ? | ? | ? | ? | ? |
| NP_889466 | KpsT / polysialic acid transport ATP-binding protein | NP_885152 | *kpsT* gene product (99%) | NP_880352 | *kpsT* gene product (98%) | Ax C54 (EFV82085)  Ax AXX-A  (EGP43836)  Ax A8 (ADP13600 )  *A*. *piechaudii* HLE | ABC transporter ATP-binding protein (32%)  ATPase component of an ABC polysaccharide exporter (24%, with 25% less coverage)  Same as above  NIL | AFK64278 | Polysaccharide ABC transporter ATP-binding protein (35%) | ADU91936 | Teichoic acid export ATP-binding protein TagH (26%) |
| NP_889467 | KpsM / polysialic acid transport protein | NP_885153 | *kpsM* gene product (100%) | NP_880351 | *kpsM* gene product (99%) | *A*. *piechaudii* HLE (EJO33615)  Ax A8  (ADP13599)  Ax C54  (EFV 85303)  Ax AXX-A  (EGP43835) | Permease of an ABC exporter involved in polysaccharide export (29%, with 10% less coverage)  ABC transporter (25%)  ABC transporter (25%)  ABC transporter (25%) | AFK64279 | Permease of an ABC exporter involved in polysaccharide export (26%) | ADU91935 | O-antigen export system permease protein RfbD (25%, with 20% less coverage) |
| NP_889468 | Glycosyltransferase | NP_885154 | Glycosyltransferase (99%) | NP_880350 | Glycosyltransferase (99%) | NIL | NIL | NIL | NIL | NIL | NIL |
| NP_889469 | Hypothetical protein | NP_885155 | Hypothetical protein (99%) | NP_880349 | Hypothetical protein (99%) | NIL | NIL | NIL | NIL | NIL | NIL |
| NP_889470 | TviD / capsular polysaccharide biosynthesis protein | NIL | NIL | NP_880348 | *tviD* gene product (99%) | NIL | NIL | NIL | NIL | NIL | NIL |

* This gene has another orthologous copy (UDP-N-acetylglucosamine C4 epimerase, NP_887426 having 96% identity) in the *Bordetella bronchiseptica* RB50 genome.

** This *wbpO* gene has another orthologous copy (NP_887425 having 99% identity) in the *Bordetella bronchiseptica* RB50 genome.

**References**

1. Van Iwaarden JF, Pikaar JC, Storm J, Brouwer E, Verhoef J, et al. (1994) Binding of surfactant protein A to the lipid A moiety of bacterial lipopolysaccharides. Biochem J 303 ( Pt 2): 407-411.

2. Schaeffer LM, McCormack FX, Wu H, Weiss AA (2004) Interactions of pulmonary collectins with Bordetella bronchiseptica and Bordetella pertussis lipopolysaccharide elucidate the structural basis of their antimicrobial activities. Infect Immun 72: 7124-7130.

3. Allen A, Maskell D (1996) The identification, cloning and mutagenesis of a genetic locus required for lipopolysaccharide biosynthesis in Bordetella pertussis. Mol Microbiol 19: 37-52.

4. Allen AG, Thomas RM, Cadisch JT, Maskell DJ (1998) Molecular and functional analysis of the lipopolysaccharide biosynthesis locus wlb from Bordetella pertussis, Bordetella parapertussis and Bordetella bronchiseptica. Mol Microbiol 29: 27-38.

5. Allen AG, Isobe T, Maskell DJ (1998) Identification and cloning of waaF (rfaF) from Bordetella pertussis and use to generate mutants of Bordetella spp. with deep rough lipopolysaccharide. J Bacteriol 180: 35-40.

6. Preston A, Allen AG, Cadisch J, Thomas R, Stevens K, et al. (1999) Genetic basis for lipopolysaccharide O-antigen biosynthesis in bordetellae. Infect Immun 67: 3763-3767.

7. Preston A, Maxim E, Toland E, Pishko EJ, Harvill ET, et al. (2003) Bordetella bronchiseptica PagP is a Bvg-regulated lipid A palmitoyl transferase that is required for persistent colonization of the mouse respiratory tract. Mol Microbiol 48: 725-736.

8. Plainvert C, Bidet P, Peigne C, Barbe V, Medigue C, et al. (2007) A new O-antigen gene cluster has a key role in the virulence of the Escherichia coli meningitis clone O45:K1:H7. J Bacteriol 189: 8528-8536.
