# Supplementary material for: Genome Implosion Elicits Host-Confinement in Alcaligenaceae: Evidence from the Comparative Genomics of Tetrathiobacter kashmirensis, a Pathogen in the Making
Source: PLoS One. 2013 May 31;8(5):e64856. doi: 10.1371/journal.pone.0064856 (PMC3669393; doi:10.1371/journal.pone.0064856)
Supplement: File S5 — Comparative genomics of the iron uptake capabilities of the four Alcaligenaceae . (DOC) [file pone.0064856.s005.doc]

**Comparative genomics of iron uptake mechanisms of Alcaligenaceae**

Iron uptake mechanisms, central to bordetellar virulence, are well developed across *Alcaligenaceae*. Most pathogenic bacteria live in perpetually iron impoverished environment, and hence develop versatile mechanisms for iron acquisition and metabolism [1]. Since most of the iron in the pathogens’ habitat remains complexed with proteins, their survival entails specialization in obtaining iron from iron-containing host proteins [1]. This is achieved by contact-dependent or contact-independent mechanisms, both of which involve specialized outer membrane receptors that either directly bind host iron sources (such as lactoferrin, transferrin, ferritin, hemes and hemoproteins) or bind to and transport iron bound to siderophores [2]. Accordingly, bacteria have wide arrays of surface receptors for direct uptake of heme, or irons from transferrin, lactoferrin etc. [3]. They may also synthesize siderophores in tandem with their cognate transporters and/or only transporters corresponding to specific xenosiderophores [4]. TonB-dependent outer membrane receptors transfer iron chelates and heme to the periplasm, subsequent to which they are transported to the cytoplasm by ABC transporters. In the absence of TonB, these receptors bind their substrates but do not render active transport [5]. The proton motive force of the cytoplasmic membrane transports host-derived Fe or heme across the outer membrane by means of the TonB-ExbB-ExbD proteins [6].

*Bb*, *Bp* and *Bpp* all produce and utilize the siderophore alcaligin, in addition to which *Bb* and *Bp* utilize xenosiderophores like enterobactin, ferrichrome, and desferrioxamine B [7]. *Bb*, again, is versatile enough to be able to use aerobactin, ferrichrysin, ferricrocin, ferrirubin, protochelin, schizokinen, vicibactin, and pyoverdin [7]. What is more, it can further utilize mammalian transferrins and hemes that are released from injured host cells in course of extensive host-pathogen interactions. *Bb*’s multifaceted ability to detect and utilize miscellaneous iron sources may be one of the keys to its successful adaptation to diverse host environments offering variable iron source availabilities.

Out of the various iron utilization systems only those meant for alcaligin, enterobactin and heme have been characterized in detail. All these mechanisms are reportedly repressed by the ferric uptake regulation protein Fur (or functionally similar repressors) [8] and activated under iron starvation conditions by mechanisms involving dedicated positive regulators inducible by the cognate iron source [7]. Iron starvation, again, is a key signal controlling the expression of a host of virulence factors in different bacteria [9,10] including the pathogenic bordetellae [7]. As such, a whole gamut of factors ranging from the iron-starved condition itself [11] to the successful expression of tonB [12], biosynthesis of alcaligin siderophore [13], its receptors [14] as well as those for enterobactin, [15], heme [16] etc. have been proven as essential for the successful colonization and persistence of *Bb*, *Bp* and *Bpp* in diverse host cellular environments. In addition to those responsible for the utilization of alcaligin, enterobactin and heme, iron-restricted environments of their hosts further elicit a wide range of bordetellar gene expression responses. Such responses include repression of T3SS genes by iron, expression of the iron-repressed cytoplasmic membrane transporter system fbpABC (required for the utilization of multiple structurally distinct siderophores such as alcaligin, enterobactin, ferrichrome, and desferrioxamine B) under iron-starved conditions [7].

*Bb*, *Bp* or *Bpp* have around 14 to 19 predicted or experimentally verified TonB-dependent ferric complex receptor genes [7,17], while A8 has at least 25 of them in addition to 6 putative TonB-dependent receptor plug domain genes. *Tk*, in its turn, has 15 putative genes (including one pseudogene) for TonB-dependent Fe^3+^ ion receptors (see Table 1 below). The abridged genome of *Te* also has five TonB-dependent outer membrane receptors, some of which are putatively specific for lactoferrin / transferrin and heme. The syntenic *tonB*-*exbB*-*exbD* genes are apparently conserved in the family, even as the *exbB* homolog of *Tk* may have been pseudogenized. This potential loss may anyway get replenished by another *tolQ*-*tolR* (homologous to *exbB*-*exbD*) locus present in *Tk*, as well as A8 and *Bb*.

All the genes necessary for pyoverdin biosynthesis and transport were identifiable in the A8 genome, even as there was no clue about the chemical nature of the siderophore(s) potentially synthesized by *Tk*. Nonetheless, quite a few genes putatively involved in the biosynthesis of siderophore-like substances are present in the *Tk* genome (see Table 2 below) in conjunction with receptors and/or transporters for heme, hemin, ferrichrome, achromobactin, ferritin, enterobactin, pyoverdin etc. Putatively under the control of the FUR regulon [8], *Tk* further encompass a Fe^2+^-dicitrate sensor and a specialized RNA polymerase sigma subunit (sigma24) co-localized with a TonB-dependent siderophore receptor. No siderophore biosynthesis-related gene is there in the *Te* genome, albeit three genes each for the transport of Fe^3+^ iron and hemin are present. A8, *Bb* and *Tk*, on the other hand possess elaborate heme, hemin uptake, transport and utilization systems (see Table 3 below). Again, at least one copy of fbpA, fbpB and fbpC present in all the compared genomes, including Te, reaffirming the ingrained ability of this group to utilize diverse siderophores from the environment.

**Table 1. Putative TonB-dependent ferric complex receptors in the *Tk* genome**

| **Locus** | **Gene product** | **Predicted**  **function** | **Nearest homolog**  **(% identity)** | **Nearest bordetellar homolog (% identity)** |
| --- | --- | --- | --- | --- |
| TKWG_00935 | TonB-dependent receptor, plug | COG1629 associated with Fe transport | TonB-dependent receptor, plug of many *Burkholderia* spp. (50%) | Exogenous ferric siderophore receptor (CAE35124) of *Bb* RB50 (34%) |
| TKWG_01475 | TonB-dependent OM receptor | COG1629 associated with Fe transport | TonB-dependent OM receptor (CAP44996) of *Bordetella petri* (59%);    Homolog from *Bb* RB50 (CAE31331) (57%) | TonB-dependent OM receptor (CAP44996) of *Bordetella petri* (59%);  Homolog from *Bb* RB50 (CAE31331) (57%) |
| TKWG_01480 | TonB-dependent OM receptor | COG1629 associated with Fe transport | TonB-dependent receptor (AEC21636) of *Pusillimonas* sp. T7-7 (64%) | TonB-dependent receptor (CAE31331) of *Bb* RB50 (60%) |
| TKWG_01485 | TonB-dependent OM receptor | COG1629 associated with Fe transport | TonB-dependent receptor (AEC21636) of *Pusillimonas* sp. T7-7 (61%) | TonB-dependent receptor (CAE31331) of *Bb* RB50 (58%) |
| TKWG_08605 | putative TonB-dependent receptor | COG4773 OM receptor for ferric coprogen and ferric-rhodotorulic acid siderophore | FpvA (CAL95128) of *Azoarcus* sp. BH72 (48%) | TonB-dependent OM receptor (CAP43540) of *Bordetella petrii* DSM 12804 (38%) |
| TKWG_08710 | TonB-dependent ferric siderophore receptor | COG1629 OM receptor protein associated with Fe transport | TonB-dependent siderophore receptor (YP_609109) of *Pseudomonas entomophila*  L48 (53%) | Ferric siderophore receptor (NP_880686) of *Bordetella* *pertussis* Tohama I (52%) |
| TKWG_08715 | TonB-dependent ferric siderophore receptor | COG1629 OM receptor protein associated with Fe transport | Ferrichrome receptor (ZP_16183048) of Cupriavidus necator HPC(L) (43%) | Ferric siderophore receptor (NP_884632) of *B*. *parapertussis* 12822 (37%)  Ferric siderophore receptor (NP_888391) of *B*. bronchiseptica RB50 (37%) |
| TKWG_08720 | TonB-dependent ferric siderophore receptor | COG1629 OM receptor protein associated with Fe transport | (YP_002258073) Ferric siderophore receptor of *Ralstonia solanacearum* IPO1609 (51%)  Ferric siderophore receptor (CCA81688) of Blood disease bacterium R229 (51%) | TonB-dependent outer membrane receptor (YP_001632827) of *B*. *petrii* DSM 12804 (44%) |
| TKWG_12295 | TonB-dependent ferric siderophore receptor | COG1629 OM receptor protein associated with Fe transport | TonB-dependent siderophore receptor (ZP_07045434) of *Comamonas testosteroni* S44 (65%)  Ferric alcaligin E (ZP_15932409) of Achromobacter piechaudii HLE (60%) | TonB-dependent outer membrane receptor (YP_001633263) of *B*. *petrii* DSM 12804 (25%) |
| TKWG_18375 | putative TonB-dependent receptor | COG1629 OM receptor protein associated with Fe transport | TonB-dependent outermembrane ferrioxamine receptor (ZP_15928561) of Achromobacter piechaudii HLE (53%) | Ferric siderophore receptor (NP_890193) of *B*. bronchiseptica RB50 (38%) and a host of other *Bordetella* species. |
| TKWG_18825 | TonB-dependent ferric siderophore receptor | COG1629 OM receptor protein associated with Fe transport | TonB-dependent siderophore receptor, partial (ZP_16186538) of Cupriavidus necator HPC(L) (70%) | TonB-dependent outer membrane receptor (YP_001632827) of *B*. *petrii* DSM 12804 (58%) |
| TKWG_18835 | TonB-dependent ferric siderophore receptor | COG1629 OM receptor protein associated with Fe transport | TonB-dependent siderophore receptor, partial (ZP_16186538) of *Cupriavidus necator*  HPC(L) (61%)  (ZP_09299843) TonB-dependent siderophore receptor [Achromobacter arsenitoxydans  SY8] (60%) | (YP_001632827) TonB-dependent outer membrane receptor of *B*. petrii DSM 12804 (40%) |
| Nt. positions 3365165 - 3367158) | TonB-dependent ferric achromobactin receptor | Pseudogene | Not determined | Not determined |
| TKWG_21110 | TonB-dependent iron/copper receptor | COG1629 OM receptor protein associated with Fe transport | TonB-dependent copper receptor (YP_003979205) of *Achromobacter xylosoxidans* A8 (71%) | TonB-dependent outer membrane receptor (YP_001632451) of Bordetella petrii DSM  12804 (69%) |
| TKWG_22815 | putative TonB-dependent receptor | Unknown function | Outer membrane protein (ZP_10347816) of *Alcaligenes* *faecalis* subsp. *faecalis* NCIB 8687  (43% identity over only 60% coverage in the C-terminal end) | TonB-dependent vitamin B12 receptor (YP_787562) of *B*. *avium* 197N (41%) |

**Table 2. Putative siderophore biosynthesis and transport genes of *Tk***

| **Locus** | **Gene Product** | **Predicted**  **Function** | **Nearest Homolog** | **Bordetellar Homolog** |
| --- | --- | --- | --- | --- |
| TKWG_00940 | COG0614 ABC-type Fe^3+^-hydroxamate transport system, periplasmic component | Periplasmic binding protein | Periplasmic binding protein (YP_486309) of *Rhodopseudomonas palustris* HaA2 (38%) | None |
| TKWG_00950 | COG0609 ABC-type Fe3+-siderophore transport system, permease component | ABC transporter | ABC transporter permease (NP_881873) of *B*. *pertussis* Tohama I (61%) | ABC transporter permease (NP_881873) of *B*. *pertussis* Tohama I (61%) |
| Nt. positions  163804 - 164873 | Pseudogene | FAD-binding 9 siderophore-interacting domain-containing protein | Not determined | Not determined |
| TKWG_00975 | Putative export protein | Siderophore export | Siderophore export protein (YP_002762736) of *Gemmatimonas aurantiaca* T-27 (47% identity over ~62% coverage in the C-terminal end) | Transporter (YP_785300) of *B*. *avium* 197N (47% identity over only ~50% coverage in the C-terminal end) |
| TKWG_00980 | COG0477 Permeases of the major facilitator superfamily | Siderophore export | Siderophore export protein (YP_002762736) of *Gemmatimonas aurantiaca* T-27 (38%) | Transporter (YP_785300) of *B*. *avium* 197N (27%) |
| TKWG_02450 | COG1840 Fe^3+^ transport system, periplasmic component | ABC transporter | Ferric iron ABC transporter, iron-binding protein (YP_004874797) of *Taylorella asinigenitalis* MCE3 (68%) | ABC transporter, periplasmic binding protein (YP_785715) of *B*. *avium* 197N (34%) |
| TKWG_08610 | COG3486 Lysine / ornithine N-monooxygenase | Plausibly involved in alcaligin biosynthesis | Alcaligin biosynthesis enzyme (ZP_16399225) of *Achromobacter xylosoxidans* C54 | Alcaligin biosynthesis enzyme (NP_881083) of *B*. *pertussis* Tohama I (46%)  Alcaligin biosynthesis enzyme (NP_885604) of *B*. *parapertussis* 12822 (46%) |
| TKWG_08685 | COG1108 permease component encountered in pyoverdin gene cluster | ABC transporter | Permease of ABC transporter (ZP_15637997) of *Pseudomonas aeruginosa* E2 (77%) | ABC transporter permease (NP_886735) of *B*. bronchiseptica RB50 (30%) |
| TKWG_18455 | COG0609 ABC-type Fe3+-siderophore transport system, permease component | ABC transporter | Transporter permease (ZP_09302722) of *Achromobacter arsenitoxydans* SY8 (67%) | Hemin ABC transporter permease (YP_787626) of *B*. *avium* 197N (66%) |
| TKWG_19125 | COG1120 ABC-type cobalamin/Fe3+-siderophores  transport systems, ATPase components | Ferrichrome transport ATP-binding protein FhuC | ABC transporter-related protein (ZP_07544161) of *Actinobacillus pleuropneumoniae*  serovar 12 str. 1096 (52%) | Iron ABC transporter ATP-binding protein (YP_787152) of *B*. *avium* 197N (37%) |
| TKWG_19135 | COG0614 ABC-type Fe3+-hydroxamate transport system, periplasmic component | Periplasmic binding protein | Putative ferrichrome-binding periplasmic protein FhuD (ZP_05320060) of *Neisseria sicca* ATCC 29256 (34%) | None |
| TKWG_19140 | COG0609 ABC-type Fe3+-siderophore transport system, permease component | Iron-hydroxamate transporter permease subunit | Putative permease component of ABC-type hydroxamate-dependent iron transport system (ZP_01226568) of *Aurantimonas manganoxydans* SI85-9A1 (41%) | None |
| TKWG_19145 | COG0609 ABC-type Fe3+-siderophore transport system, permease component | Iron-hydroxamate transporter permease subunit | FecCD transporter (ZP_16221813) of Serratia plymuthica A30 (38%) | None |
| TKWG_19150 | COG0609 ABC-type Fe3+-siderophore transport system, permease component | Iron-hydroxamate transporter permease subunit | Truncated peptide with 48% identity with transporter permease (YP_003695529) of *Starkeya novella* DSM 506 | None |
| TKWG_19560 | COG0609 ABC-type Fe3+-siderophore transport system, permease component | Ferric achromobactin inner membrane permease | 53% identity with Ferric achromobactin inner membrane permease (YP_003882395) of *Dickeya dadantii* 3937  and CbrB protein (CAA60667) of *Erwinia chrysanthemi* | None |
| TKWG_19565 | COG0609 ABC-type Fe3+-siderophore transport system, permease component | Enterobactin ABC transporter permease | Enterobactin ABC transporter permease (YP_434629) of *Hahella chejuensis* KCTC  2396 (45%) | Hemin ABC transporter permease (YP_787626) of *B*. *avium* 197N  (34%) |
| TKWG_19570 | COG1120 ABC-type cobalamin/Fe3+-siderophores transport systems, ATPase components | ABC transporter | ABC transporter (YP_003260293) of *Pectobacterium wasabiae* WPP163]  (67%) | None |
| TKWG_19575 | COG4114 Uncharacterized Fe-S protein | FhuF/siderophore biosynthesis protein | Hypothetical protein of (ZP_11287460) *Pseudomonas viridiflava* UASWS0038 (49%) | None |
| TKWG_22445 | COG0803 ABC-type metal ion transport system, periplasmic component/surface adhesin | Priplasmic chelated iron-binding protein | Periplasmic chelated iron-binding protein (ZP_12978884) of *Agrobacterium tumefaciens* 5A (54%) | None |
| TKWG_23655 | COG2375 Siderophore-interacting protein | Siderophore-interacting protein | Siderophore-interacting protein (YP_002007762) of *Cupriavidus taiwanensis* LMG 19424 (56%) | Iron utilization protein (NP_884897) of *B*. *parapertussis* 12822 |
| TKWG_24750 | COG4321 uncharacterized protein involved in siderophore biosynthesis | Putative arylsulfate sulfotransferase | Hypothetical protein (YP_283253) Daro_0024 *Dechloromonas aromatica* RCB (58%) | Hypothetical protein (YP_785434) of *B*. *avium* 197N (52%) |

**Table 3.** Comparison of the putative heme, hemin uptake, transport and utilization genes identified in the four genomes

| **Functional identity of the PEG** | **A8** | ***Tk*** | ***Bb*** | ***Te*** |
| --- | --- | --- | --- | --- |
| Heme oxygenase HemO, associated with heme uptake | + | - | - | - |
| Ferric siderophore transport system, periplasmic binding protein TonB | + | - | + | + |
| Ferric reductase | + | - | - | - |
| Paraquat-inducible protein B | + | + | + | - |
| Heme uptake regulator | + | + | + | - |
| Heme uptake transmembrane sensor | + | - | + | - |
| ABC-type hemin transport system, ATPase component | + | + | + | - |
| Periplasmic hemin-binding protein | + | + | + | - |
| Outer membrane receptor proteins, mostly Fe transport | + | + | - | + |
| Hemin ABC transporter, permease protein | + | + | + | - |
| Paraquat-inducible protein A | + | + | + | - |
| Hemin transport protein HmuS | + | + | + | - |
| TonB-dependent hemin , ferrichrome receptor | + | + | + | - |
| Pyridoxamine 5'-phosphate oxidase-related putative heme iron utilization protein | - | + | - | - |
| Ferrichrome transport ATP-binding protein FhuC | - | + | - | - |
| Ptative Cytochrome bd2, subunit I | - | - | + | - |
| Ptative Cytochrome bd2, subunit II | - | - | + | - |
| Cytochrome c-type biogenesis protein DsbD, protein-disulfide reductase | - | - | + | - |
| Predicted iron-dependent peroxidase, Dyp-type family | - | - | + | - |
| Encapsulating protein for a DyP-type peroxidase or ferritin-like protein oligomers | - | - | + | - |
| Electron transfer flavoprotein, beta subunit | - | - | - | + |

**References**

1. Eppinger M, Baar C, Raddatz G, Huson DH, Schuster SC (2004) Comparative analysis of four Campylobacterales. Nat Rev Microbiol 2: 872-885.

2. Miethke M, Marahiel MA (2007) Siderophore-based iron acquisition and pathogen control. Microbiol Mol Biol Rev 71: 413-451.

3. Ratledge C, Dover LG (2000) Iron metabolism in pathogenic bacteria. Annu Rev Microbiol 54: 881-941.

4. Neilands JB (1995) Siderophores: structure and function of microbial iron transport compounds. J Biol Chem 270: 26723-26726.

5. Koebnik R (2005) TonB-dependent trans-envelope signalling: the exception or the rule? Trends Microbiol 13: 343-347.

6. Braun V (2003) Iron uptake by Escherichia coli. Front Biosci 8: s1409-1421.

7. Brickman TJ, Cummings CA, Liew SY, Relman DA, Armstrong SK (2011) Transcriptional profiling of the iron starvation response in Bordetella pertussis provides new insights into siderophore utilization and virulence gene expression. J Bacteriol 193: 4798-4812.

8. Stojiljkovic I, Baumler AJ, Hantke K (1994) Fur regulon in gram-negative bacteria. Identification and characterization of new iron-regulated Escherichia coli genes by a fur titration assay. J Mol Biol 236: 531-545.

9. Mekalanos JJ (1992) Environmental signals controlling expression of virulence determinants in bacteria. J Bacteriol 174: 1-7.

10. Litwin CM, Calderwood SB (1993) Role of iron in regulation of virulence genes. Clin Microbiol Rev 6: 137-149.

11. Vidakovics ML, Lamberti Y, Serra D, Berbers GA, van der Pol WL, et al. (2007) Iron stress increases Bordetella pertussis mucin-binding capacity and attachment to respiratory epithelial cells. FEMS Immunol Med Microbiol 51: 414-421.

12. Pradel E, Guiso N, Menozzi FD, Locht C (2000) Bordetella pertussis TonB, a Bvg-independent virulence determinant. Infect Immun 68: 1919-1927.

13. Register KB, Ducey TF, Brockmeier SL, Dyer DW (2001) Reduced virulence of a Bordetella bronchiseptica siderophore mutant in neonatal swine. Infect Immun 69: 2137-2143.

14. Brickman TJ, Armstrong SK (2007) Impact of alcaligin siderophore utilization on in vivo growth of Bordetella pertussis. Infect Immun 75: 5305-5312.

15. Brickman TJ, Hanawa T, Anderson MT, Suhadolc RJ, Armstrong SK (2008) Differential expression of Bordetella pertussis iron transport system genes during infection. Mol Microbiol 70: 3-14.

16. Brickman TJ, Vanderpool CK, Armstrong SK (2006) Heme transport contributes to in vivo fitness of Bordetella pertussis during primary infection in mice. Infect Immun 74: 1741-1744.

17. Sebaihia M, Preston A, Maskell DJ, Kuzmiak H, Connell TD, et al. (2006) Comparison of the genome sequence of the poultry pathogen Bordetella avium with those of B. bronchiseptica, B. pertussis, and B. parapertussis reveals extensive diversity in surface structures associated with host interaction. J Bacteriol 188: 6002-6015.
